# Supplementary material for: Identifying interpretable gene-biomarker associations with functionally informed kernel-based tests in 190,000 exomes
Source: Nat Commun. 2022 Sep 10;13:5332. doi: 10.1038/s41467-022-32864-2 (PMC9464252; doi:10.1038/s41467-022-32864-2)
Supplement: Supplementary file 1 — Supplementary Information [file 41467_2022_32864_MOESM1_ESM.pdf]

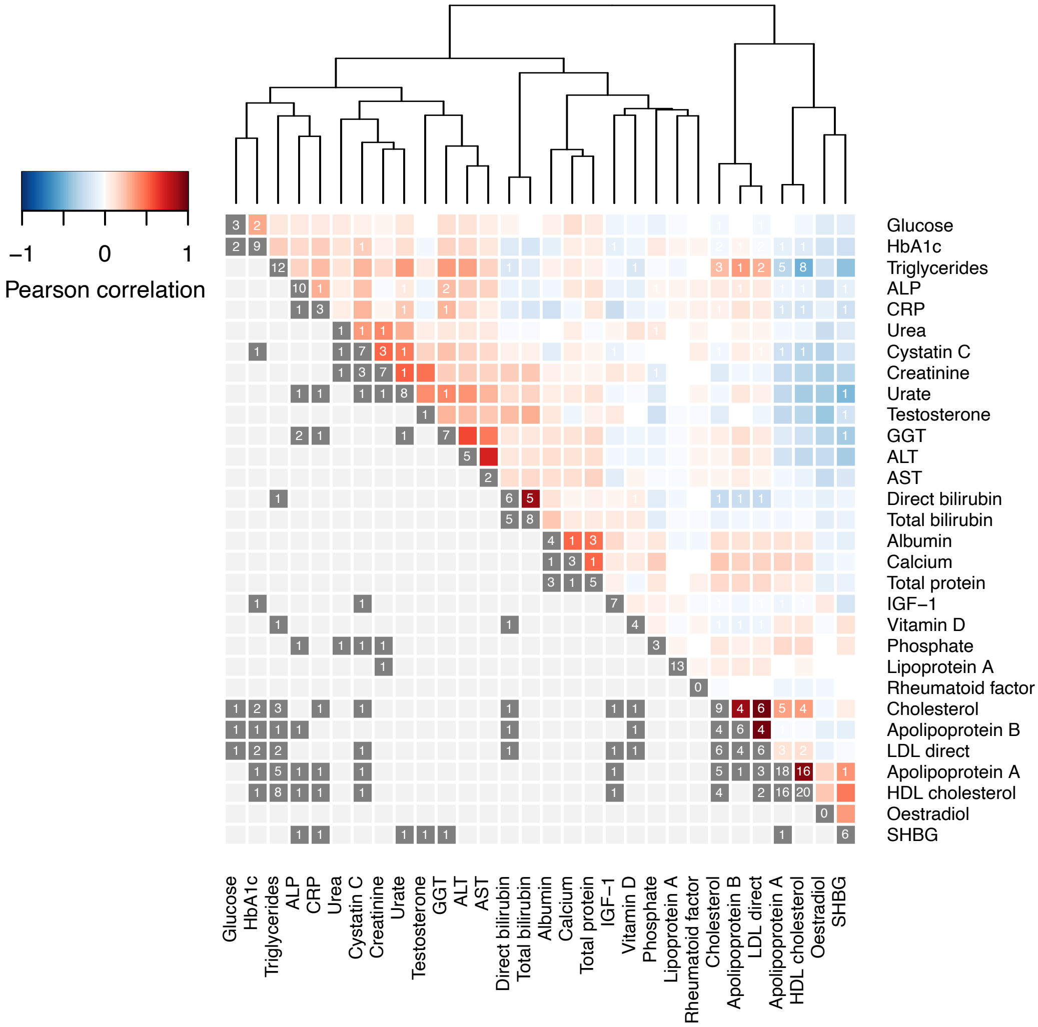

Supplementary Figure 1: **Biomarker correlation and number of hits.** Heatmap showing Pearson correlation between pre-processed biomarkers (upper triangle) and number of significant associations (cell notes). Rows and columns are clustered using complete linkage on the Euclidean distances of the correlation matrix between phenotypes (dendrogram). While some even weakly (anti-)correlated biomarkers share significant associations (e.g., Cholesterol and Glucose, gene: *GIGYF1*), other highly correlated markers do not share significant associations (e.g., GGT, ALT, AST).

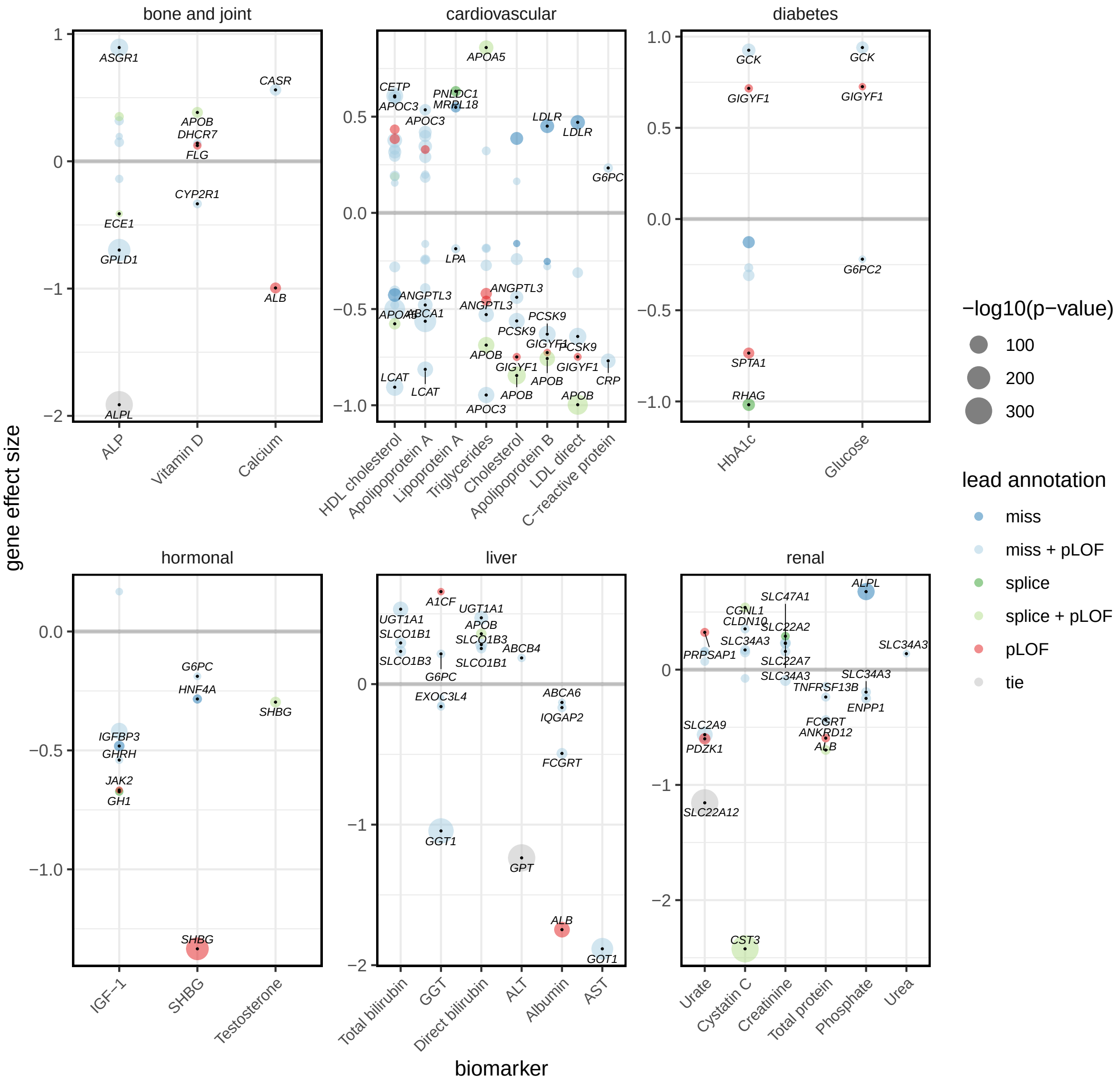

Supplementary Figure 2: **Gene-based variant collapsing results overview.** Collapsing variants allows defining gene effect sizes. Bubble plots showing the effect sizes after weighted collapsing of variants (y-axis) of significant associations (FWER ≤ 0.05) for each biomarker (x-axis). The four genes with largest absolute effect sizes are labeled for each biomarker. Larger bubble size indicates higher significance. P-values and effect sizes are those given by the most significant variant effect category (lead annotation). In case of ties ($p = 0$, gray) the average effect size across annotations is shown. Effect sizes are calculated on covariate-corrected quantile transformed phenotypes. Data are available in Supplementary Data 1.

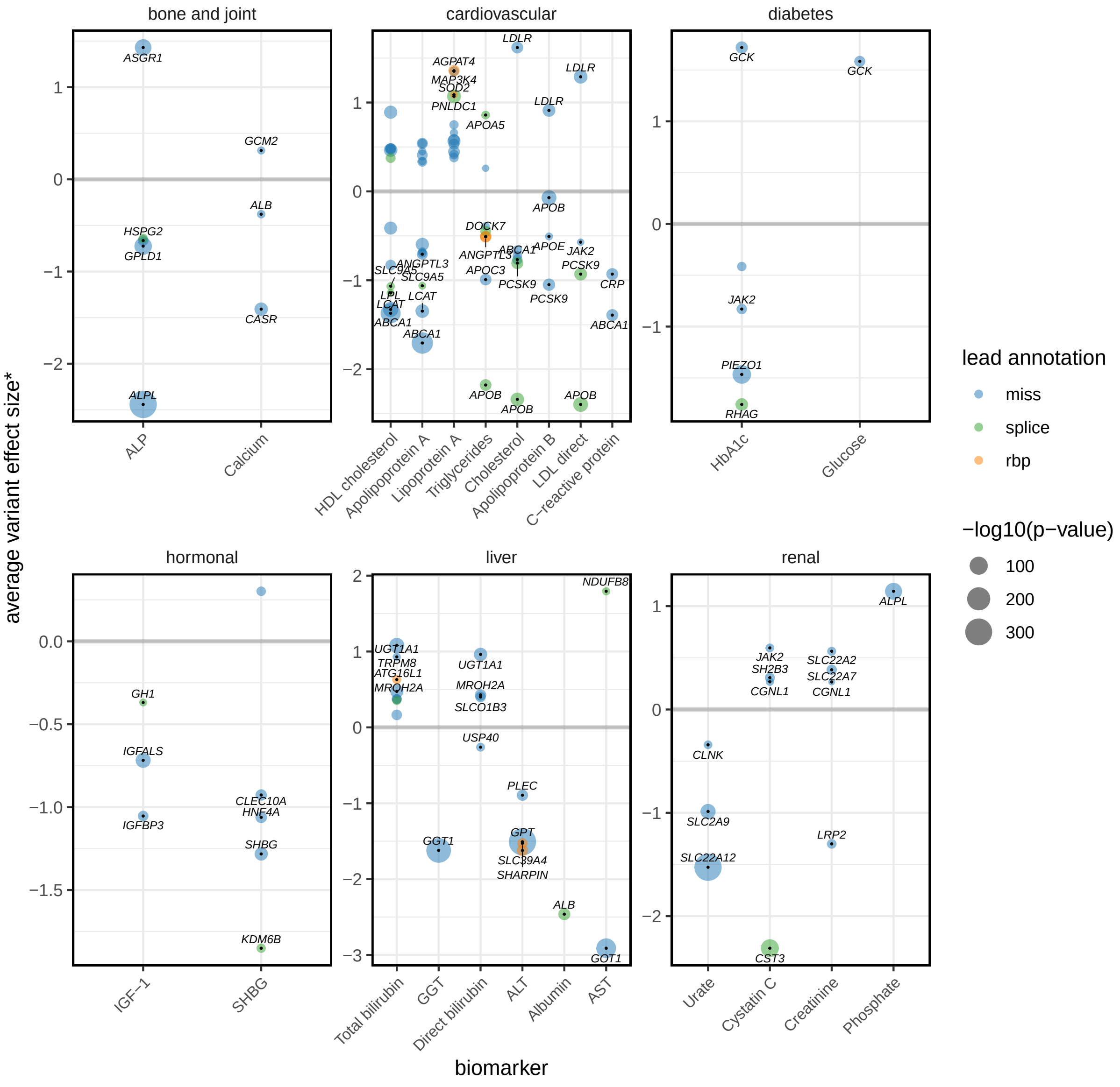

Supplementary Figure 3: **Kernel-based tests results overview.** We calculated the average effect size for *variants with single-variant p-values below 10^{-5} (score test) within significant genes (FWER ≤ 0.05) found by kernel-based tests if the cumulative minor allele count across these variants was at least 5. Bubble plots showing these average effect sizes (y-axis) for each biomaker (x-axis). The four genes with largest average effect sizes are labeled for each biomaker. Larger bubble size indicates higher significance of the gene-based test. P-values and average effect sizes are those given by the most significant variant effect category (lead annotation). Effect sizes are calculated on covariate-corrected quantile transformed phenotypes. Variant weights were not considered in the calculation of single-variant effect sizes. Data are available in Supplementary Data 1.

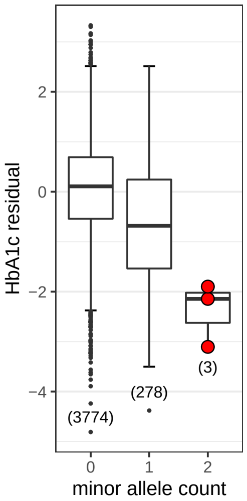

Supplementary Figure 4: ***PIEZO1* L2277M, (16:88716656:G:T).** Dosage plot of *PIEZO1* L2277M in individuals of inferred South Asian ancestry. Numbers in brackets denote the number of carriers of 0, 1 or 2 minor alleles (x-axis), from left to right: $n_0 = 3773$, $n_1 = 278$ and $n_2 = 3$. The three only homozygous carriers are shown in red. The y-axis contains the covariate-adjusted quantile transformed values for HbA1c. Centers denote the medians. The lower and upper hinges indicate 25th and 75th percentiles. Whiskers extend to the largest/lowest values no further than 1.5 $\times$ IQR away from the upper/lower hinges and black points denote outliers. Data ranged from -4.81 (min) to 3.3 (max).

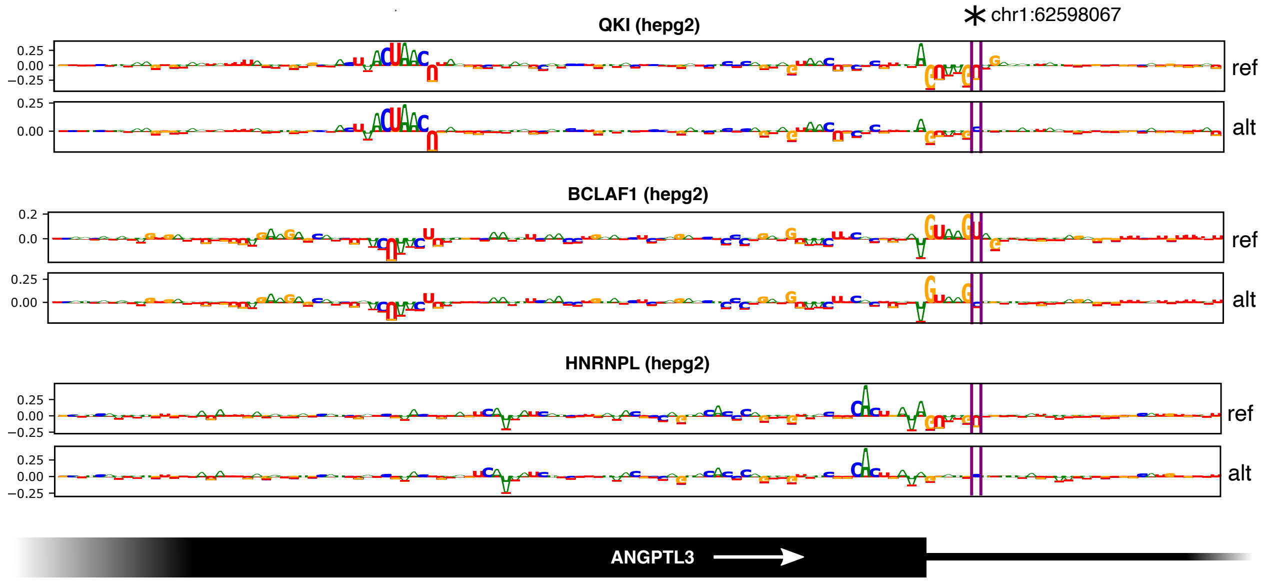

Supplementary Figure 5: **Attribution maps.** The variant 1:62598067:T:C at one-based position chr1:62598067 makes DeepRiPe predict increased binding probabilities for HNRNPL and QKI, and decreased probability for BCLAF1. Attribution maps for reference (ref) and alternative (alt) sequences as described in [1] highlight important nucleotides proximal to an ANGPTL3 exon boundary. The predictions for QKI depend positively on an upstream QKI binding motif (ACUAAC), and negatively on the splice donor signal (GUAAGU). The pattern is inverted for BCLAF1. Weakening of the splice signal by the alternative variant increases predicted binding probabilities for QKI and HNRNPL.

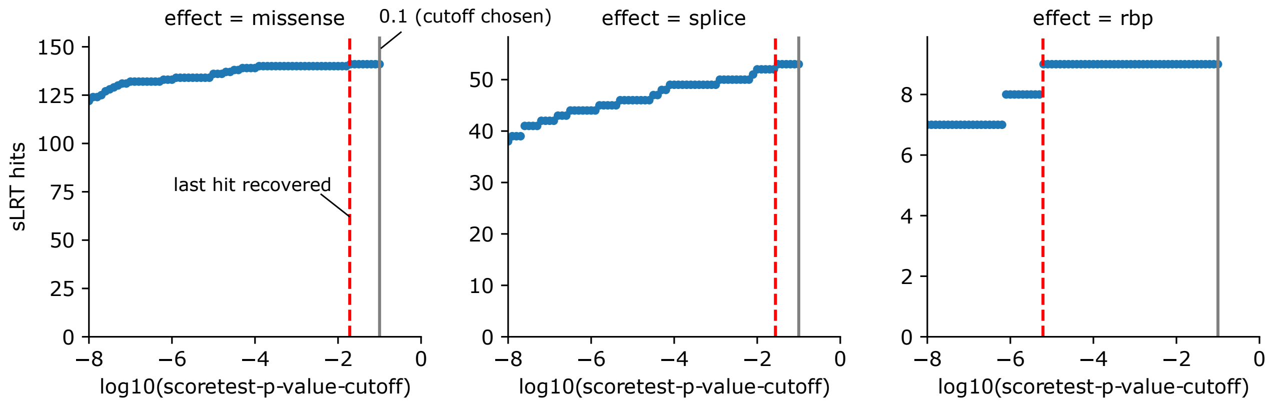

Supplementary Figure 6: **sLRT number of significant gene-biomarker associations vs. score test cutoff.** We consider the scenario of performing five score tests per gene and biomarker (one collapsing and one kernel-based test for missense and splice variants, and one kernel-based test for RBP-variants), resulting in 2,540,128 tests genome-wide and a Bonferroni-corrected p-value threshold of 1.96×10^{-8} (FWER ≤ 0.05). The plots show the number of significant associations found by the sLRT depending on the nominal significance cutoff chosen for the score tests (which determine whether the LRT is performed). We used the cutoff of 0.1 (gray vertical line) in our analysis.

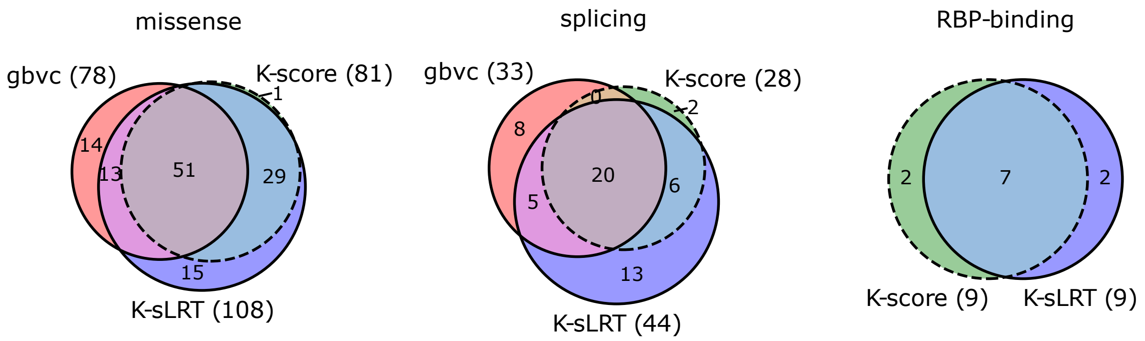

Supplementary Figure 7: **Kernel-based sLRT vs. score test comparison.** We consider the scenario of performing five score tests per gene and biomarker (one collapsing and one kernel-based test for missense and splice variants, and one kernel-based test for RBP-variants), resulting in 2,540,128 tests genome-wide and a Bonferroni-corrected p-value threshold of 1.96×10^{-8} (FWER ≤ 0.05). Venn diagrams showing the significant locus-biomarker associations identified by the kernel-based score test (K-score), kernel-based sLRT (K-sLRT) and gene-based variant collapsing (gbvc). For missense and splice variants, the kernel-based sLRT identified more significant associations than the kernel-based score test. A large fraction of these additional associations was also found by gbvc tests.

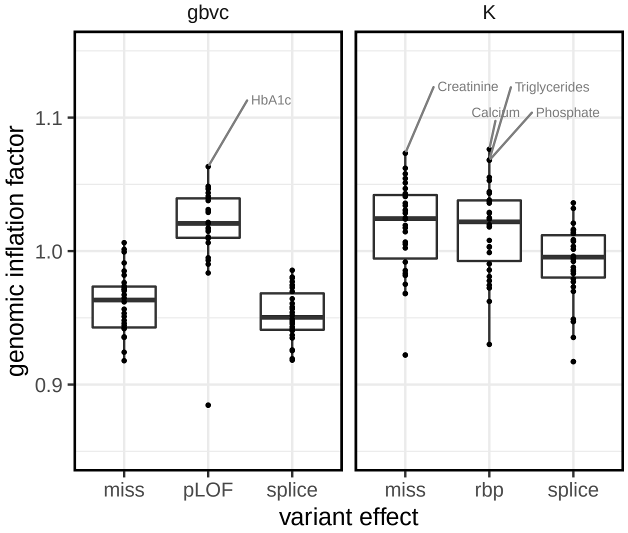

Supplementary Figure 8: **Genomic inflation factor across models.** We calculated λ_{GC} for all tests that were performed exome-wide in the all-ancestry analysis. Boxplots showing λ_{GC} across all phenotypes (y-axis, $n = 30$ phenotypes) against the different variant categories and types of association tests. All values refer to the sLRT, except for gbvc-pLOF, where we only performed the score test. Left: gene based variant collapsing (gbvc); Right: kernel-based tests (K). QQ-plots for all models that resulted in at least one significant association are given in Supplementary Data 5. Center lines denote the medians. The lower and upper hinges indicate 25th and 75th percentiles. Whiskers extend to the largest/lowest values no further than 1.5 $\times$ IQR away from the upper/lower hinges and black points denote outliers. λ_{GC} ranged from 0.84 (pLOF, gbvc, phenotype: Rheumatoid factor) to 1.076 (rbp, kernel-based test, phenotype: Calcium)

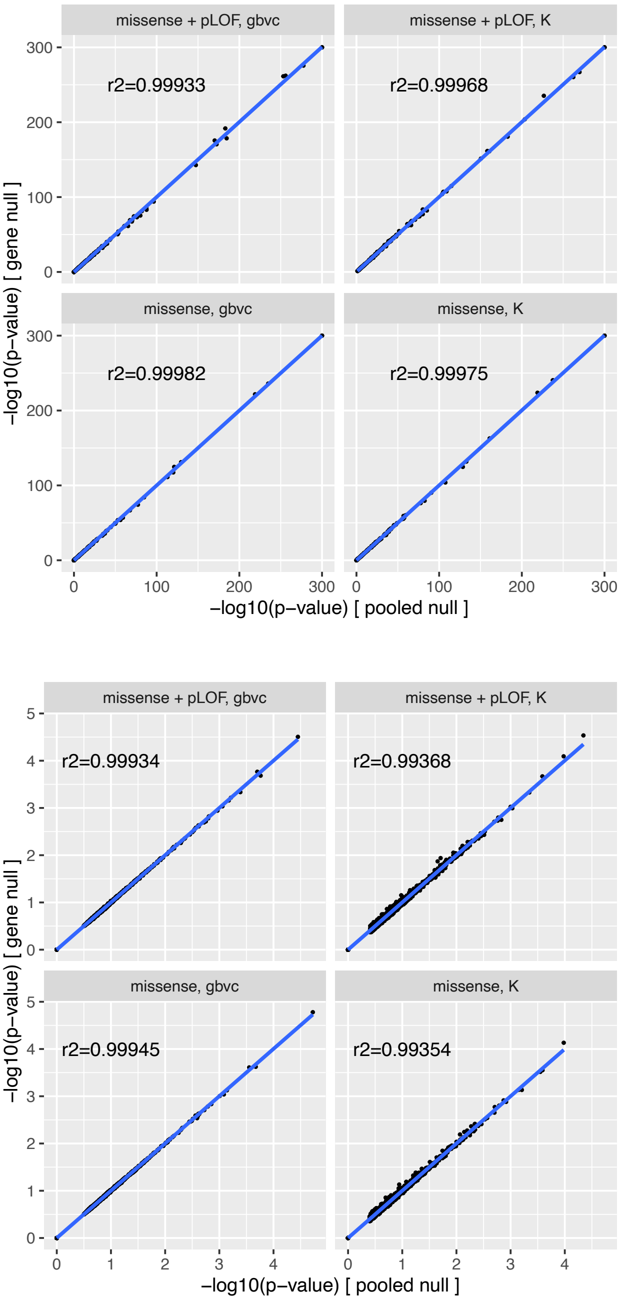

Supplementary Figure 9: **RLRT p-value comparison (gene-specific vs. pooled null distribution) for tests with missense variants.** Scatter plots showing the negative log10-p-values produced by pooled null distributions vs. those from gene-specific null distributions for associations close to or below the study-wide significance threshold (FWER $\leq$ 0.05) (top) and randomly selected genes (bottom). Blue lines indicate the linear regression fit. *r2*: r-squared values calculated for the points that do not lie in the origin.

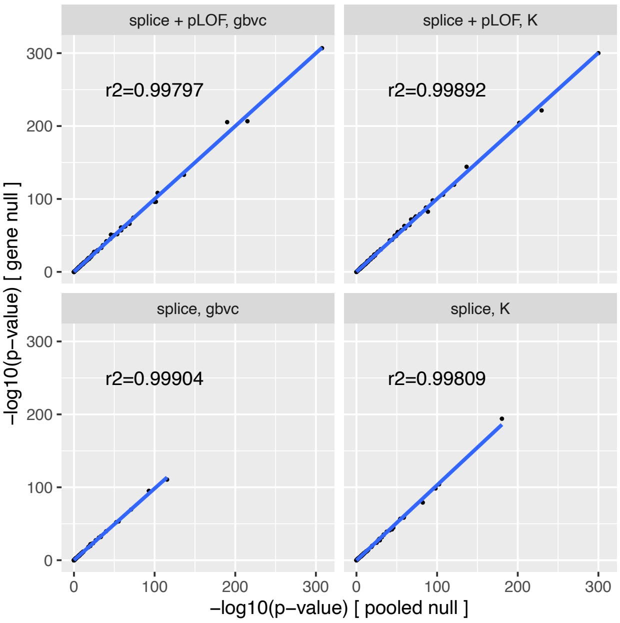

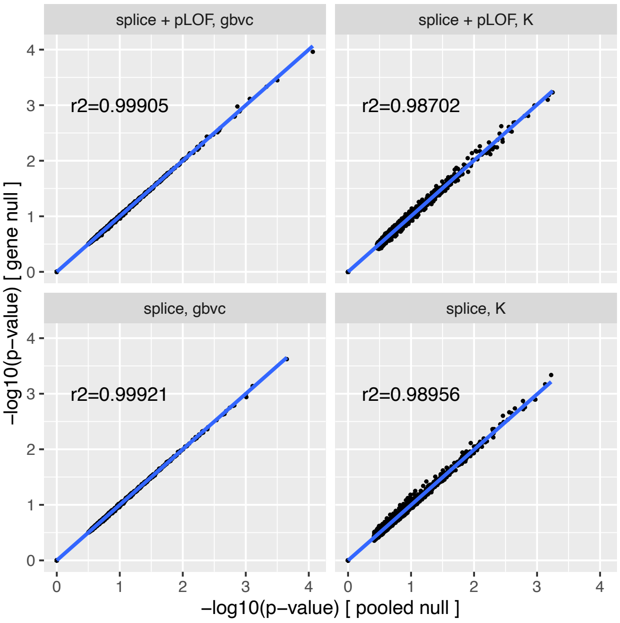

Supplementary Figure 10: **RLRT p-value comparison (gene-specific vs. pooled null distribution) for tests with splice variants.** Scatter plots showing the negative log10-p-values produced by pooled null distributions vs. those from gene-specific null distributions for associations close to or below the study-wide significance threshold (FWER $\leq$ 0.05) (top) and randomly selected genes (bottom). Blue lines indicate the linear regression fit. *r2*: r-squared values calculated for the points that do not lie in the origin.

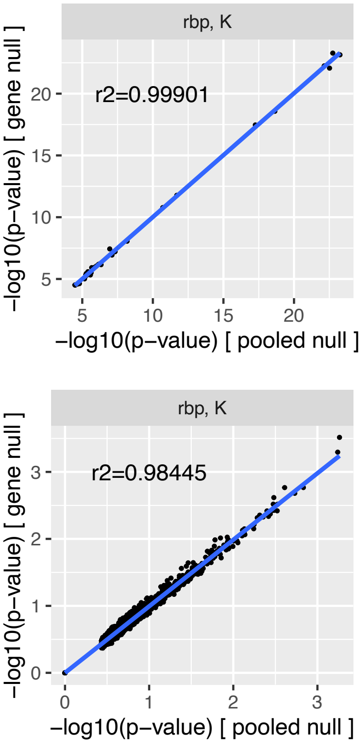

Supplementary Figure 11: **RLRT p-value comparison (gene-specific vs. pooled null distribution) for tests with predicted RBP-binding altering variants.** Scatter plots showing the negative log10-p-values produced by pooled null distributions vs. those from gene-specific null distributions for associations close to or below the study-wide significance threshold (FWER ≤ 0.05) (top) and randomly selected genes (bottom). Blue lines indicate the linear regression fit. *r2*: r-squared values calculated for the points that do not lie in the origin.

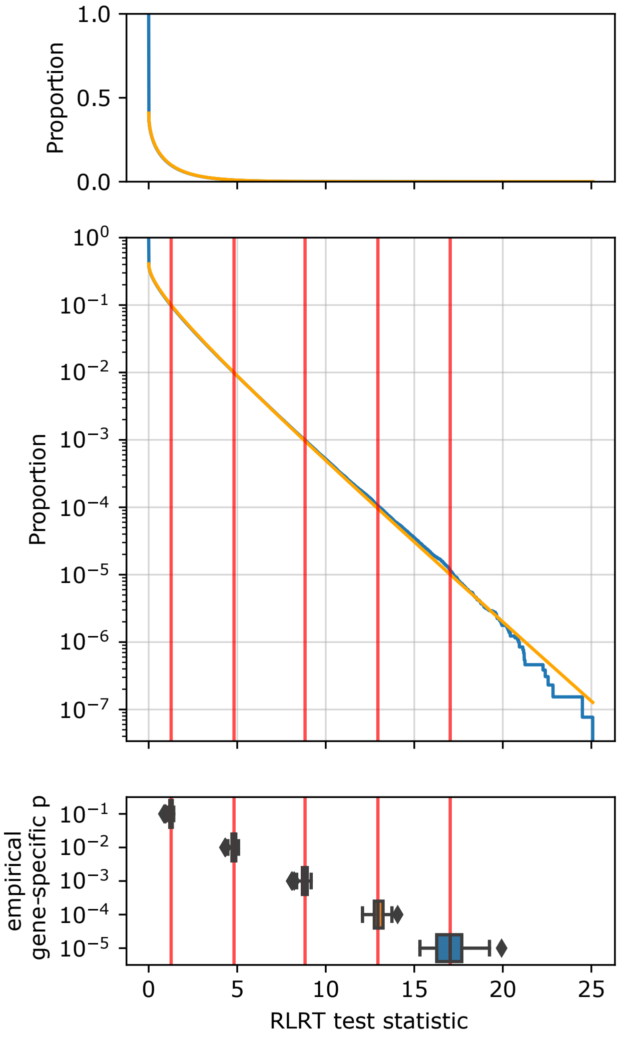

Supplementary Figure 12: **RLRT pooled null distribution vs empirical gene-specific quantiles example.** For the kernel-based RLRT with missense variants and phenotype triglycerides, we sampled 250,000 test statistics each for 52 genes and fit a χ^2-mixture distribution (parametric null) to the pooled test statistics ($0.59 \times \chi^2_0 + (1 - 0.59) \times 0.96 \times \chi^2_{0.98}$). The top two panels show the empirical inverse cumulative distribution function of the pooled test statistics (y; blue line), against the value of the test statistic (x). The survival function of the non-zero mixture component of the parametric null is overlaid in orange. In the lower panel, the box plot shows the gene-specific quantiles of test statistics (x) corresponding to gene-specific emirical p-value thresholds of 10^{-1}, 10^{-2}, 10^{-3}, 10^{-4} and 10^{-5} (y) for the 52 genes ($n = 52$). Center lines denote the medians, and are extended to the plot above in red, showing that the mixture distribution accurately captures the median gene-specific quantiles in the tail. The lower and upper hinges indicate 25th and 75th percentiles. Whiskers extend to the largest/lowest values no further than 1.5 × IQR away from the upper/lower hinges and black points denote outliers. Empirical cutoffs ranged from 0.85 to 1.41 for $p = 10^{-1}$, 4.3 to 5.07 for $p = 10^{-2}$, 8.07 to 9.18 for $p = 10^{-3}$, 12.06 to 14.06 for $p = 10^{-4}$, and 15.32 to 19.93 for $p = 10^{-5}$.

Supplementary References.

[1] Ghanbari, M. & Ohler, U. Deep neural networks for interpreting rna-binding protein target preferences. *Genome research* **30**, 214–226 (2020).
