# Supplementary material for: Identifying interpretable gene-biomarker associations with functionally informed kernel-based tests in 190,000 exomes
Source: Nat Commun. 2022 Sep 10;13:5332. doi: 10.1038/s41467-022-32864-2 (PMC9464252; doi:10.1038/s41467-022-32864-2)
Supplement: Supplementary file 3 — Description of Additional Supplementary Files [file 41467_2022_32864_MOESM3_ESM.pdf]

Description of Additional Supplementary Files

File Name: Supplementary Data 1
Description: Tables containing all genome-wide significant gene-biomarker associations (FWER ≤ 0.05) across different models, including their conditional p-values, test-type and implementation used, the parametric null distributions for the restricted likelihood ratio test ("RLRT null distributions" worksheet), gene locations, and variants that were conditioned on. Column descriptions are provided in the "Legend" worksheet.

File Name: Supplementary Data 2
Description: Variables (genotypes, phenotypes, covariates) used in the analysis, together with their UK Biobank data field identifiers and EFO ontology numbers used to query GWAS databases.

File Name: Supplementary Data 3
Description: Table accompanying Figure 3. Significant associations identified in the all-ancestry analysis (AA) but not in the analysis limited to individuals with inferred European ancestry (EUR) or vice versa. The reported p-values are the smallest across the different models for the respective genes. Column descriptions are provided in the "Legend" worksheet.

File Name: Supplementary Data 4
Description: Comparison to other UK Biobank association studies. List of genes identified by biomarker associations in our analysis together with their associations identified in Sinnott-Armstrong 2021 et al., Wang et al. 2021 or Backman et al. 2021. Column descriptions are provided in the "Legend" worksheet.

File Name: Supplementary Data 5
Description: QQ-Plots. Zip-compressed archive containing PDF files with quantile-quantile plots for all biomarkers and models that had at least one association below the significance threshold in the all-ancestry analysis, for tests that were performed genome-wide. P-values are truncated to 1e-30.
