# Supplementary figures and images for: Identifying interpretable gene-biomarker associations with functionally informed kernel-based tests in 190,000 exomes (part 2 of 2)
Source: Nat Commun. 2022 Sep 10;13:5332. doi: 10.1038/s41467-022-32864-2 (PMC9464252; doi:10.1038/s41467-022-32864-2)

Alanine\_aminotransferase, test type: gbvc,  
implementation: sLRT, var. effect: splice

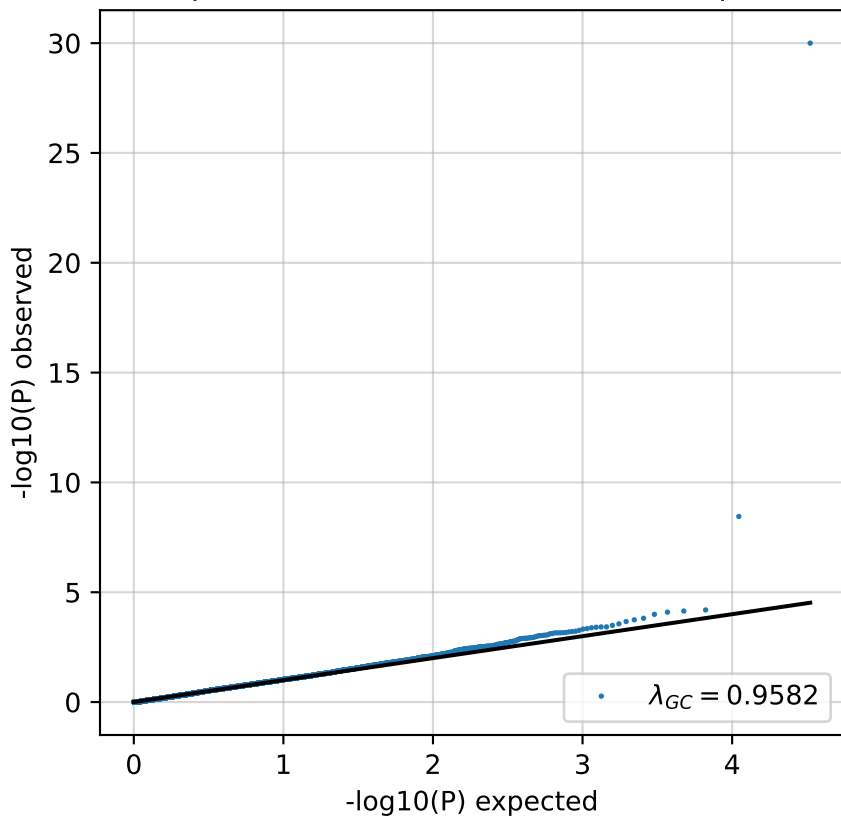

Supplement: Supplementary file 8 — Supplementary Data 5 [file 41467_2022_32864_MOESM8_ESM.zip › qqplots/splice_gbvc_sLRT_Alanine_aminotransferase.pdf]

Albumin, test type: gbvc,  
implementation: sLRT, var. effect: splice

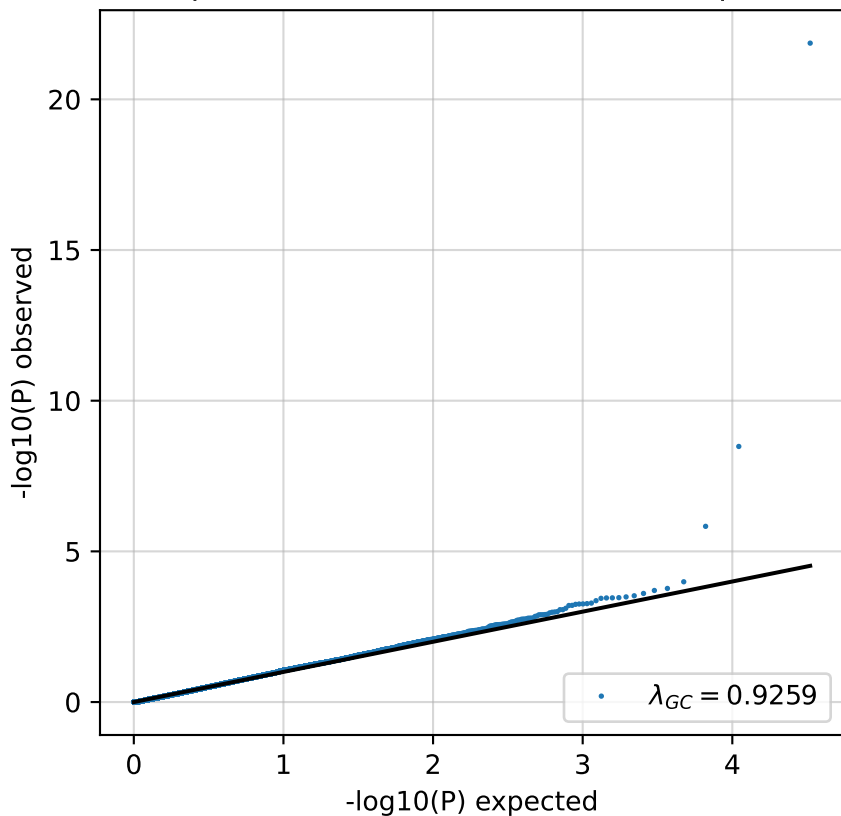

Supplement: Supplementary file 8 — Supplementary Data 5 [file 41467_2022_32864_MOESM8_ESM.zip › qqplots/splice_gbvc_sLRT_Albumin.pdf]

Alkaline\_phosphatase, test type: gbvc,  
implementation: sLRT, var. effect: splice

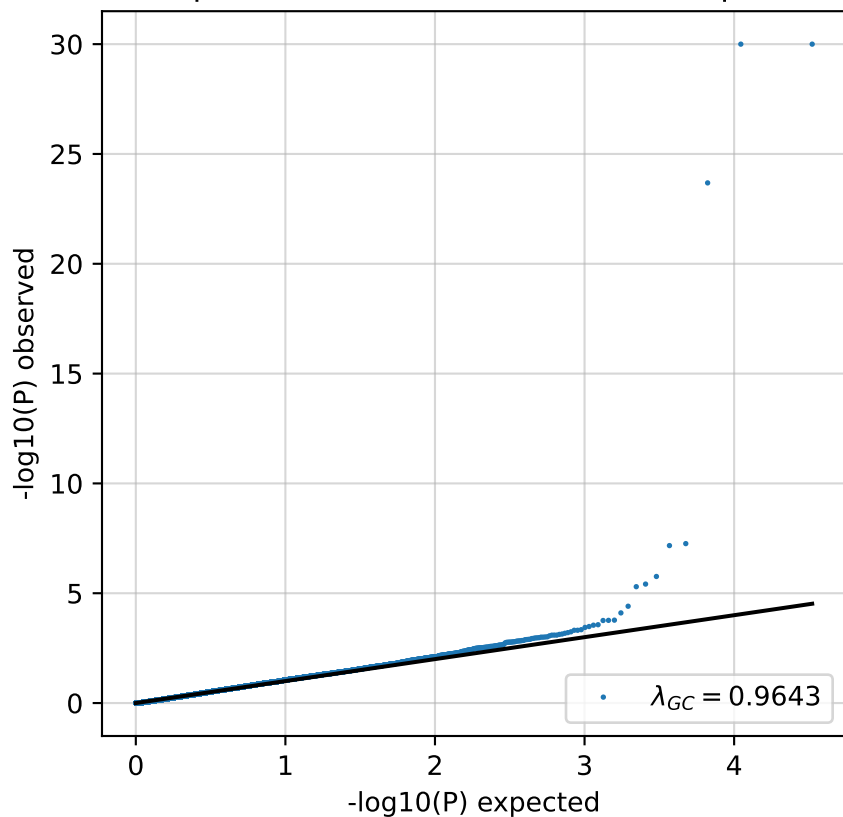

Supplement: Supplementary file 8 — Supplementary Data 5 [file 41467_2022_32864_MOESM8_ESM.zip › qqplots/splice_gbvc_sLRT_Alkaline_phosphatase.pdf]

Apolipoprotein\_A, test type: gbvc,  
implementation: sLRT, var. effect: splice

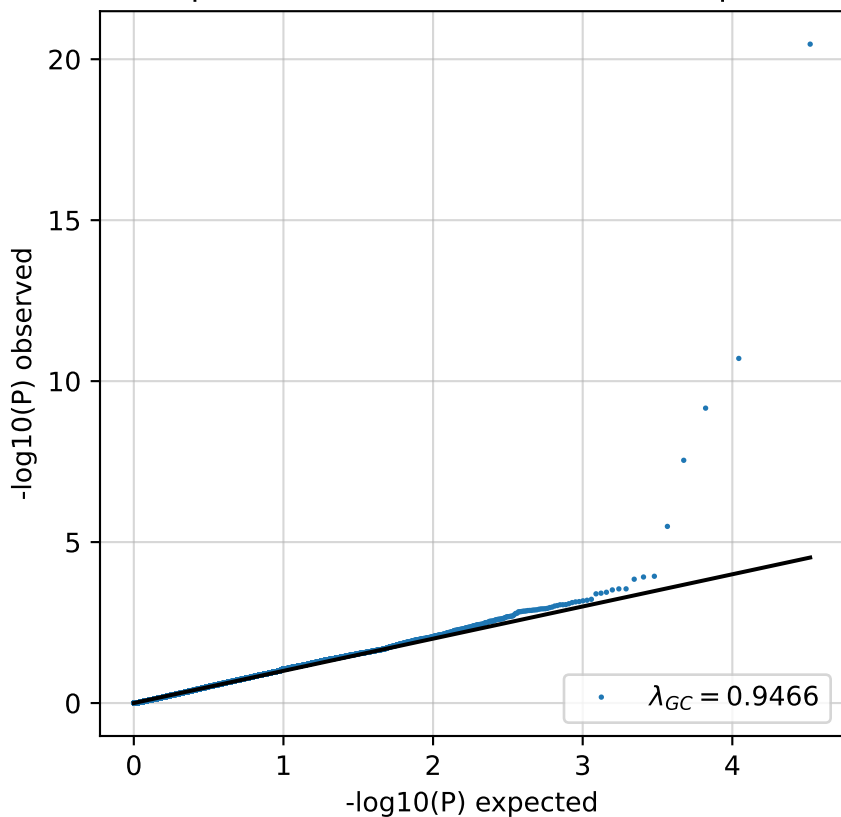

Supplement: Supplementary file 8 — Supplementary Data 5 [file 41467_2022_32864_MOESM8_ESM.zip › qqplots/splice_gbvc_sLRT_Apolipoprotein_A.pdf]

Apolipoprotein\_B, test type: gbvc,  
implementation: sLRT, var. effect: splice

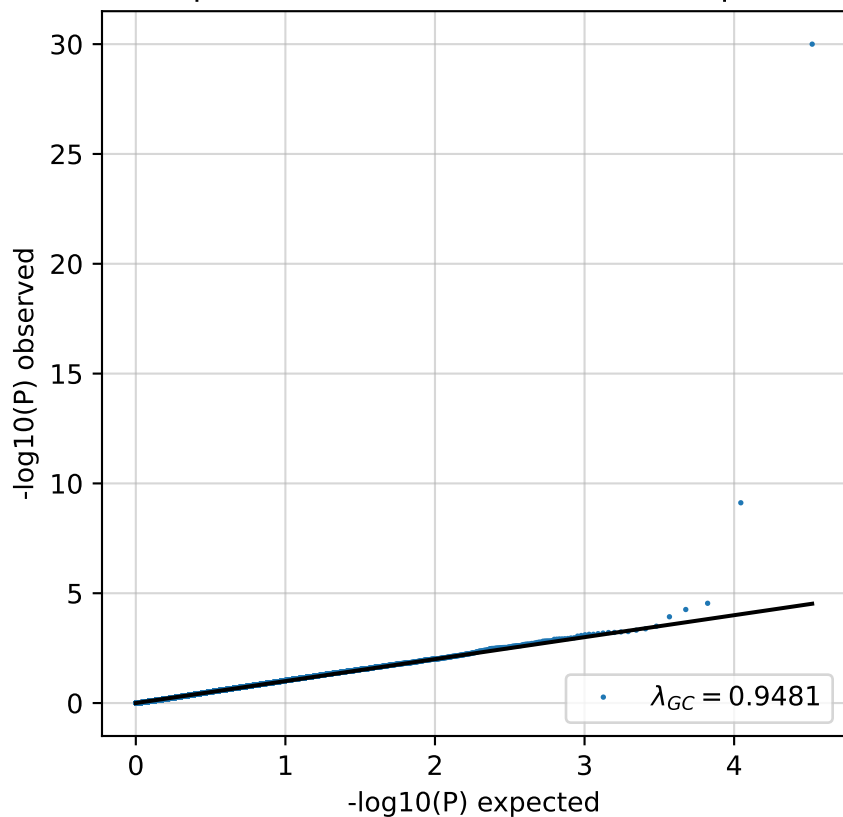

Supplement: Supplementary file 8 — Supplementary Data 5 [file 41467_2022_32864_MOESM8_ESM.zip › qqplots/splice_gbvc_sLRT_Apolipoprotein_B.pdf]

Aspartate\_aminotransferase, test type: gbvc,  
implementation: sLRT, var. effect: splice

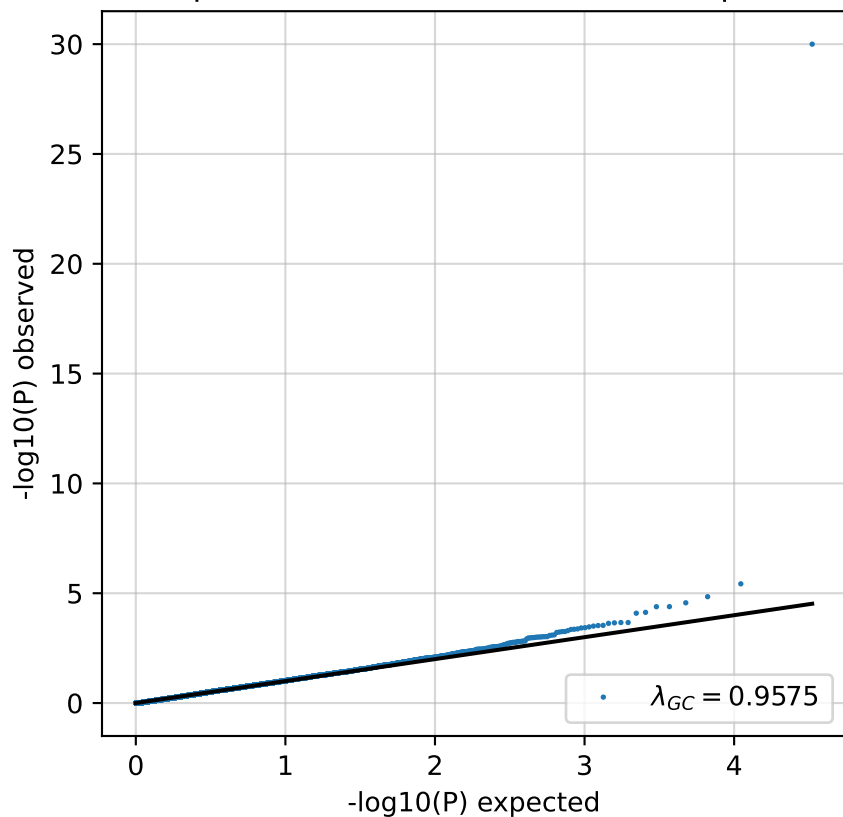

Supplement: Supplementary file 8 — Supplementary Data 5 [file 41467_2022_32864_MOESM8_ESM.zip › qqplots/splice_gbvc_sLRT_Aspartate_aminotransferase.pdf]

Calcium, test type: gbvc,  
implementation: sLRT, var. effect: splice

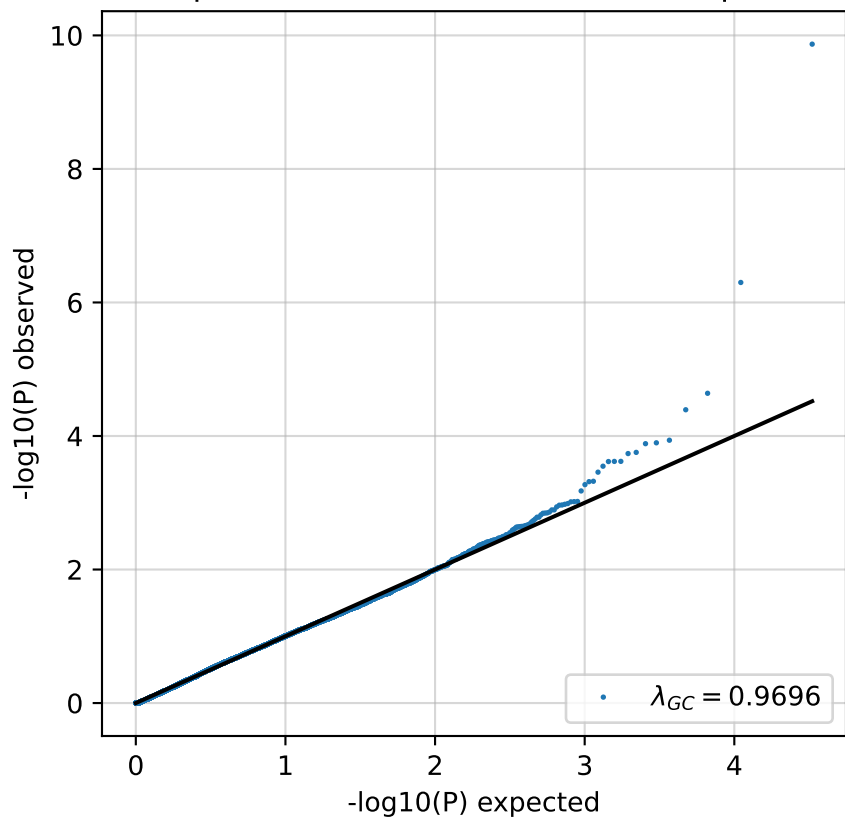

Supplement: Supplementary file 8 — Supplementary Data 5 [file 41467_2022_32864_MOESM8_ESM.zip › qqplots/splice_gbvc_sLRT_Calcium.pdf]

Cholesterol, test type: gbvc,  
implementation: sLRT, var. effect: splice

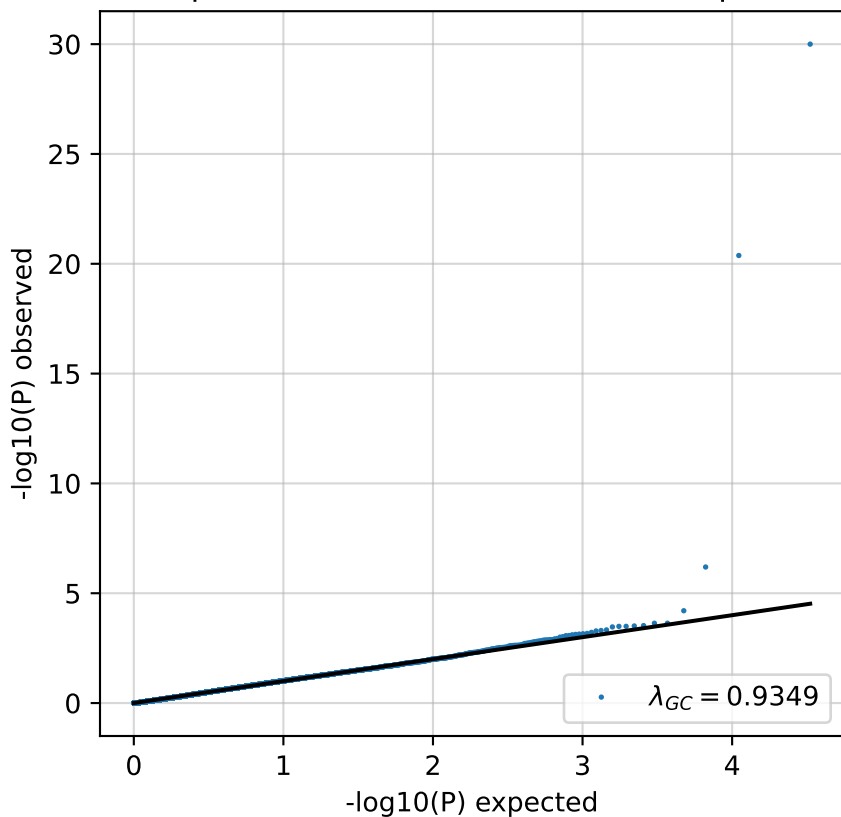

Supplement: Supplementary file 8 — Supplementary Data 5 [file 41467_2022_32864_MOESM8_ESM.zip › qqplots/splice_gbvc_sLRT_Cholesterol.pdf]

Creatinine, test type: gbvc,  
implementation: sLRT, var. effect: splice

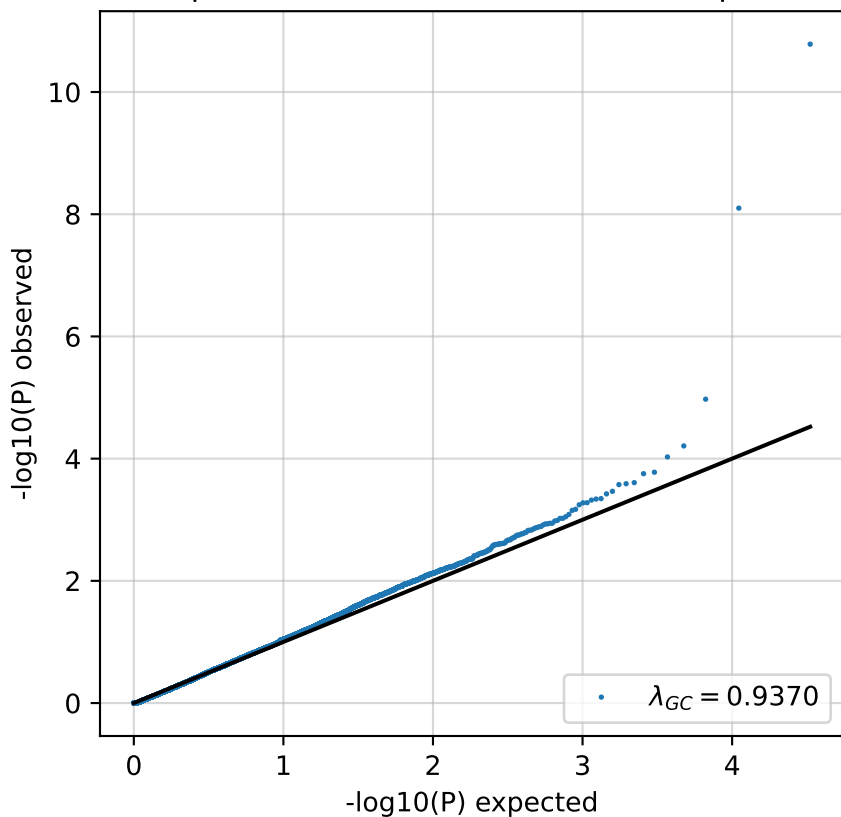

Supplement: Supplementary file 8 — Supplementary Data 5 [file 41467_2022_32864_MOESM8_ESM.zip › qqplots/splice_gbvc_sLRT_Creatinine.pdf]

Cystatin\_C, test type: gbvc,  
implementation: sLRT, var. effect: splice

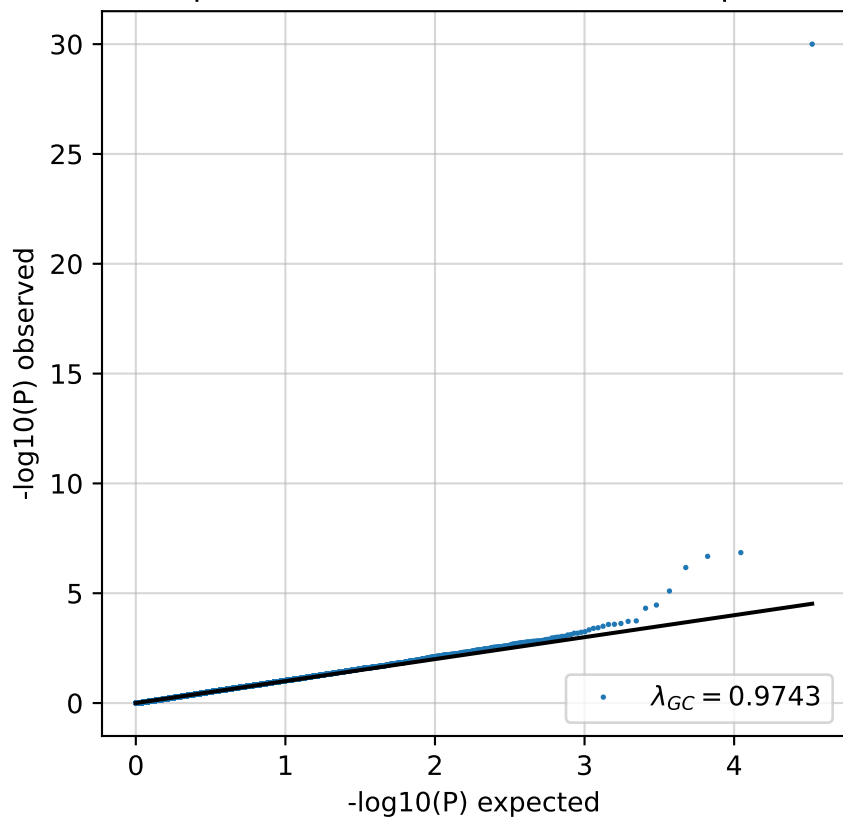

Supplement: Supplementary file 8 — Supplementary Data 5 [file 41467_2022_32864_MOESM8_ESM.zip › qqplots/splice_gbvc_sLRT_Cystatin_C.pdf]

Direct\_bilirubin, test type: gbvc,  
implementation: sLRT, var. effect: splice

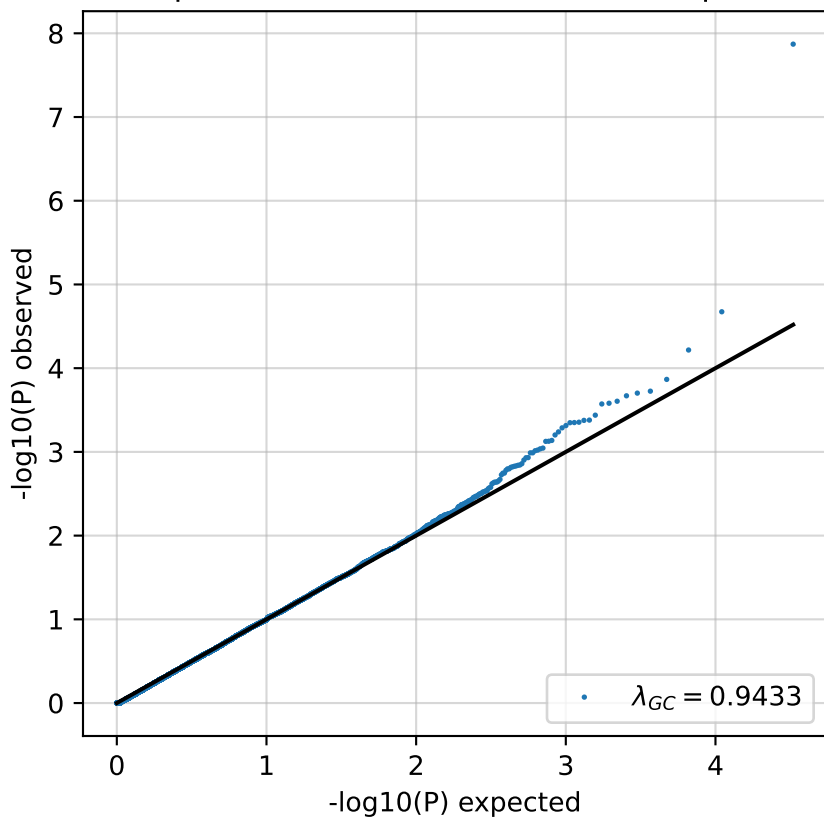

Supplement: Supplementary file 8 — Supplementary Data 5 [file 41467_2022_32864_MOESM8_ESM.zip › qqplots/splice_gbvc_sLRT_Direct_bilirubin.pdf]

Gamma\_glutamyltransferase, test type: gbvc,  
implementation: sLRT, var. effect: splice

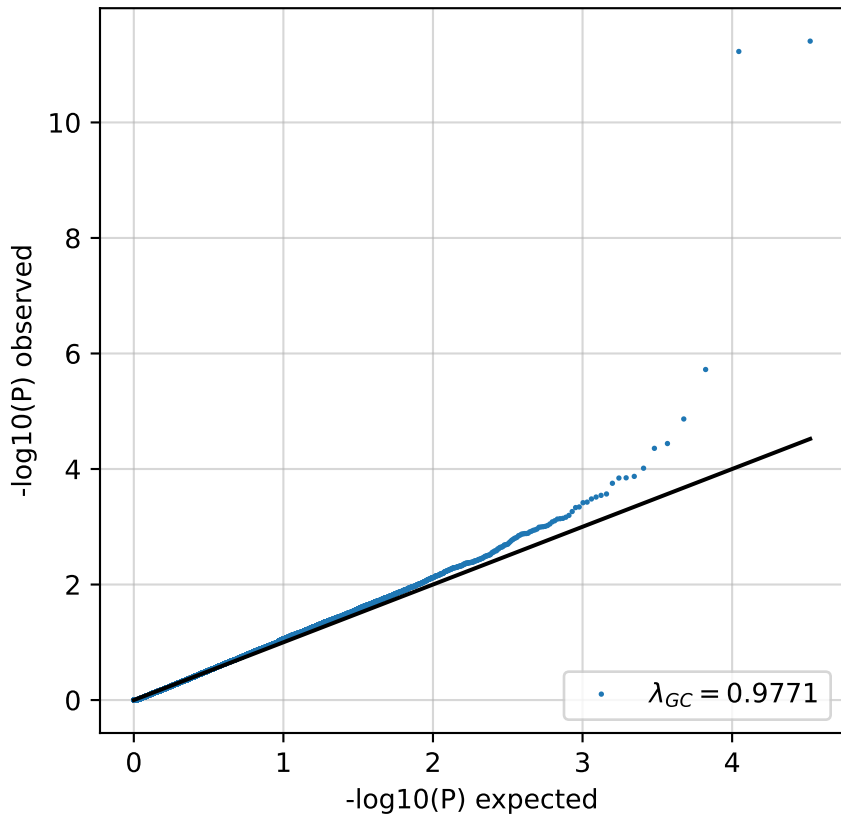

Supplement: Supplementary file 8 — Supplementary Data 5 [file 41467_2022_32864_MOESM8_ESM.zip › qqplots/splice_gbvc_sLRT_Gamma_glutamyltransferase.pdf]

Glycated\_haemoglobin\_(HbA1c), test type: gbvc,  
implementation: sLRT, var. effect: splice

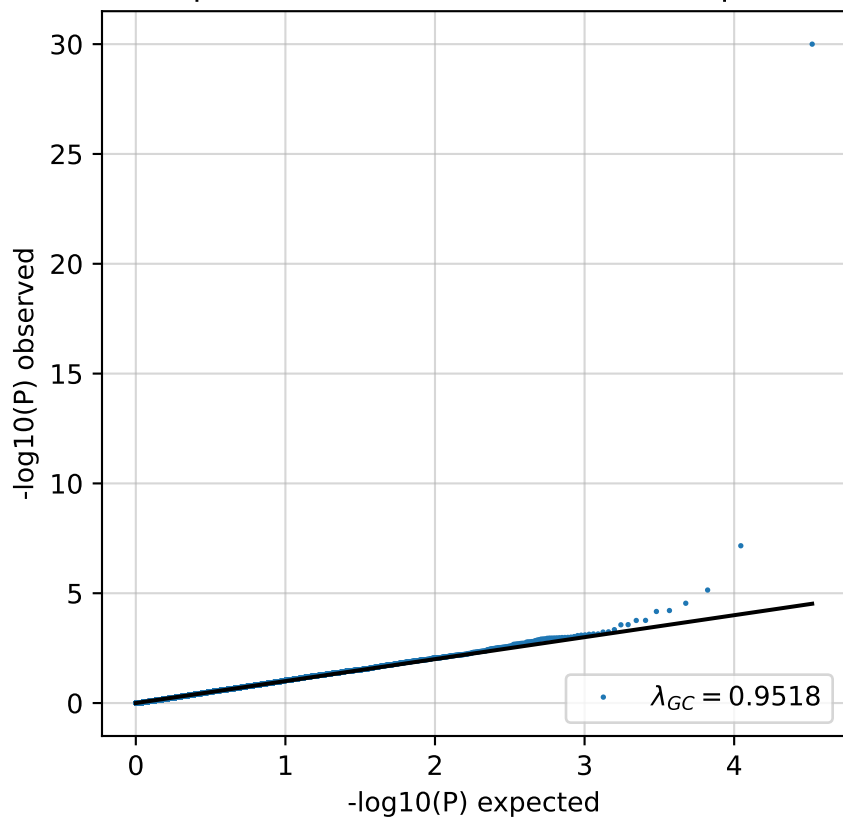

Supplement: Supplementary file 8 — Supplementary Data 5 [file 41467_2022_32864_MOESM8_ESM.zip › qqplots/splice_gbvc_sLRT_Glycated_haemoglobin_HbA1c.pdf]

HDL\_cholesterol, test type: gbvc,  
implementation: sLRT, var. effect: splice

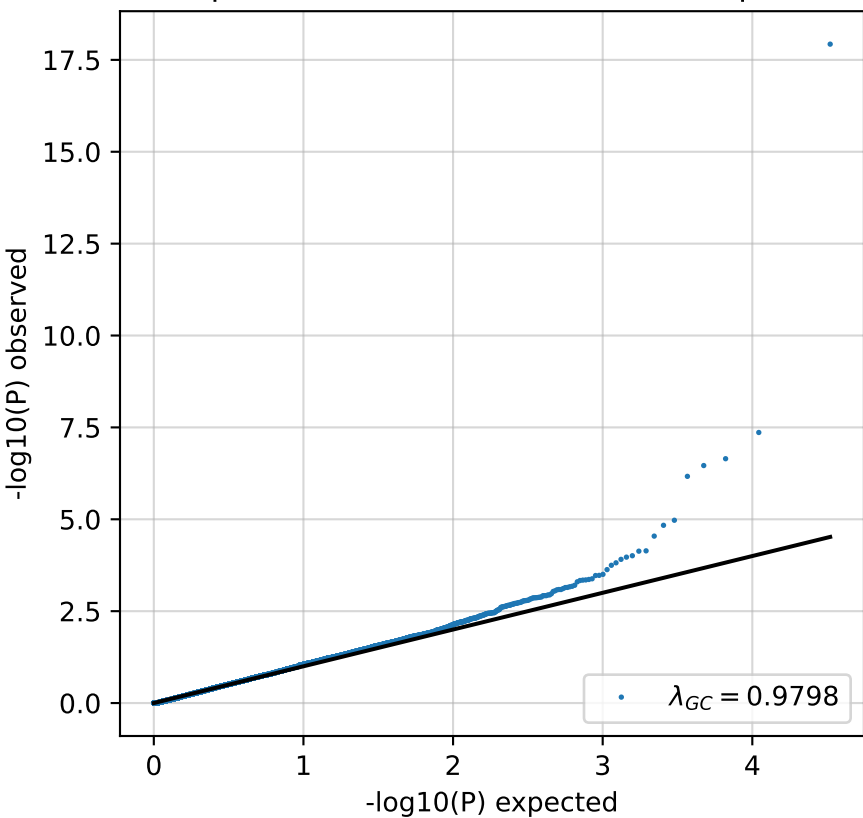

Supplement: Supplementary file 8 — Supplementary Data 5 [file 41467_2022_32864_MOESM8_ESM.zip › qqplots/splice_gbvc_sLRT_HDL_cholesterol.pdf]

IGF-1, test type: gbvc,  
implementation: sLRT, var. effect: splice

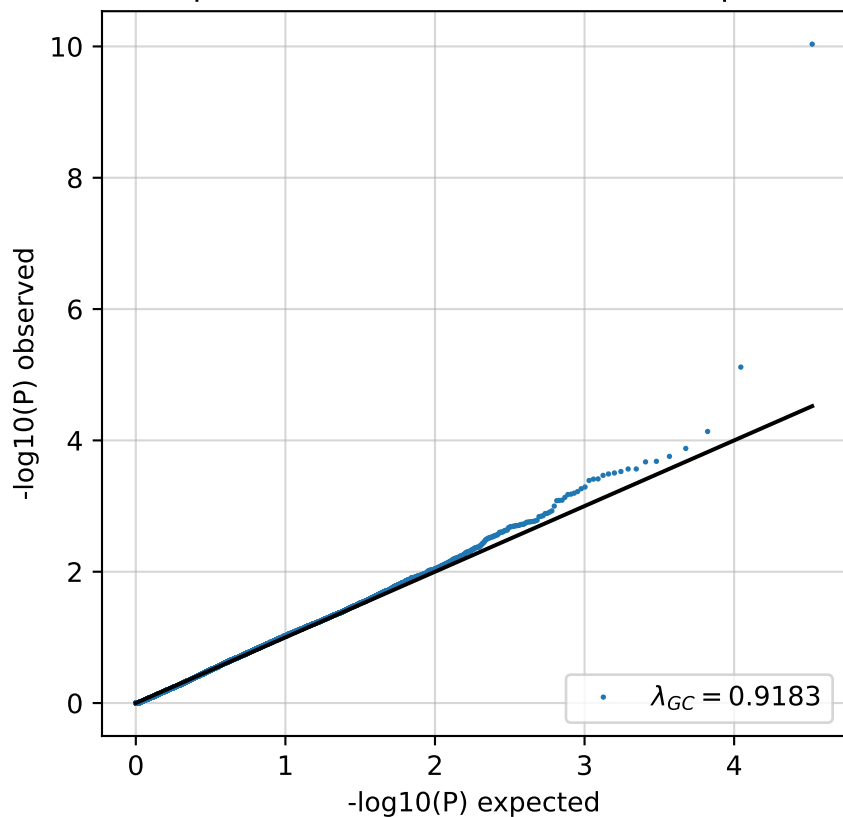

Supplement: Supplementary file 8 — Supplementary Data 5 [file 41467_2022_32864_MOESM8_ESM.zip › qqplots/splice_gbvc_sLRT_IGF1.pdf]

LDL\_direct, test type: gbvc,  
implementation: sLRT, var. effect: splice

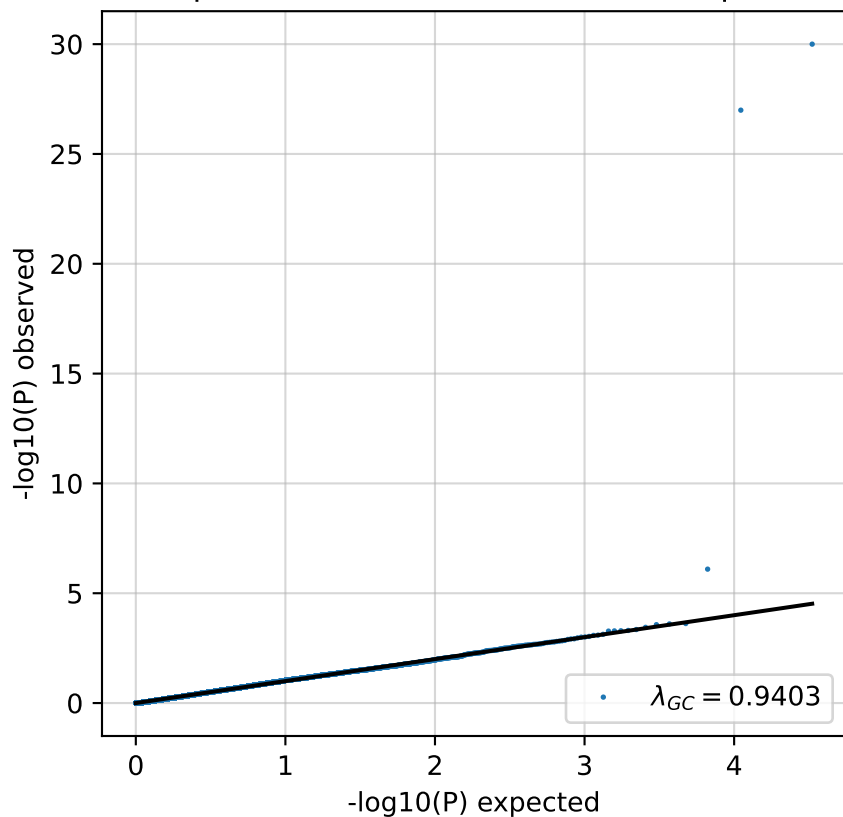

Supplement: Supplementary file 8 — Supplementary Data 5 [file 41467_2022_32864_MOESM8_ESM.zip › qqplots/splice_gbvc_sLRT_LDL_direct.pdf]

Lipoprotein\_A, test type: gbvc,  
implementation: sLRT, var. effect: splice

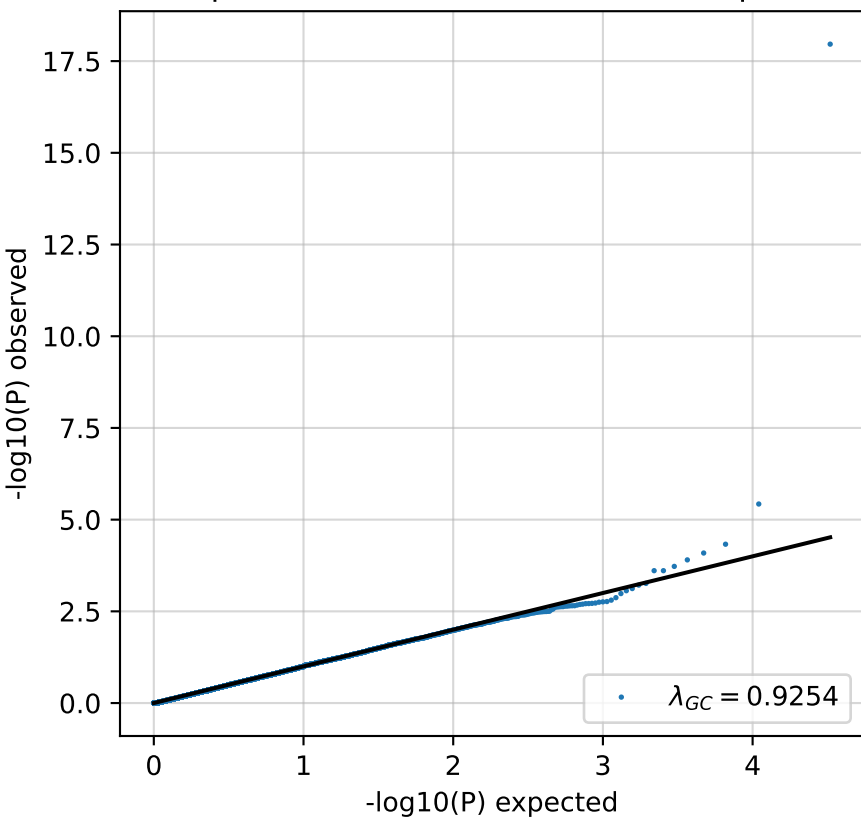

Supplement: Supplementary file 8 — Supplementary Data 5 [file 41467_2022_32864_MOESM8_ESM.zip › qqplots/splice_gbvc_sLRT_Lipoprotein_A.pdf]

Total\_bilirubin, test type: gbvc,  
implementation: sLRT, var. effect: splice

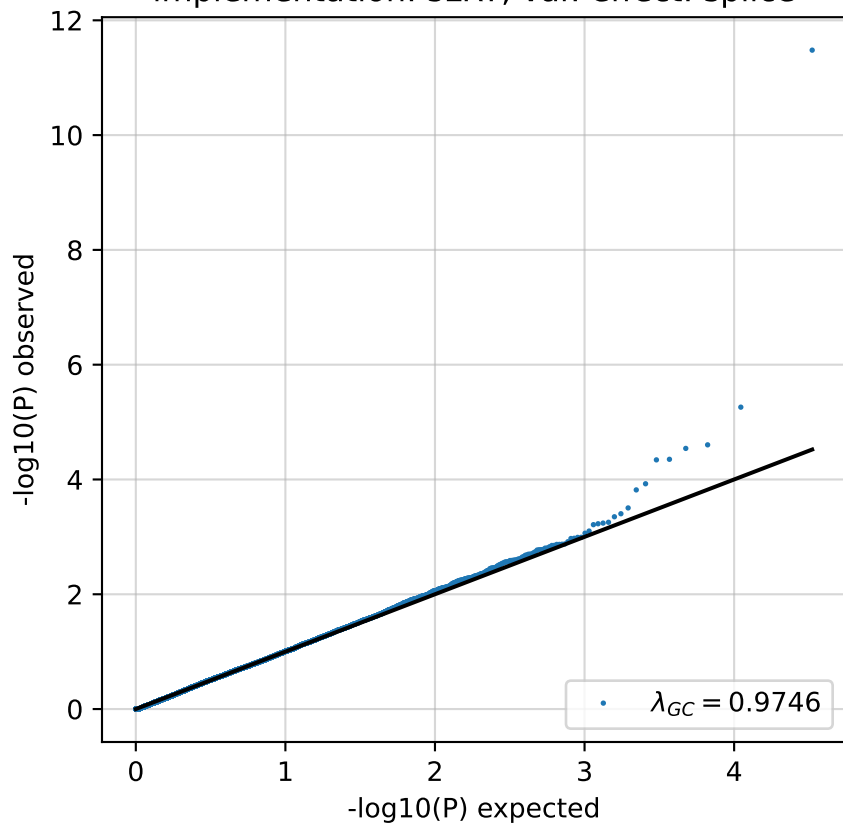

Supplement: Supplementary file 8 — Supplementary Data 5 [file 41467_2022_32864_MOESM8_ESM.zip › qqplots/splice_gbvc_sLRT_Total_bilirubin.pdf]

Triglycerides, test type: gbvc,  
implementation: sLRT, var. effect: splice

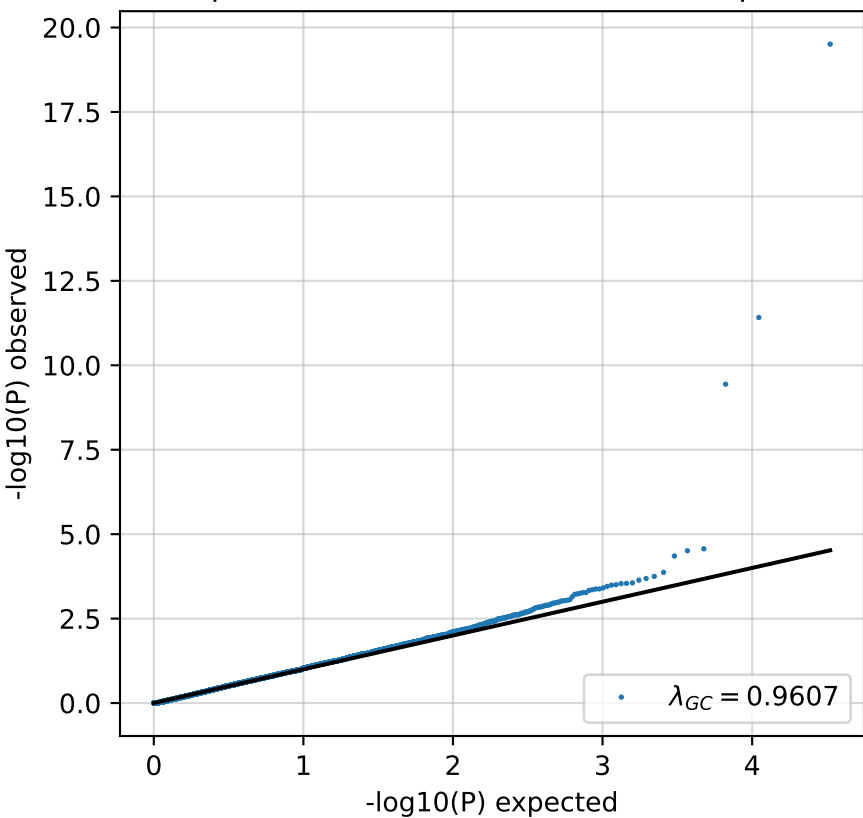

Supplement: Supplementary file 8 — Supplementary Data 5 [file 41467_2022_32864_MOESM8_ESM.zip › qqplots/splice_gbvc_sLRT_Triglycerides.pdf]

Urate, test type: gbvc,  
implementation: sLRT, var. effect: splice

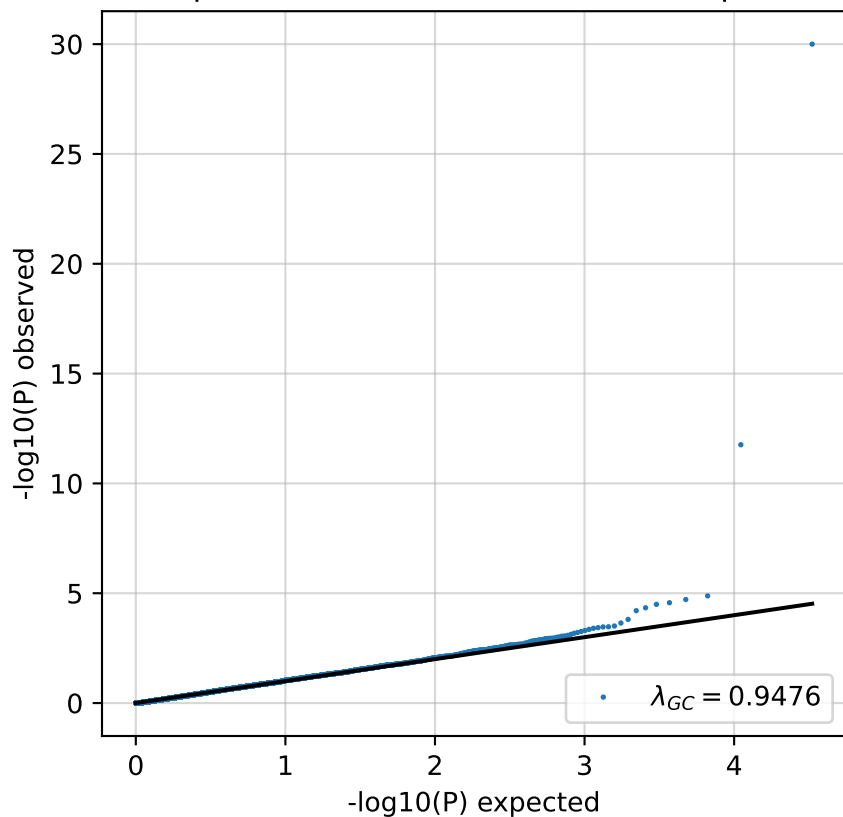

Supplement: Supplementary file 8 — Supplementary Data 5 [file 41467_2022_32864_MOESM8_ESM.zip › qqplots/splice_gbvc_sLRT_Urate.pdf]
