# Supplementary figures and images for: Identifying interpretable gene-biomarker associations with functionally informed kernel-based tests in 190,000 exomes (part 1 of 2)
Source: Nat Commun. 2022 Sep 10;13:5332. doi: 10.1038/s41467-022-32864-2 (PMC9464252; doi:10.1038/s41467-022-32864-2)

Alanine\_aminotransferase, test type: K,  
implementation: sLRT, var. effect: miss

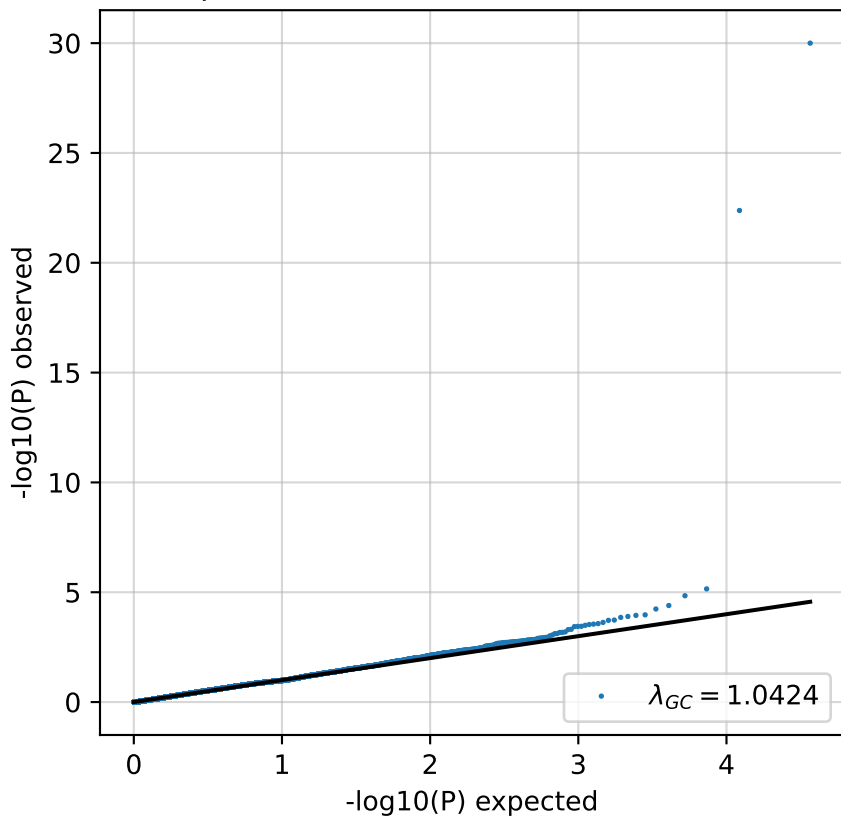

Supplement: Supplementary file 8 — Supplementary Data 5 [file 41467_2022_32864_MOESM8_ESM.zip › qqplots/miss_K_sLRT_Alanine_aminotransferase.pdf]

Albumin, test type: K,  
implementation: sLRT, var. effect: miss

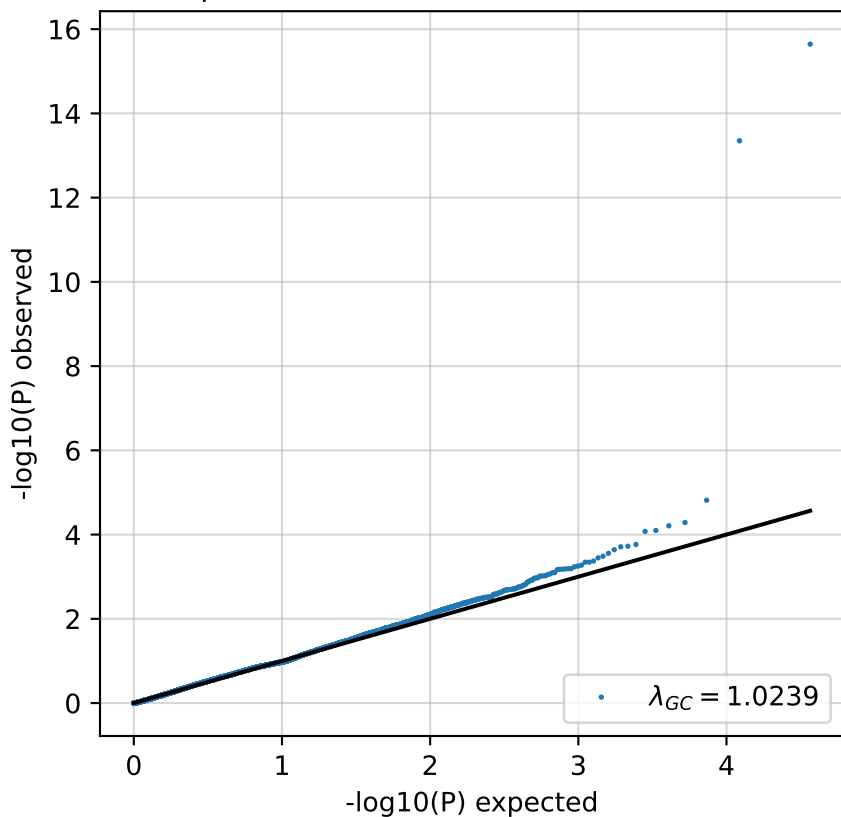

Supplement: Supplementary file 8 — Supplementary Data 5 [file 41467_2022_32864_MOESM8_ESM.zip › qqplots/miss_K_sLRT_Albumin.pdf]

Alkaline\_phosphatase, test type: K,  
implementation: sLRT, var. effect: miss

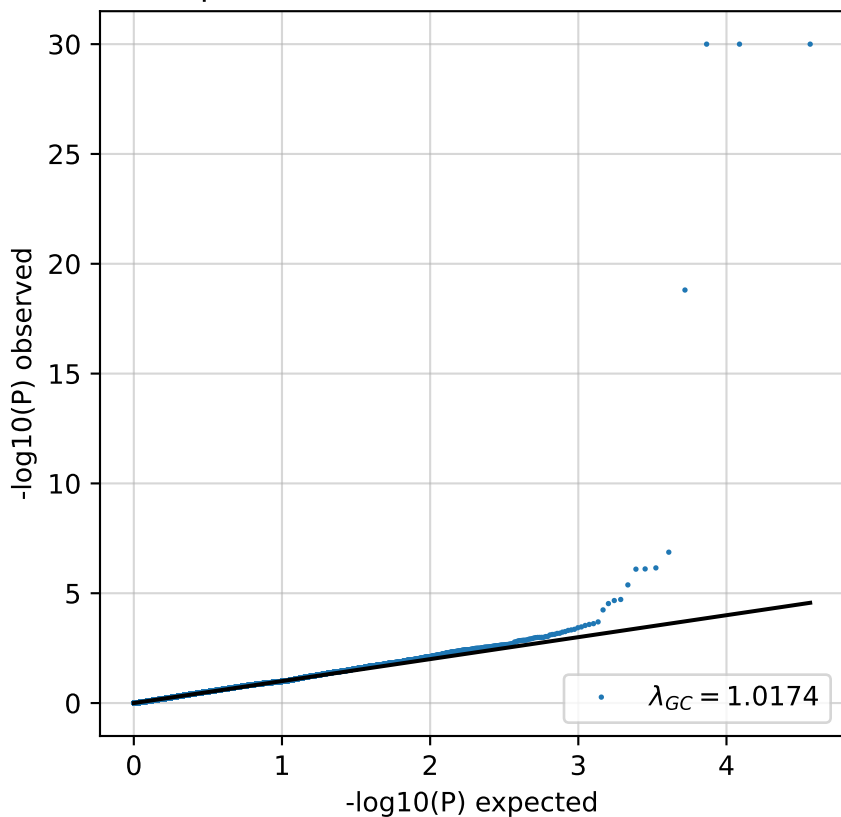

Supplement: Supplementary file 8 — Supplementary Data 5 [file 41467_2022_32864_MOESM8_ESM.zip › qqplots/miss_K_sLRT_Alkaline_phosphatase.pdf]

Apolipoprotein\_A, test type: K,  
implementation: sLRT, var. effect: miss

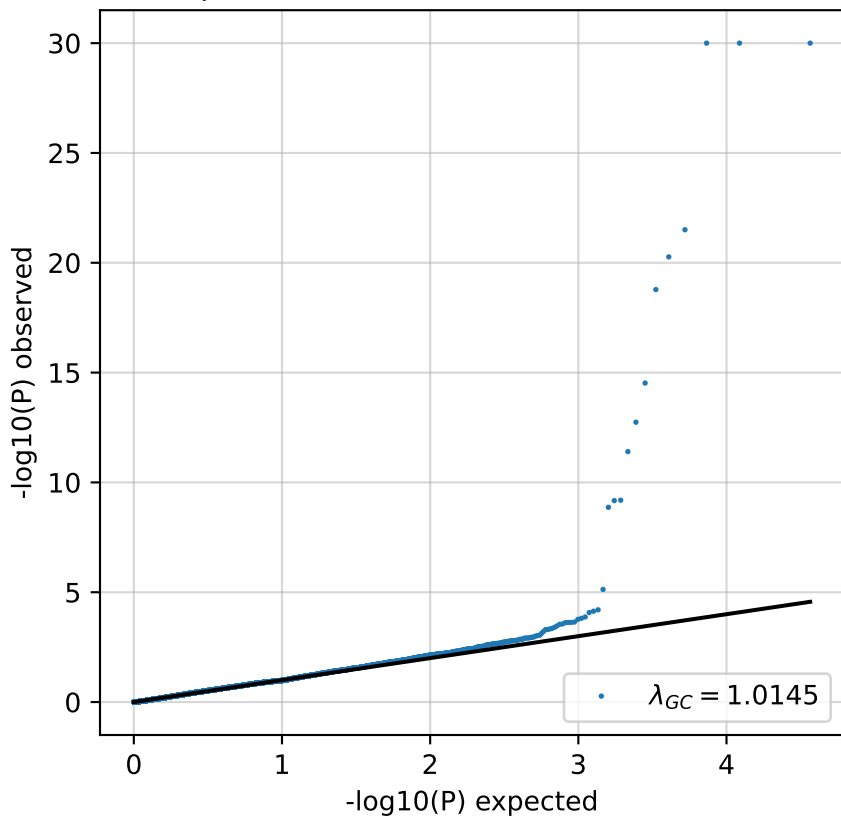

Supplement: Supplementary file 8 — Supplementary Data 5 [file 41467_2022_32864_MOESM8_ESM.zip › qqplots/miss_K_sLRT_Apolipoprotein_A.pdf]

Apolipoprotein\_B, test type: K,  
implementation: sLRT, var. effect: miss

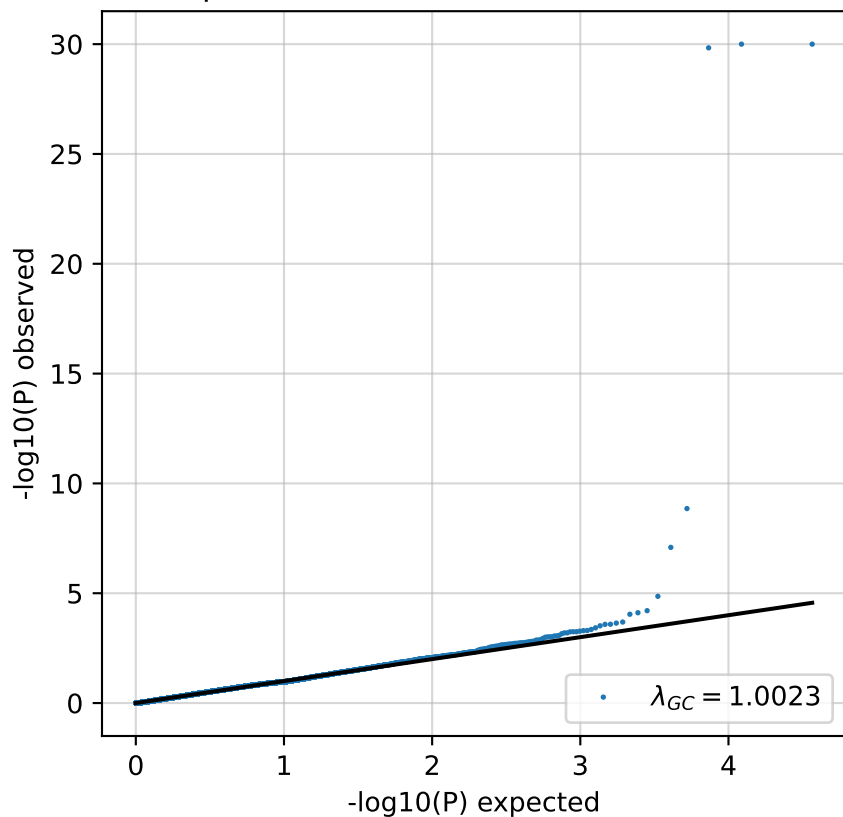

Supplement: Supplementary file 8 — Supplementary Data 5 [file 41467_2022_32864_MOESM8_ESM.zip › qqplots/miss_K_sLRT_Apolipoprotein_B.pdf]

Aspartate\_aminotransferase, test type: K,  
implementation: sLRT, var. effect: miss

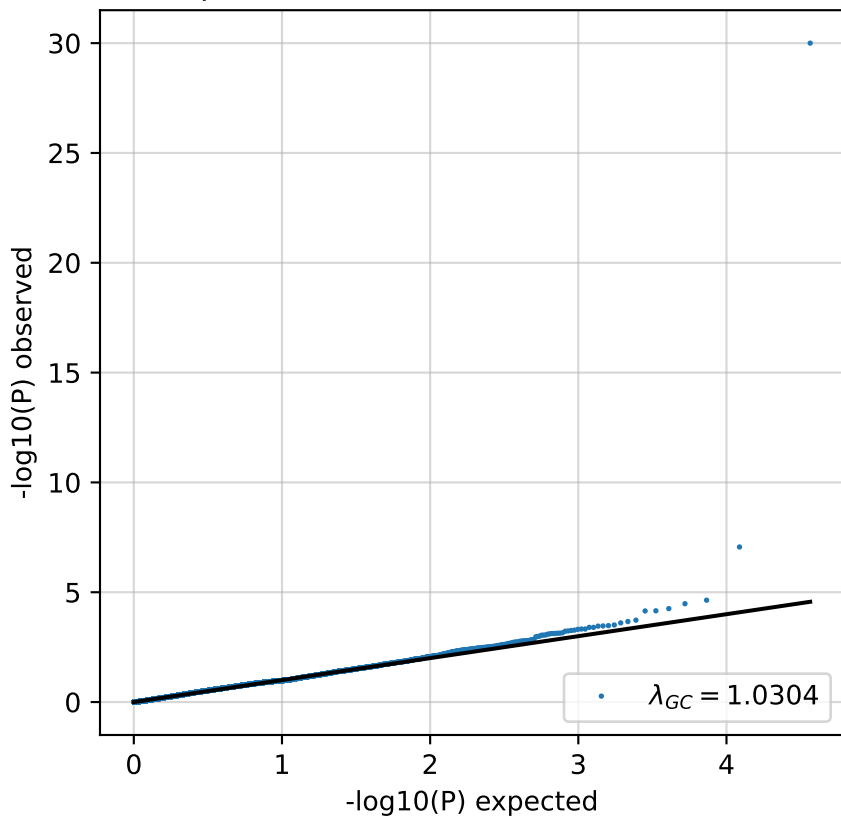

Supplement: Supplementary file 8 — Supplementary Data 5 [file 41467_2022_32864_MOESM8_ESM.zip › qqplots/miss_K_sLRT_Aspartate_aminotransferase.pdf]

Calcium, test type: K,  
implementation: sLRT, var. effect: miss

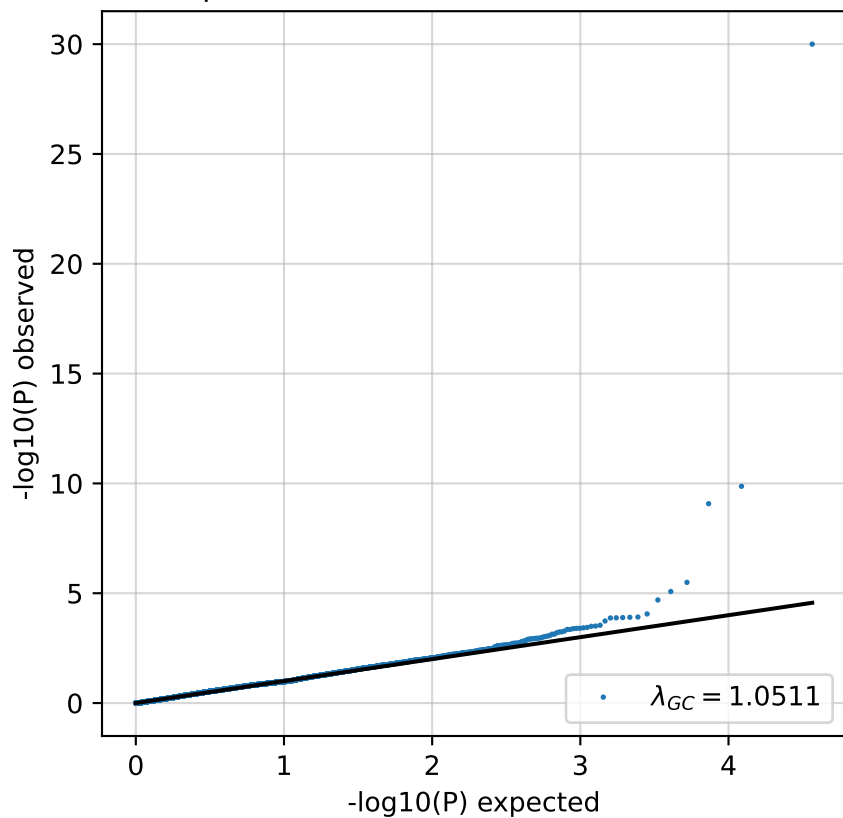

Supplement: Supplementary file 8 — Supplementary Data 5 [file 41467_2022_32864_MOESM8_ESM.zip › qqplots/miss_K_sLRT_Calcium.pdf]

Cholesterol, test type: K,  
implementation: sLRT, var. effect: miss

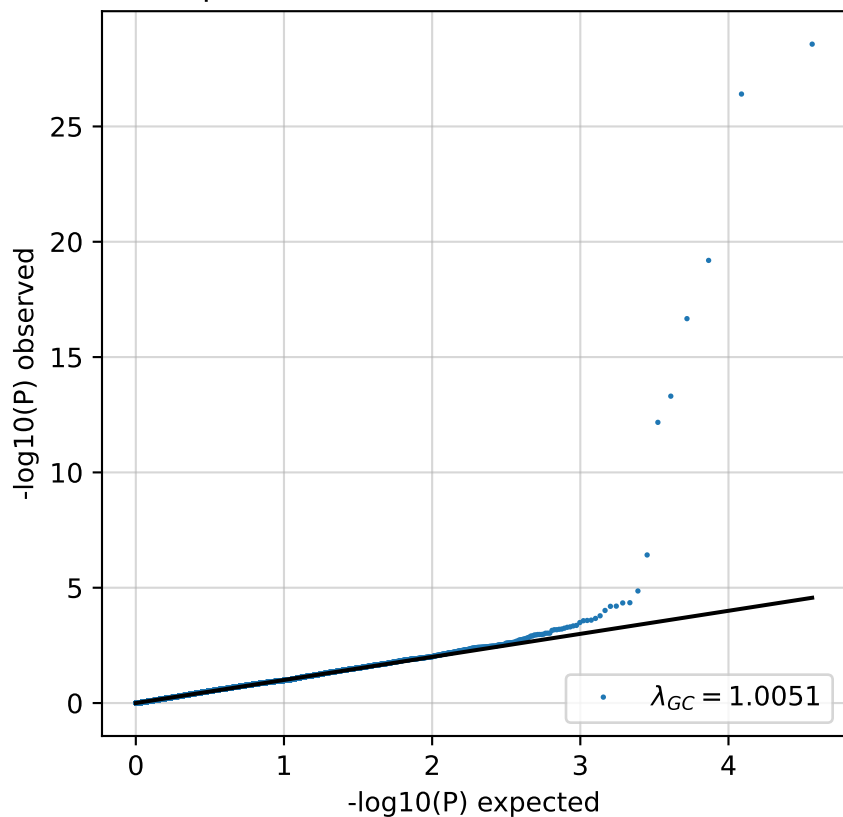

Supplement: Supplementary file 8 — Supplementary Data 5 [file 41467_2022_32864_MOESM8_ESM.zip › qqplots/miss_K_sLRT_Cholesterol.pdf]

C-reactive\_protein, test type: K,  
implementation: sLRT, var. effect: miss

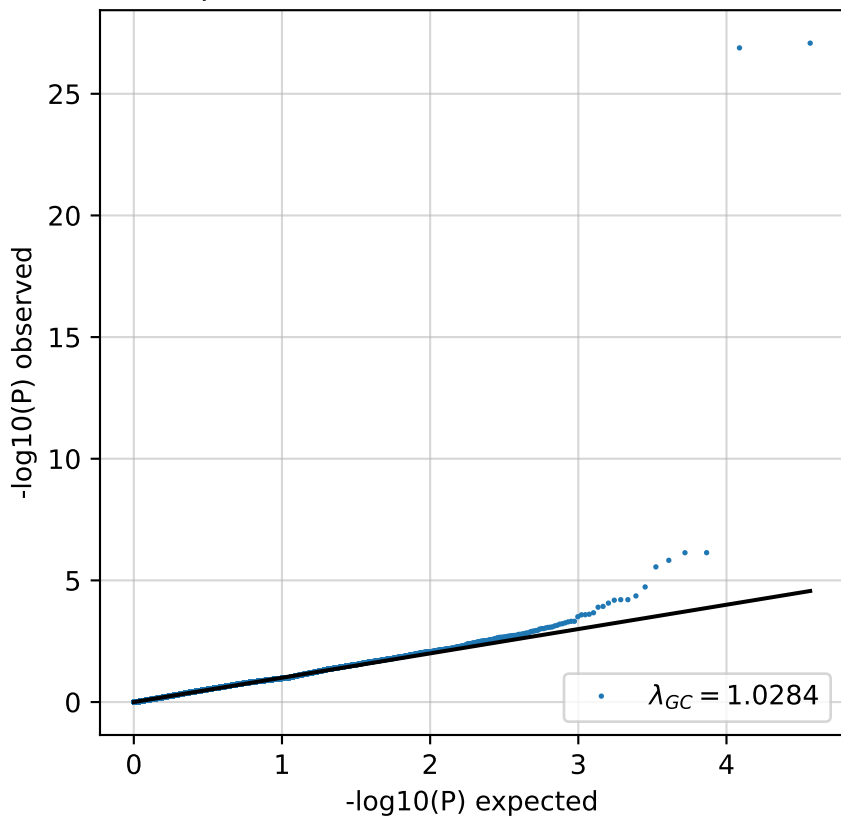

Supplement: Supplementary file 8 — Supplementary Data 5 [file 41467_2022_32864_MOESM8_ESM.zip › qqplots/miss_K_sLRT_Creactive_protein.pdf]

Creatinine, test type: K,  
implementation: sLRT, var. effect: miss

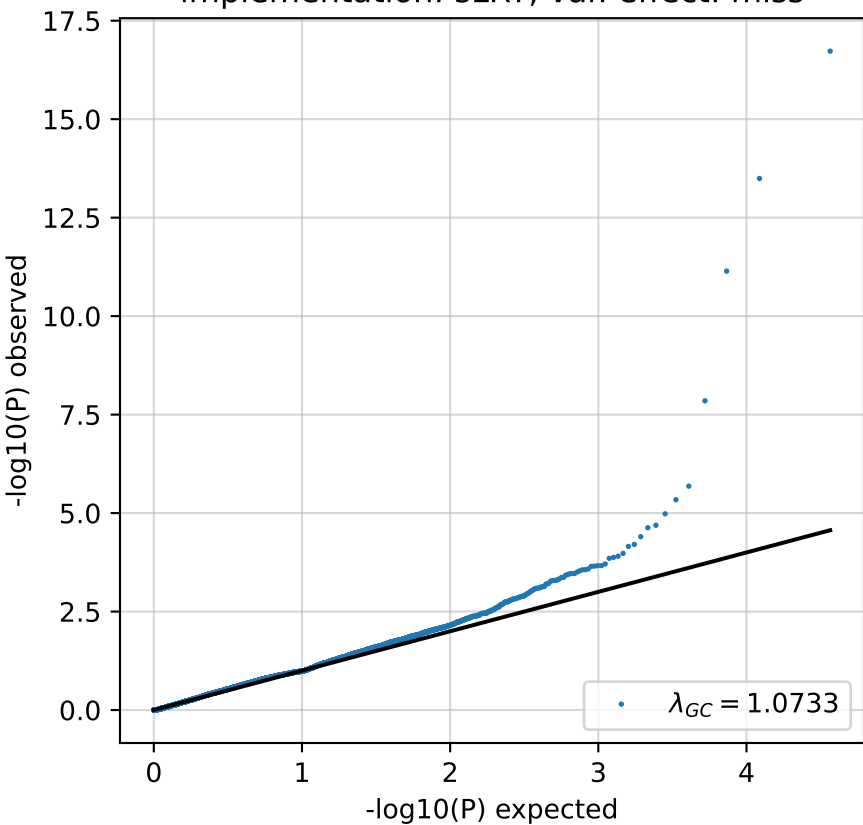

Supplement: Supplementary file 8 — Supplementary Data 5 [file 41467_2022_32864_MOESM8_ESM.zip › qqplots/miss_K_sLRT_Creatinine.pdf]

Cystatin\_C, test type: K,  
implementation: sLRT, var. effect: miss

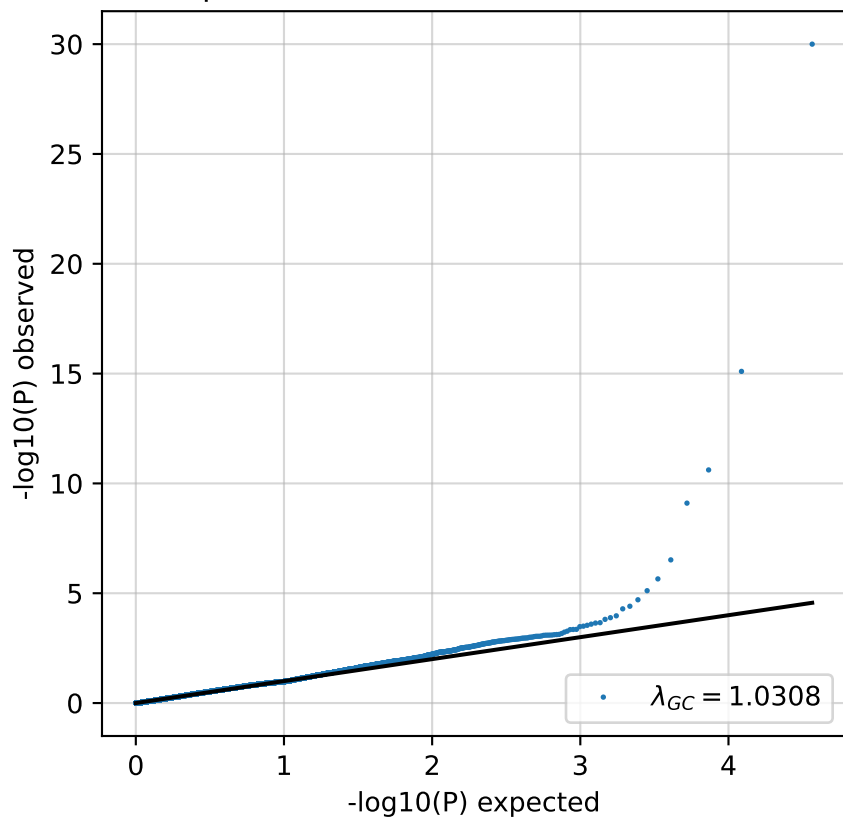

Supplement: Supplementary file 8 — Supplementary Data 5 [file 41467_2022_32864_MOESM8_ESM.zip › qqplots/miss_K_sLRT_Cystatin_C.pdf]

Direct\_bilirubin, test type: K,  
implementation: sLRT, var. effect: miss

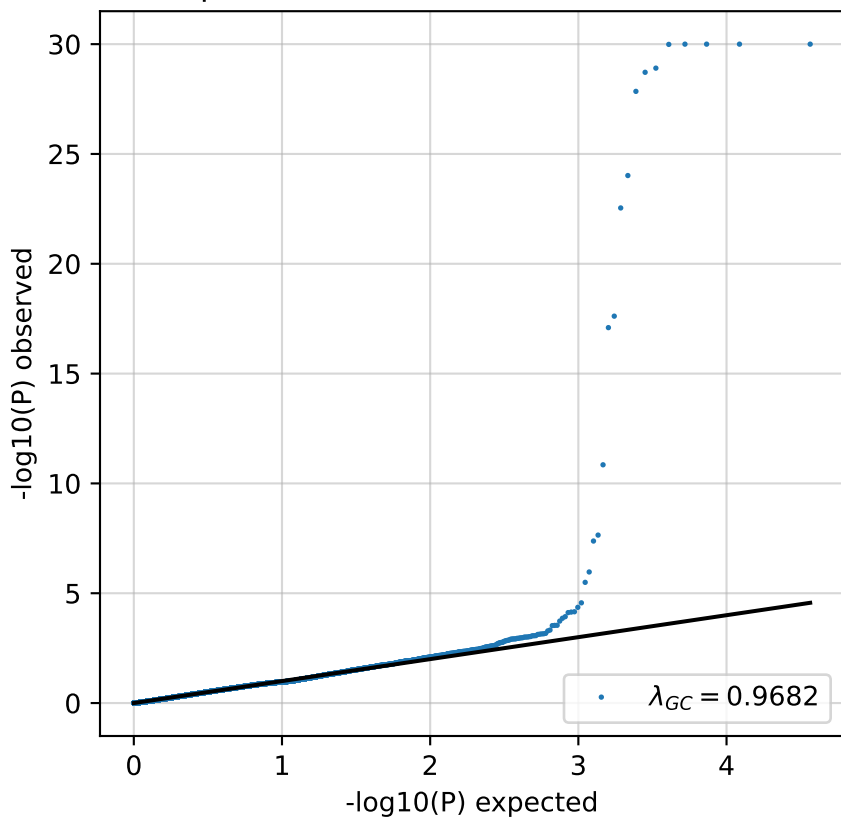

Supplement: Supplementary file 8 — Supplementary Data 5 [file 41467_2022_32864_MOESM8_ESM.zip › qqplots/miss_K_sLRT_Direct_bilirubin.pdf]

Gamma\_glutamyltransferase, test type: K,  
implementation: sLRT, var. effect: miss

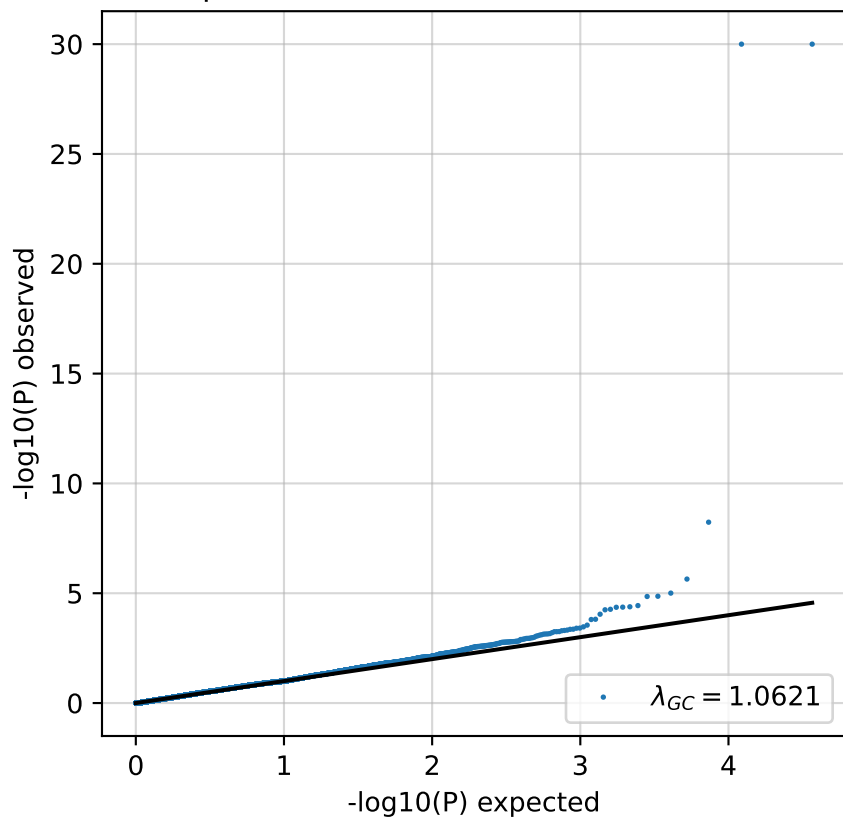

Supplement: Supplementary file 8 — Supplementary Data 5 [file 41467_2022_32864_MOESM8_ESM.zip › qqplots/miss_K_sLRT_Gamma_glutamyltransferase.pdf]

Glucose, test type: K,  
implementation: sLRT, var. effect: miss

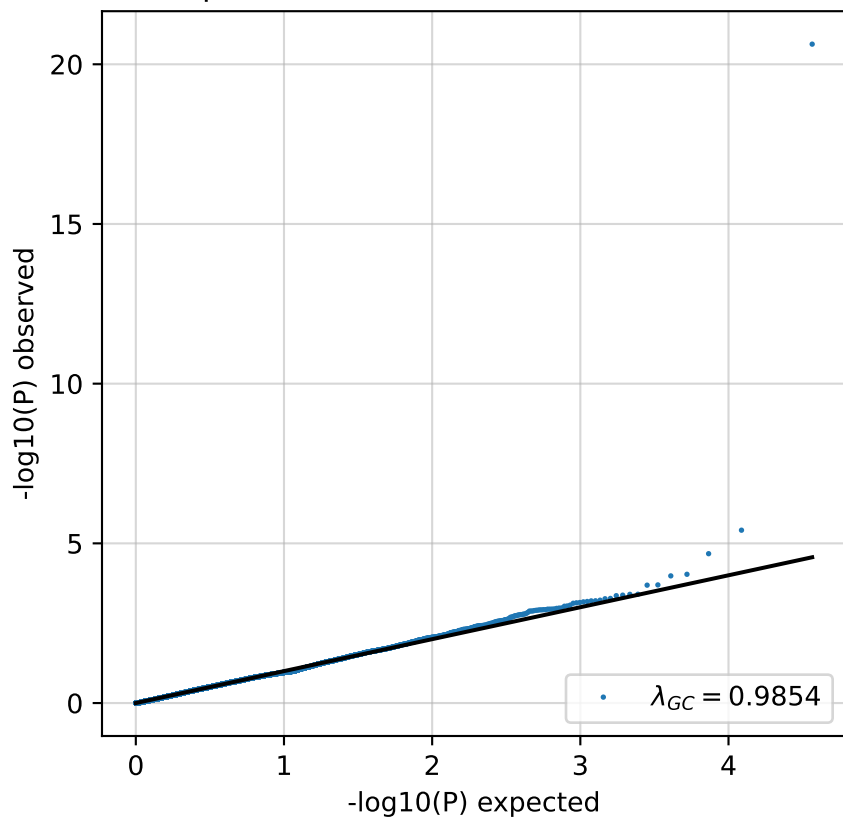

Supplement: Supplementary file 8 — Supplementary Data 5 [file 41467_2022_32864_MOESM8_ESM.zip › qqplots/miss_K_sLRT_Glucose.pdf]

Glycated\_haemoglobin\_(HbA1c), test type: K,  
implementation: sLRT, var. effect: miss

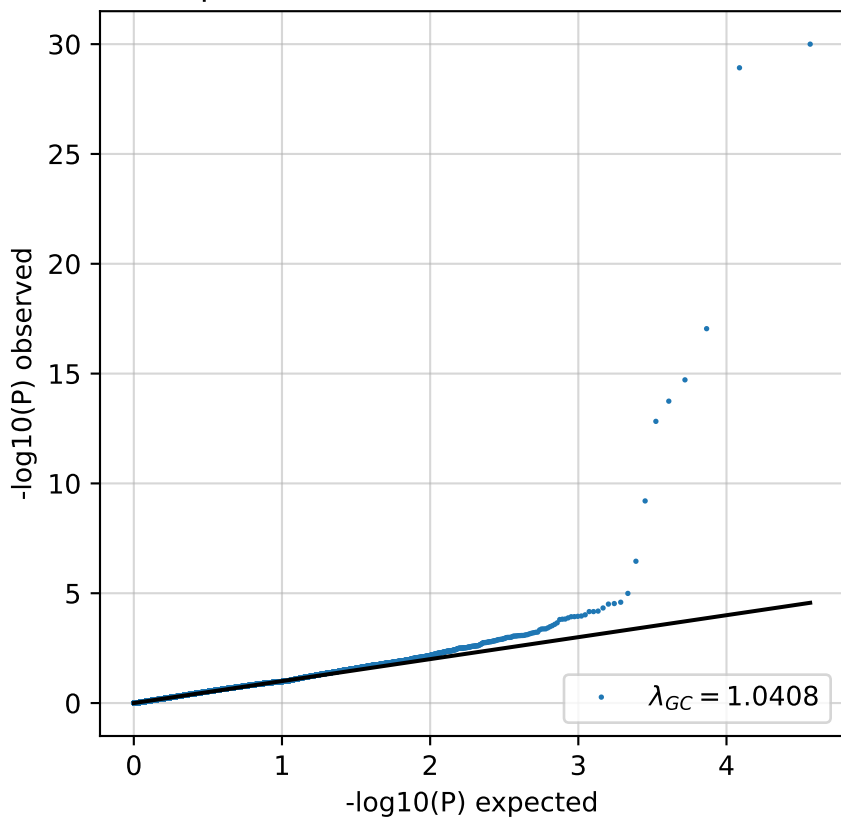

Supplement: Supplementary file 8 — Supplementary Data 5 [file 41467_2022_32864_MOESM8_ESM.zip › qqplots/miss_K_sLRT_Glycated_haemoglobin_HbA1c.pdf]

HDL\_cholesterol, test type: K,  
implementation: sLRT, var. effect: miss

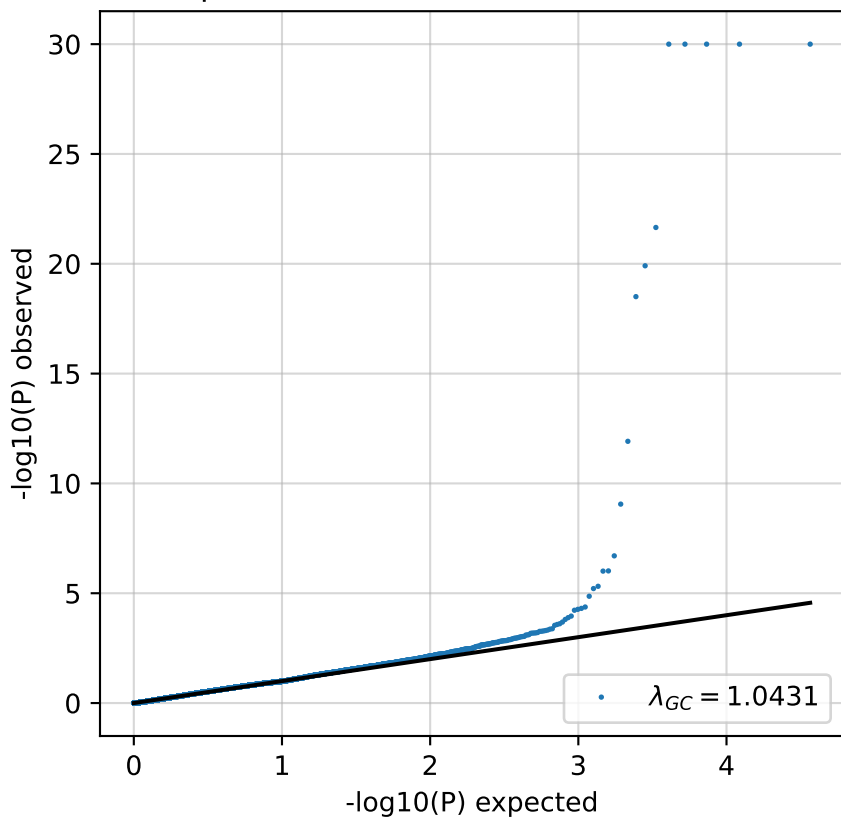

Supplement: Supplementary file 8 — Supplementary Data 5 [file 41467_2022_32864_MOESM8_ESM.zip › qqplots/miss_K_sLRT_HDL_cholesterol.pdf]

IGF-1, test type: K,  
implementation: sLRT, var. effect: miss

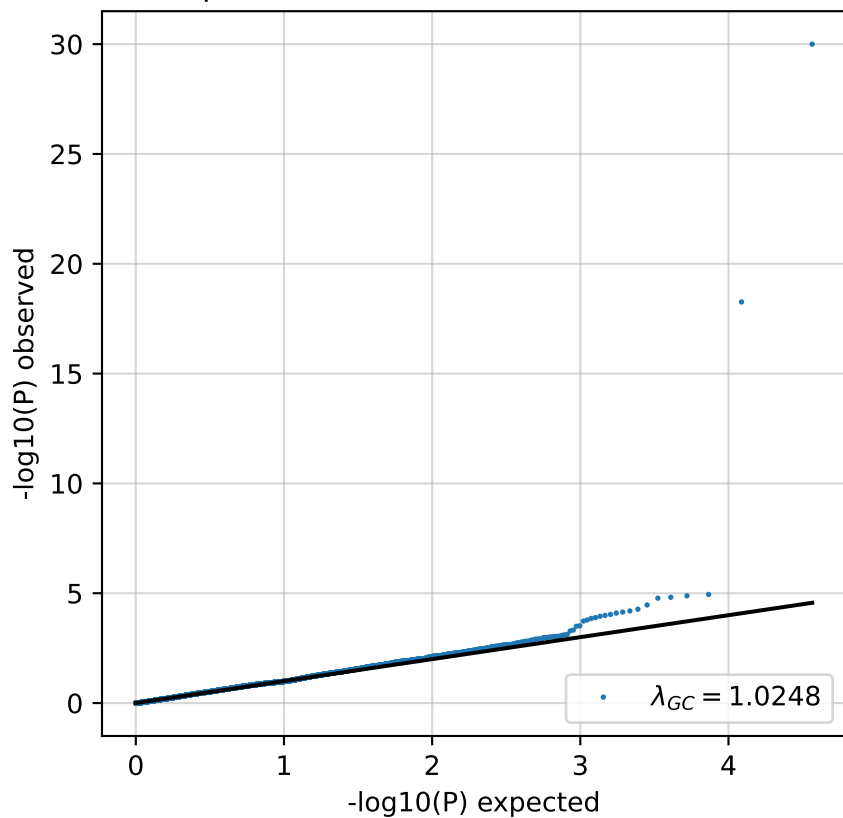

Supplement: Supplementary file 8 — Supplementary Data 5 [file 41467_2022_32864_MOESM8_ESM.zip › qqplots/miss_K_sLRT_IGF1.pdf]

LDL\_direct, test type: K,  
implementation: sLRT, var. effect: miss

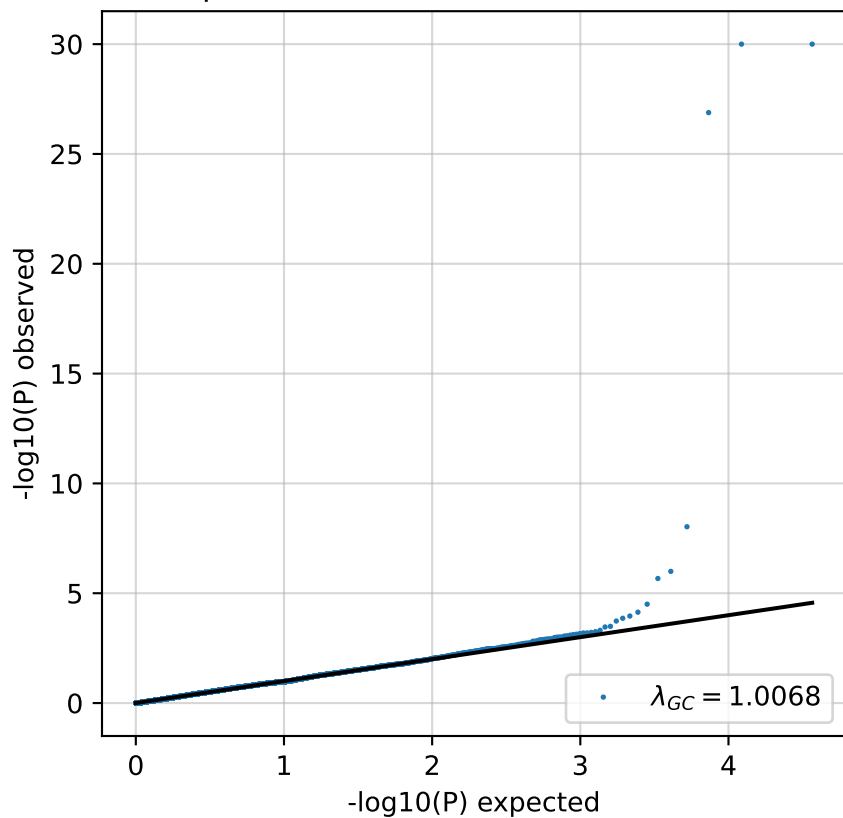

Supplement: Supplementary file 8 — Supplementary Data 5 [file 41467_2022_32864_MOESM8_ESM.zip › qqplots/miss_K_sLRT_LDL_direct.pdf]

Lipoprotein\_A, test type: K,  
implementation: sLRT, var. effect: miss

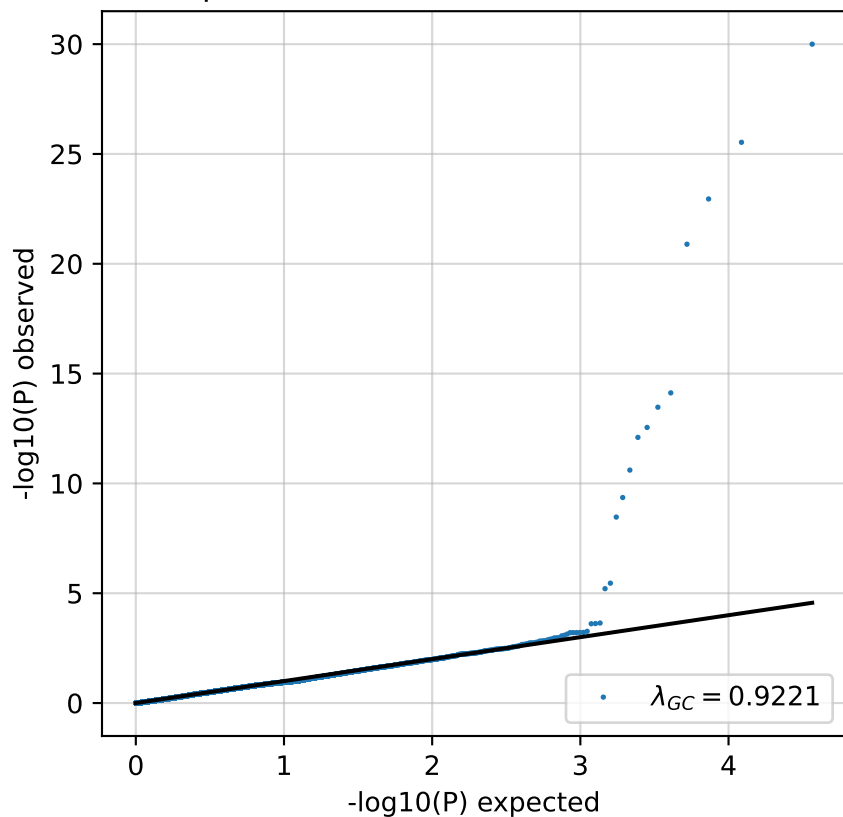

Supplement: Supplementary file 8 — Supplementary Data 5 [file 41467_2022_32864_MOESM8_ESM.zip › qqplots/miss_K_sLRT_Lipoprotein_A.pdf]

Phosphate, test type: K,  
implementation: sLRT, var. effect: miss

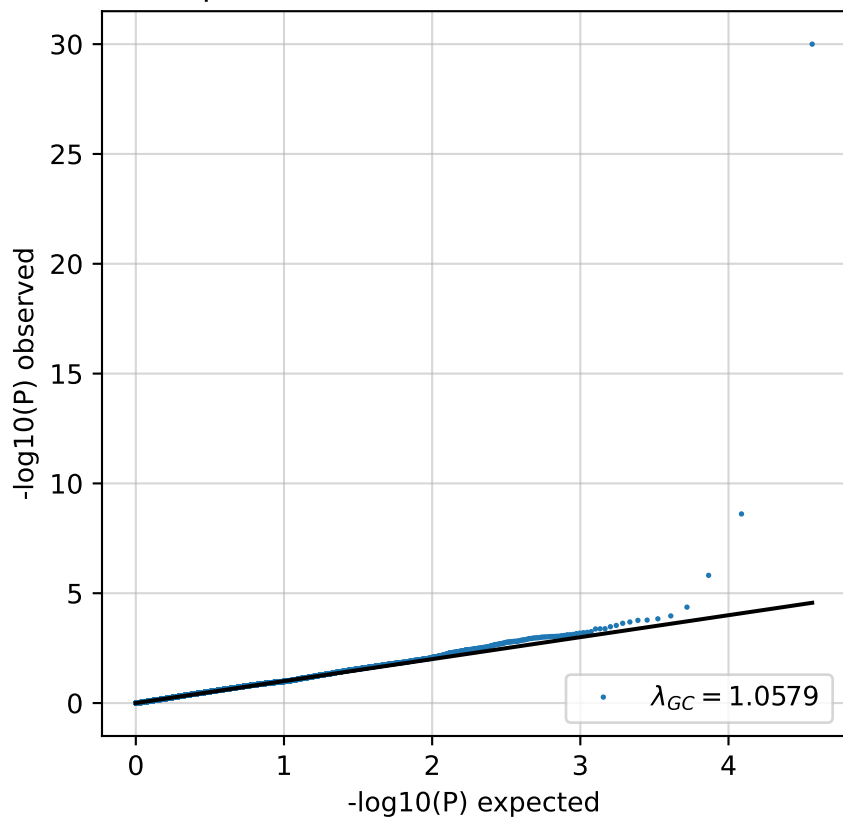

Supplement: Supplementary file 8 — Supplementary Data 5 [file 41467_2022_32864_MOESM8_ESM.zip › qqplots/miss_K_sLRT_Phosphate.pdf]

SHBG, test type: K,  
implementation: sLRT, var. effect: miss

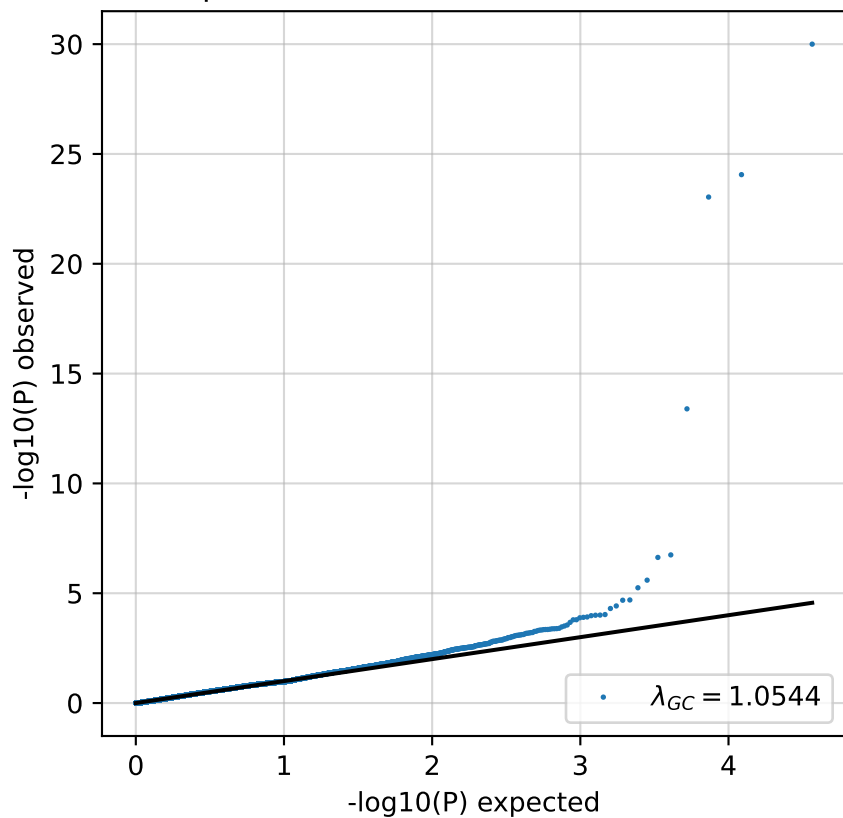

Supplement: Supplementary file 8 — Supplementary Data 5 [file 41467_2022_32864_MOESM8_ESM.zip › qqplots/miss_K_sLRT_SHBG.pdf]

Total\_bilirubin, test type: K,  
implementation: sLRT, var. effect: miss

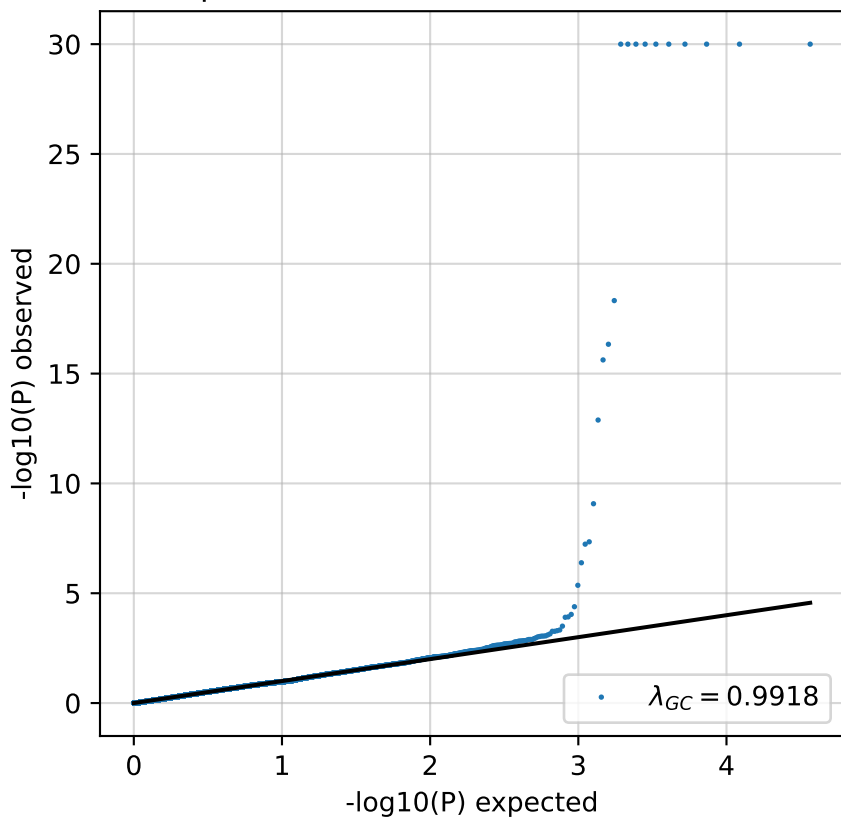

Supplement: Supplementary file 8 — Supplementary Data 5 [file 41467_2022_32864_MOESM8_ESM.zip › qqplots/miss_K_sLRT_Total_bilirubin.pdf]

Total\_protein, test type: K,  
implementation: sLRT, var. effect: miss

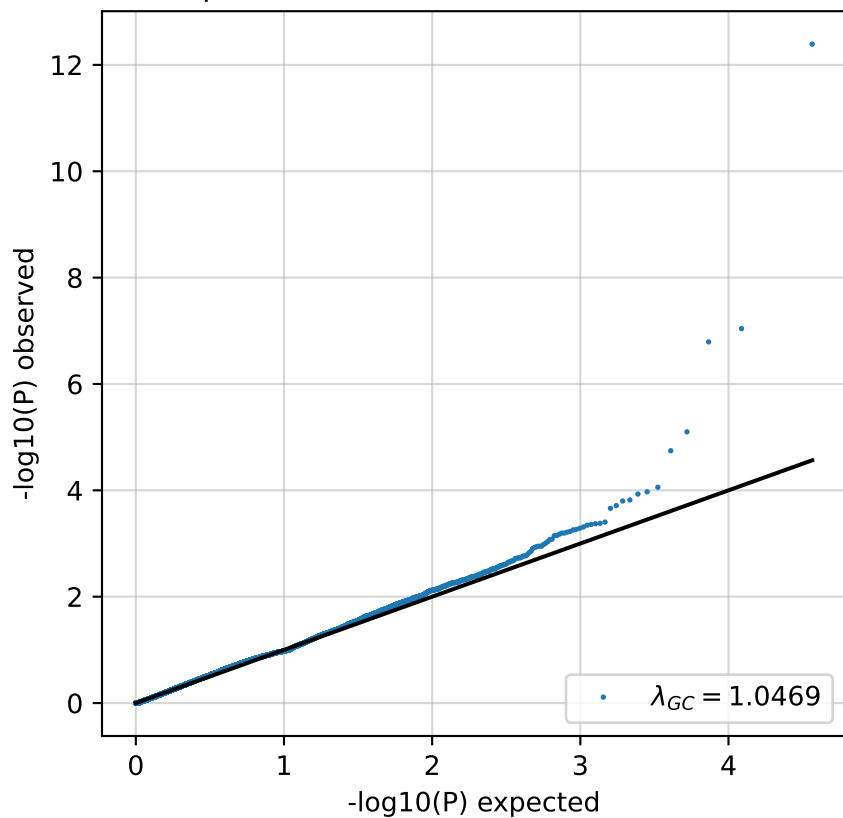

Supplement: Supplementary file 8 — Supplementary Data 5 [file 41467_2022_32864_MOESM8_ESM.zip › qqplots/miss_K_sLRT_Total_protein.pdf]

Triglycerides, test type: K,  
implementation: sLRT, var. effect: miss

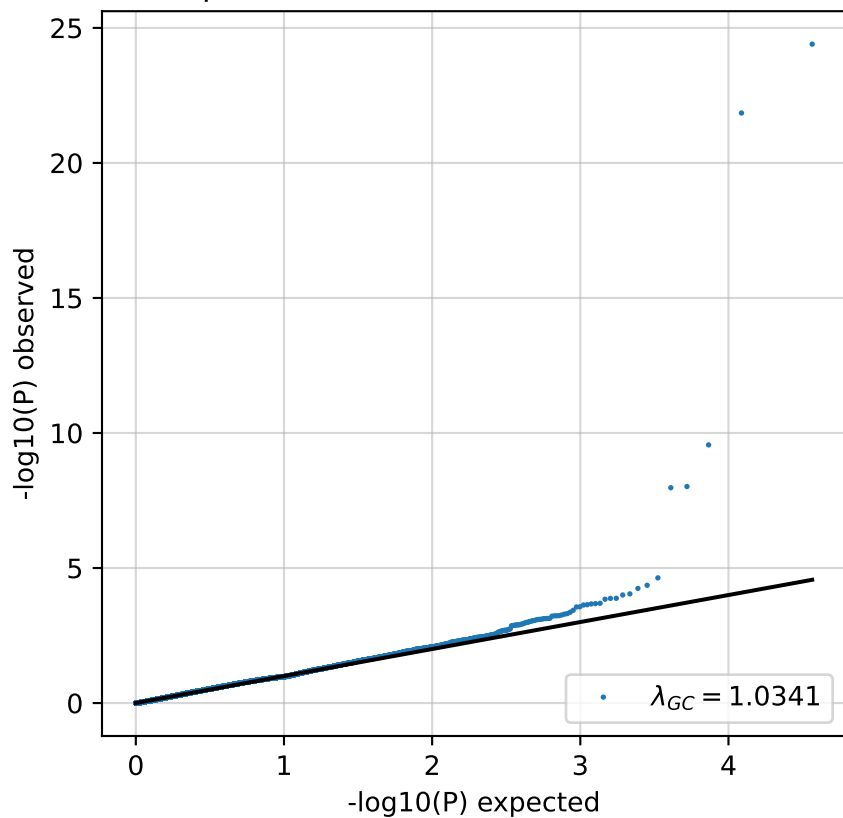

Supplement: Supplementary file 8 — Supplementary Data 5 [file 41467_2022_32864_MOESM8_ESM.zip › qqplots/miss_K_sLRT_Triglycerides.pdf]

Urate, test type: K,  
implementation: sLRT, var. effect: miss

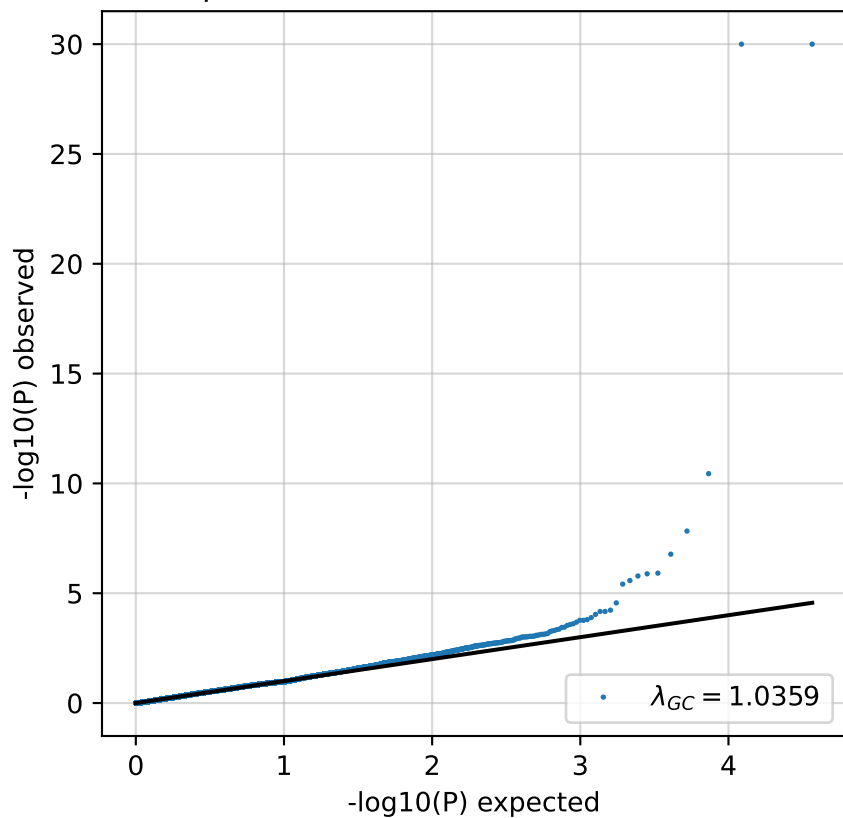

Supplement: Supplementary file 8 — Supplementary Data 5 [file 41467_2022_32864_MOESM8_ESM.zip › qqplots/miss_K_sLRT_Urate.pdf]

Vitamin\_D, test type: K,  
implementation: sLRT, var. effect: miss

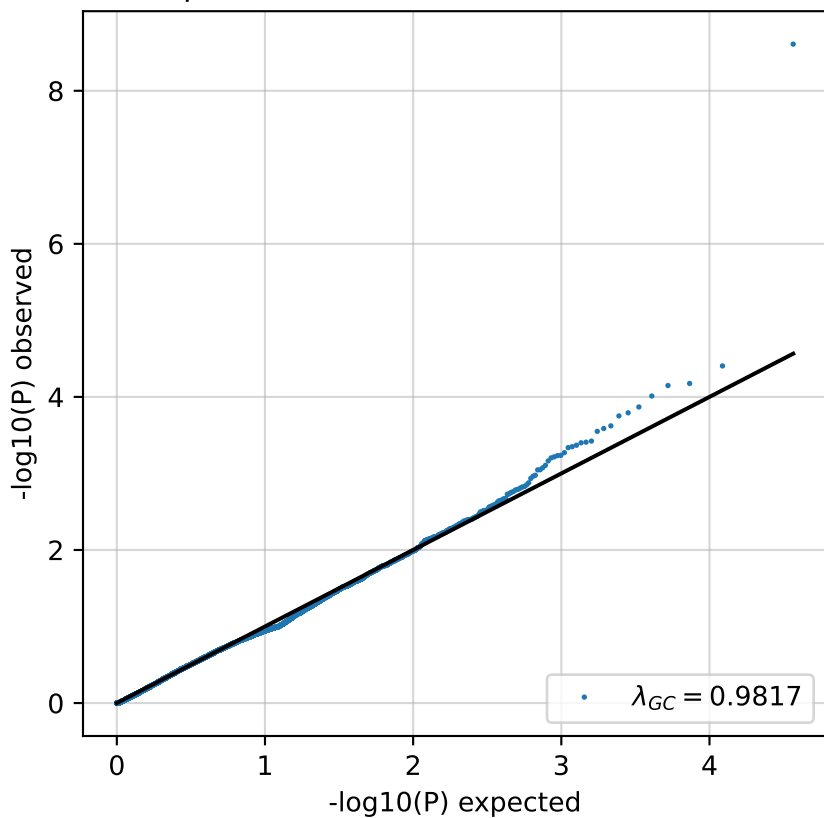

Supplement: Supplementary file 8 — Supplementary Data 5 [file 41467_2022_32864_MOESM8_ESM.zip › qqplots/miss_K_sLRT_Vitamin_D.pdf]

Alanine\_aminotransferase, test type: gbvc,  
implementation: sLRT, var. effect: miss

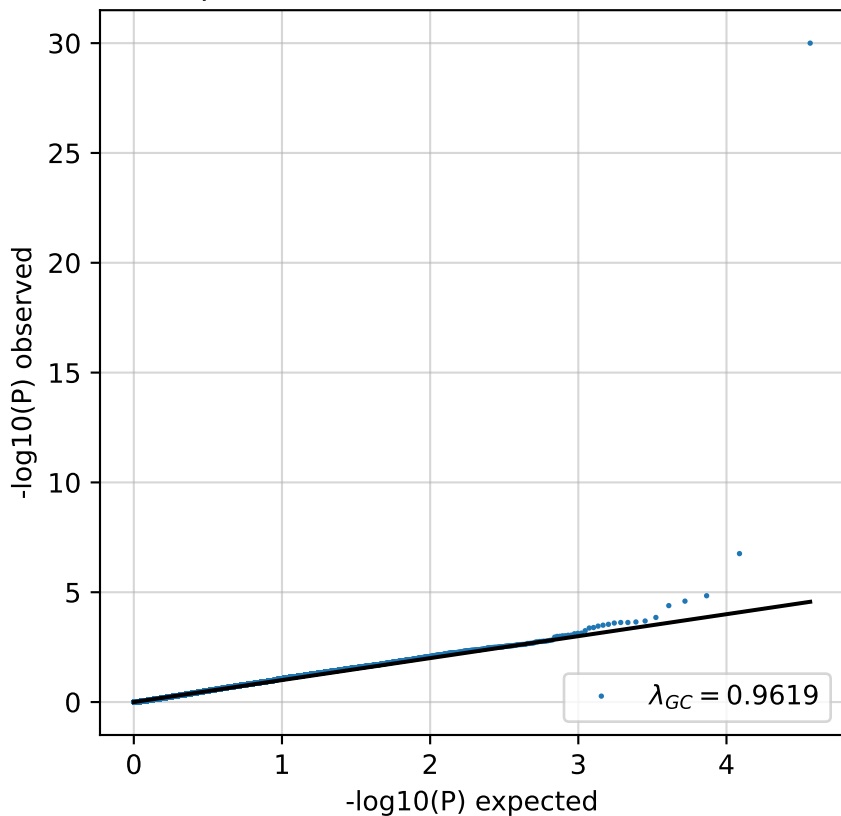

Supplement: Supplementary file 8 — Supplementary Data 5 [file 41467_2022_32864_MOESM8_ESM.zip › qqplots/miss_gbvc_sLRT_Alanine_aminotransferase.pdf]

Albumin, test type: gbvc,  
implementation: sLRT, var. effect: miss

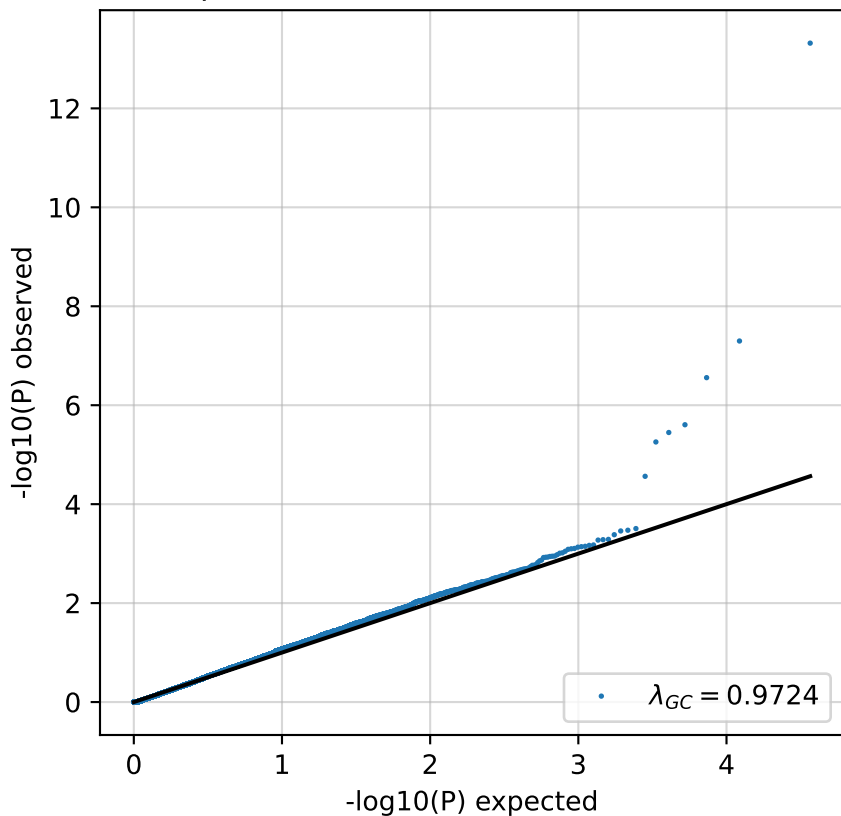

Supplement: Supplementary file 8 — Supplementary Data 5 [file 41467_2022_32864_MOESM8_ESM.zip › qqplots/miss_gbvc_sLRT_Albumin.pdf]

Alkaline\_phosphatase, test type: gbvc,  
implementation: sLRT, var. effect: miss

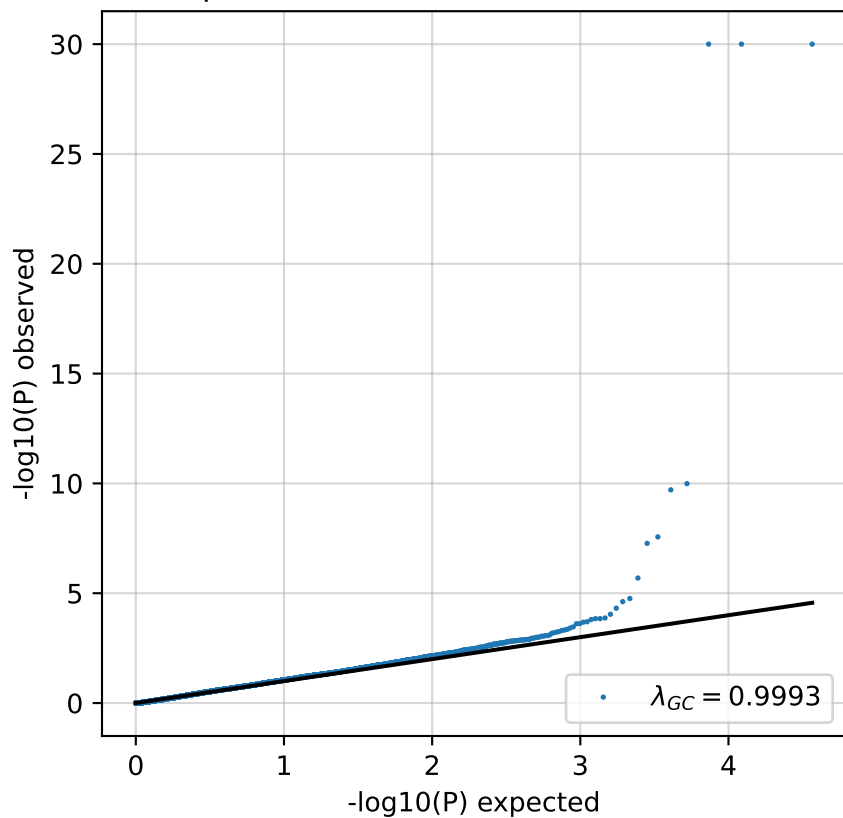

Supplement: Supplementary file 8 — Supplementary Data 5 [file 41467_2022_32864_MOESM8_ESM.zip › qqplots/miss_gbvc_sLRT_Alkaline_phosphatase.pdf]

Apolipoprotein\_A, test type: gbvc,  
implementation: sLRT, var. effect: miss

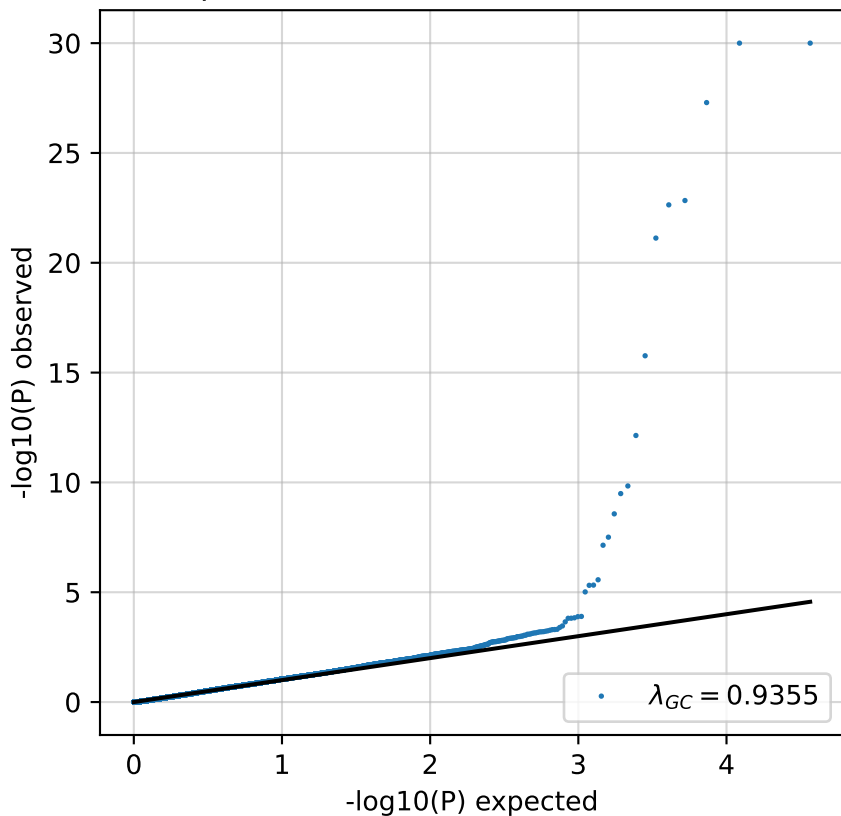

Supplement: Supplementary file 8 — Supplementary Data 5 [file 41467_2022_32864_MOESM8_ESM.zip › qqplots/miss_gbvc_sLRT_Apolipoprotein_A.pdf]

Apolipoprotein\_B, test type: gbvc,  
implementation: sLRT, var. effect: miss

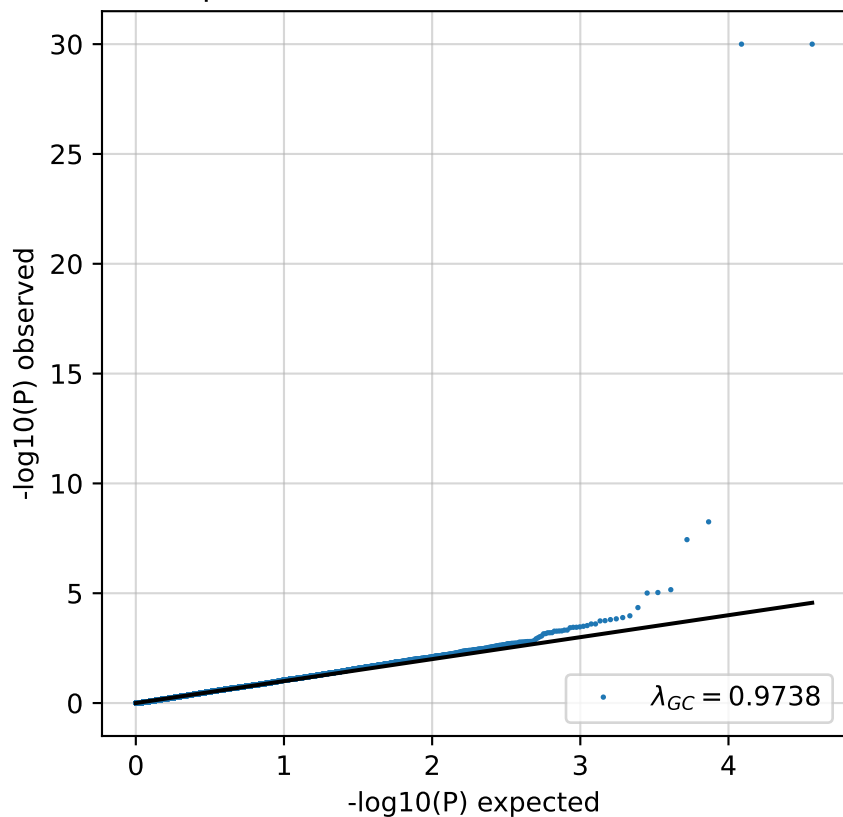

Supplement: Supplementary file 8 — Supplementary Data 5 [file 41467_2022_32864_MOESM8_ESM.zip › qqplots/miss_gbvc_sLRT_Apolipoprotein_B.pdf]

Aspartate\_aminotransferase, test type: gbvc,  
implementation: sLRT, var. effect: miss

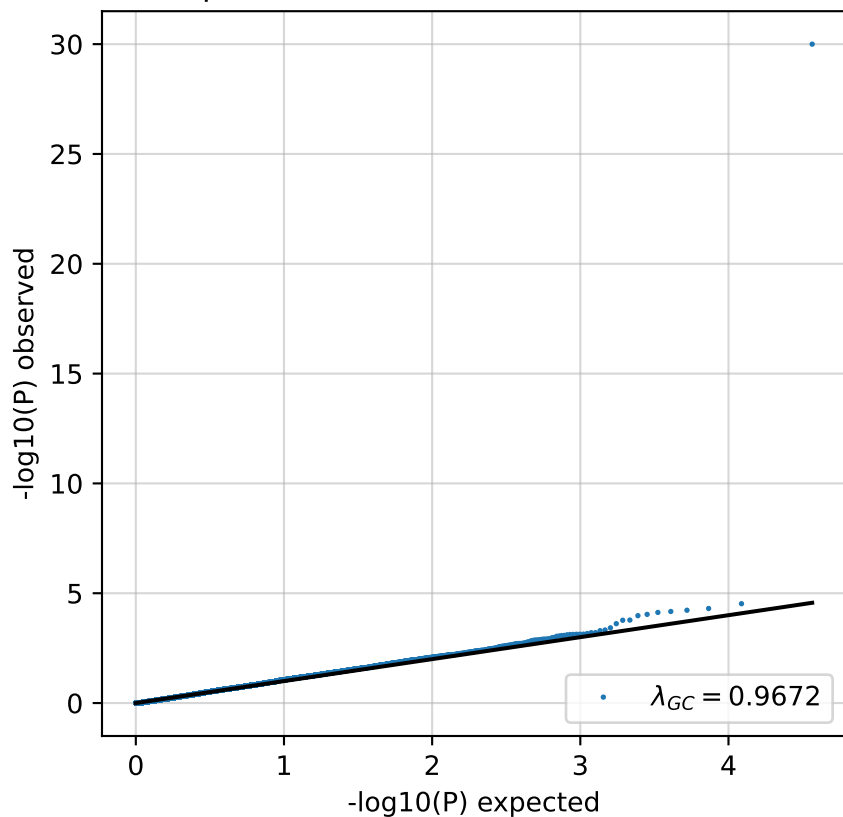

Supplement: Supplementary file 8 — Supplementary Data 5 [file 41467_2022_32864_MOESM8_ESM.zip › qqplots/miss_gbvc_sLRT_Aspartate_aminotransferase.pdf]

Calcium, test type: gbvc,  
implementation: sLRT, var. effect: miss

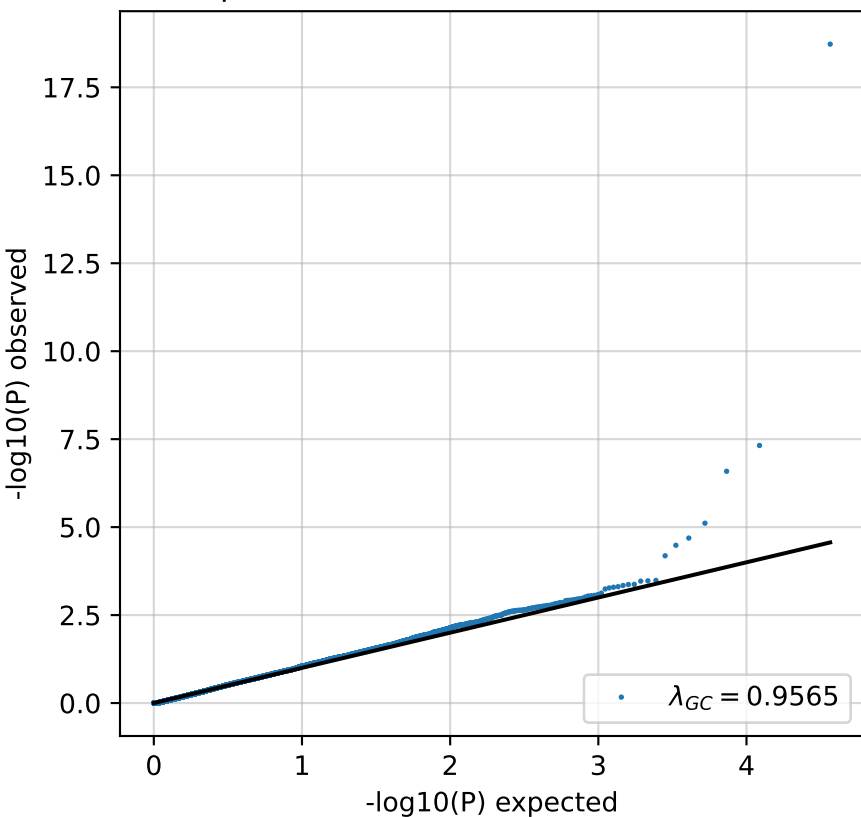

Supplement: Supplementary file 8 — Supplementary Data 5 [file 41467_2022_32864_MOESM8_ESM.zip › qqplots/miss_gbvc_sLRT_Calcium.pdf]

Cholesterol, test type: gbvc,  
implementation: sLRT, var. effect: miss

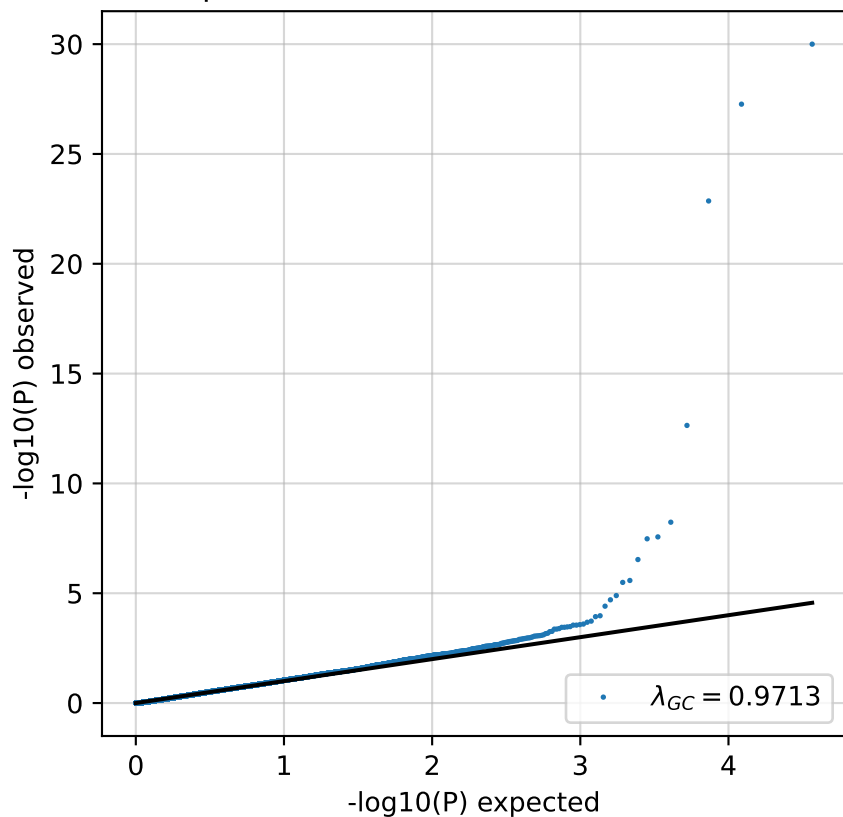

Supplement: Supplementary file 8 — Supplementary Data 5 [file 41467_2022_32864_MOESM8_ESM.zip › qqplots/miss_gbvc_sLRT_Cholesterol.pdf]

C-reactive\_protein, test type: gbvc,  
implementation: sLRT, var. effect: miss

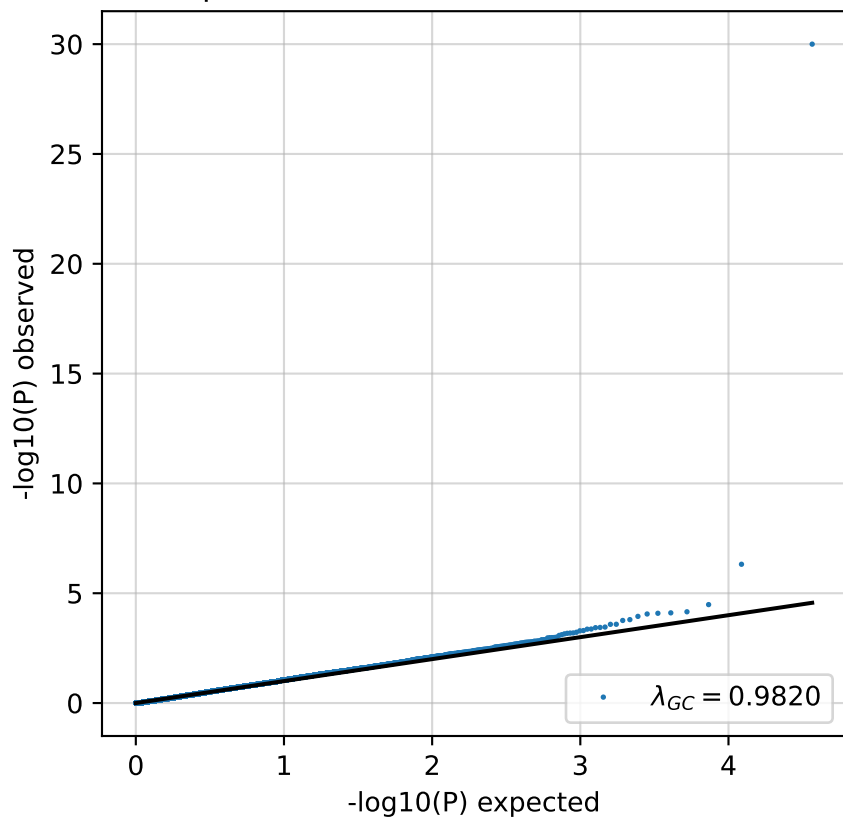

Supplement: Supplementary file 8 — Supplementary Data 5 [file 41467_2022_32864_MOESM8_ESM.zip › qqplots/miss_gbvc_sLRT_Creactive_protein.pdf]

Creatinine, test type: gbvc,  
implementation: sLRT, var. effect: miss

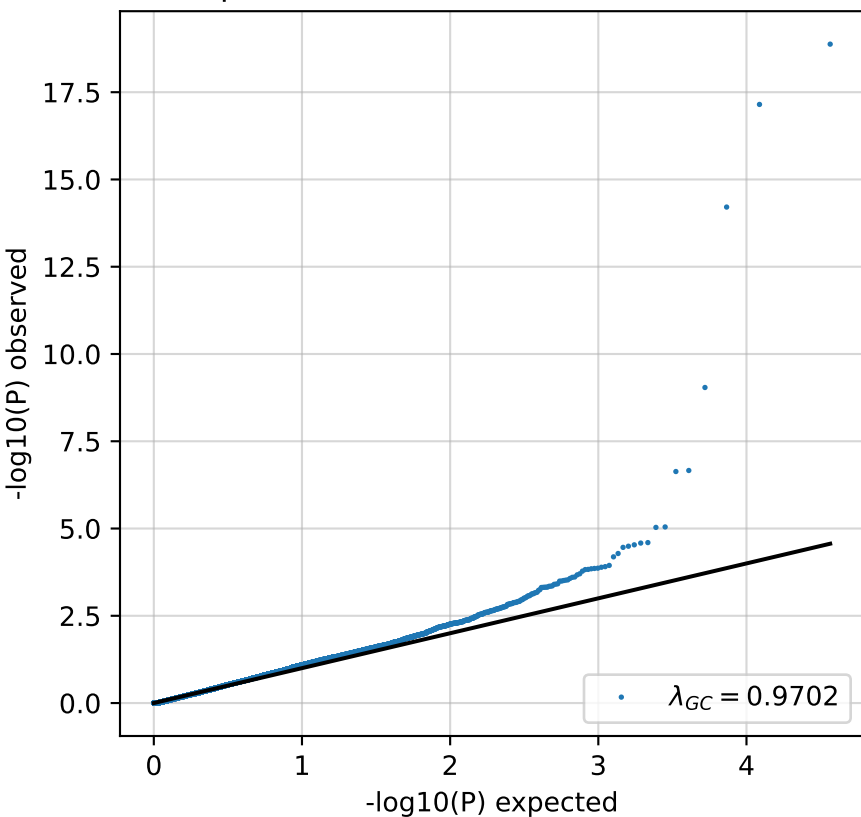

Supplement: Supplementary file 8 — Supplementary Data 5 [file 41467_2022_32864_MOESM8_ESM.zip › qqplots/miss_gbvc_sLRT_Creatinine.pdf]

Cystatin\_C, test type: gbvc,  
implementation: sLRT, var. effect: miss

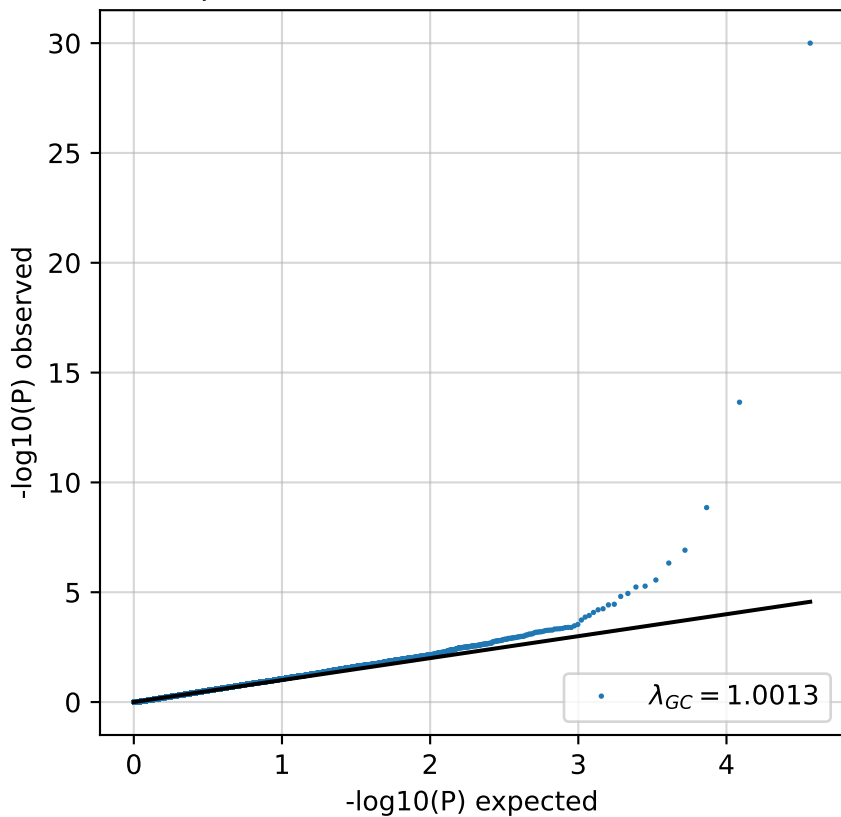

Supplement: Supplementary file 8 — Supplementary Data 5 [file 41467_2022_32864_MOESM8_ESM.zip › qqplots/miss_gbvc_sLRT_Cystatin_C.pdf]

Direct\_bilirubin, test type: gbvc,  
implementation: sLRT, var. effect: miss

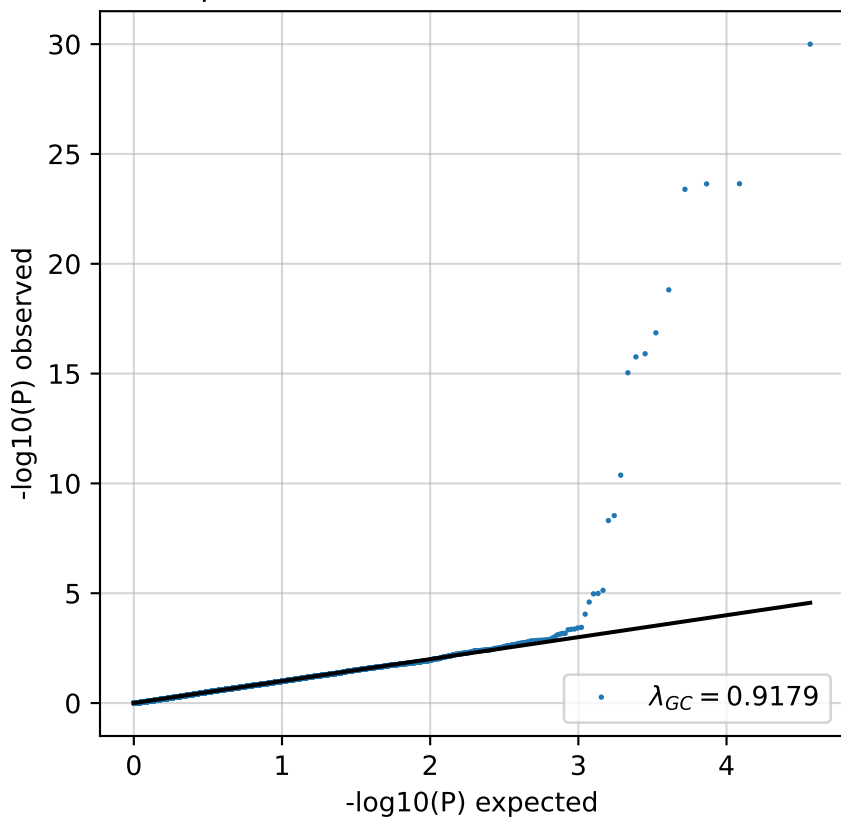

Supplement: Supplementary file 8 — Supplementary Data 5 [file 41467_2022_32864_MOESM8_ESM.zip › qqplots/miss_gbvc_sLRT_Direct_bilirubin.pdf]

Gamma\_glutamyltransferase, test type: gbvc,  
implementation: sLRT, var. effect: miss

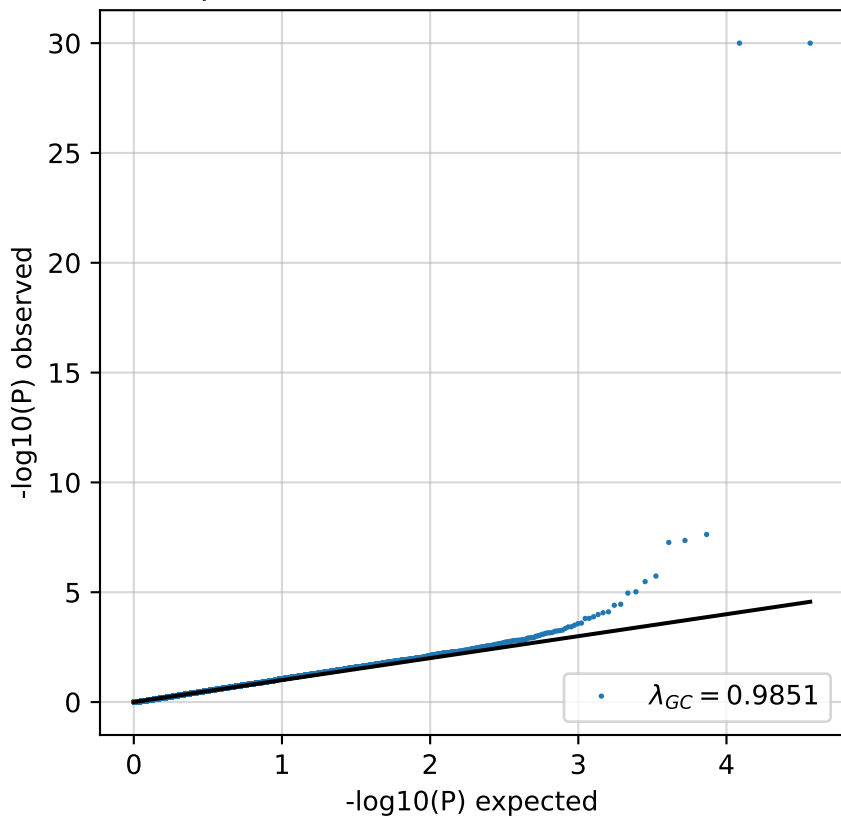

Supplement: Supplementary file 8 — Supplementary Data 5 [file 41467_2022_32864_MOESM8_ESM.zip › qqplots/miss_gbvc_sLRT_Gamma_glutamyltransferase.pdf]

Glucose, test type: gbvc,  
implementation: sLRT, var. effect: miss

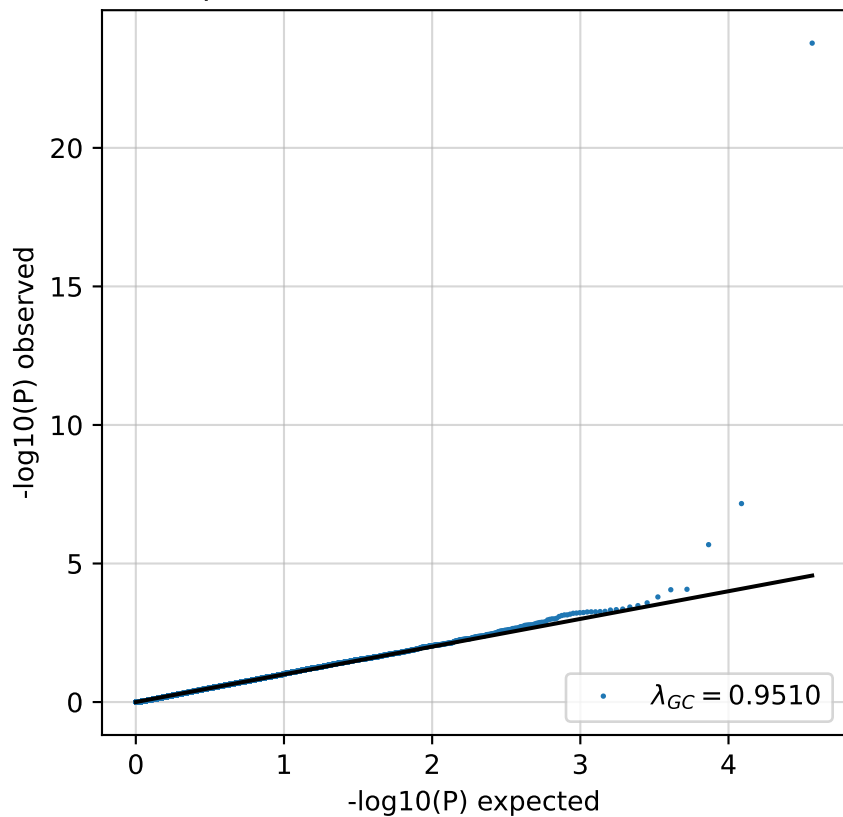

Supplement: Supplementary file 8 — Supplementary Data 5 [file 41467_2022_32864_MOESM8_ESM.zip › qqplots/miss_gbvc_sLRT_Glucose.pdf]

Glycated\_haemoglobin\_(HbA1c), test type: gbvc,  
implementation: sLRT, var. effect: miss

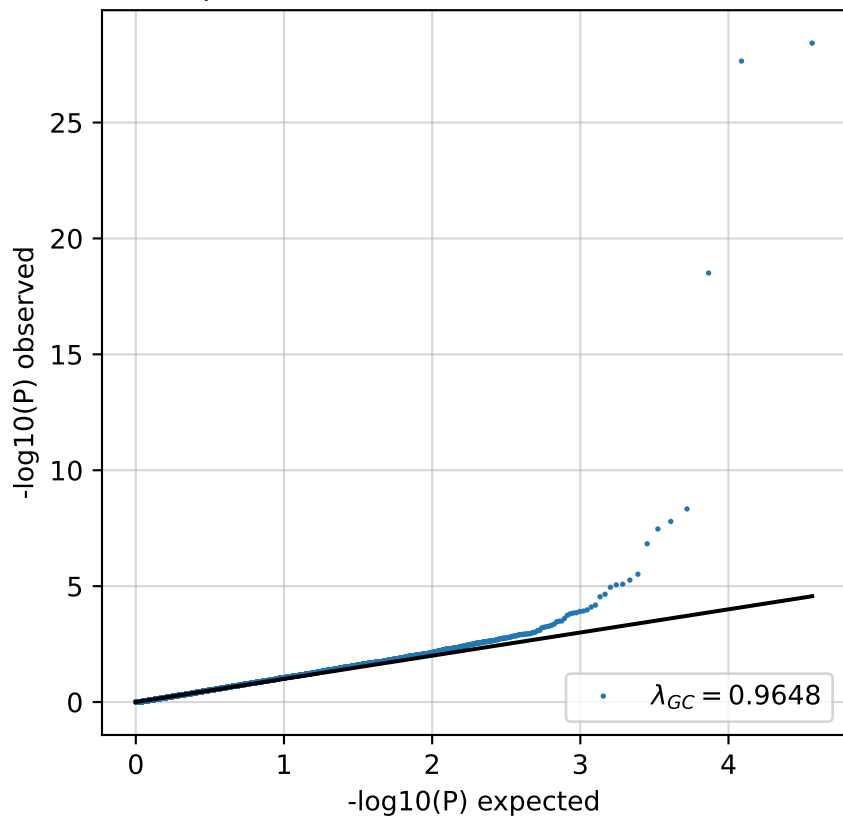

Supplement: Supplementary file 8 — Supplementary Data 5 [file 41467_2022_32864_MOESM8_ESM.zip › qqplots/miss_gbvc_sLRT_Glycated_haemoglobin_HbA1c.pdf]

HDL\_cholesterol, test type: gbvc,  
implementation: sLRT, var. effect: miss

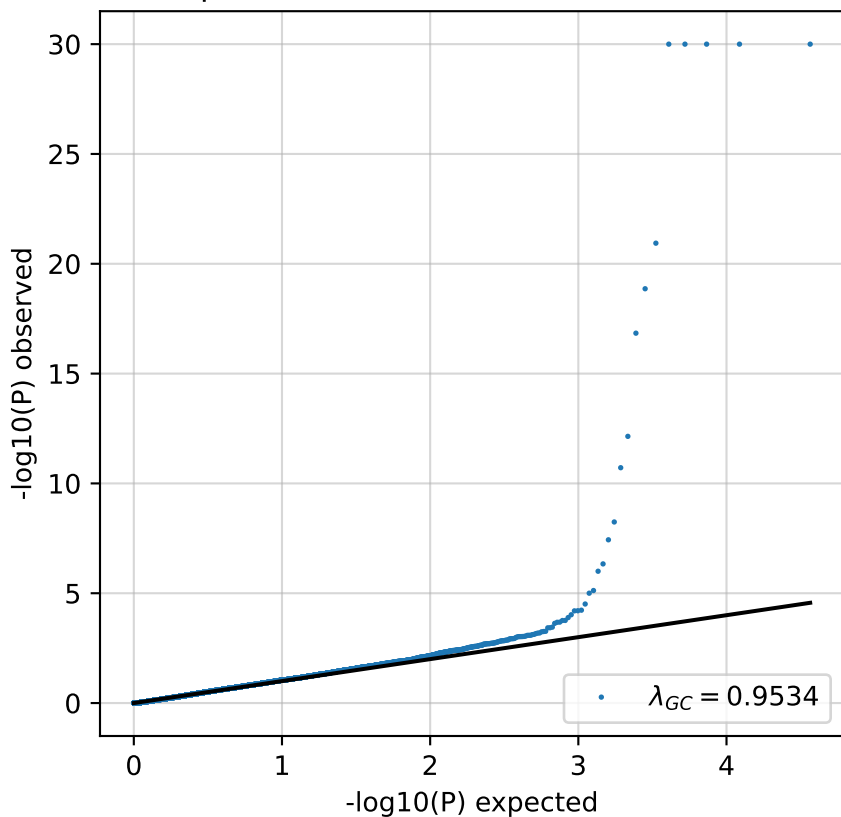

Supplement: Supplementary file 8 — Supplementary Data 5 [file 41467_2022_32864_MOESM8_ESM.zip › qqplots/miss_gbvc_sLRT_HDL_cholesterol.pdf]

IGF-1, test type: gbvc,  
implementation: sLRT, var. effect: miss

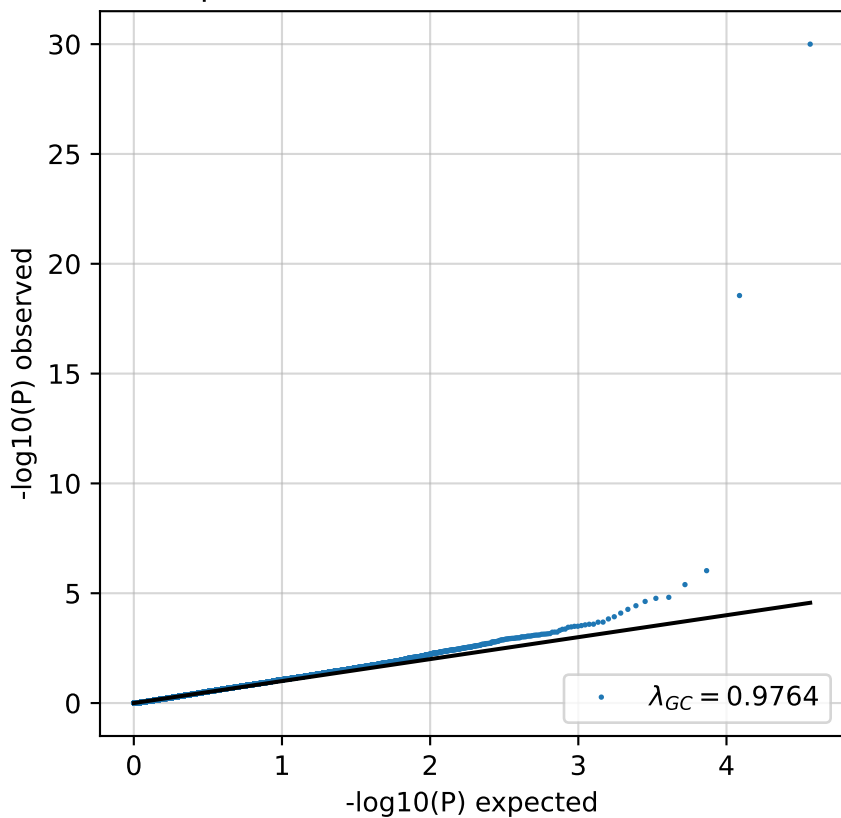

Supplement: Supplementary file 8 — Supplementary Data 5 [file 41467_2022_32864_MOESM8_ESM.zip › qqplots/miss_gbvc_sLRT_IGF1.pdf]

LDL\_direct, test type: gbvc,  
implementation: sLRT, var. effect: miss

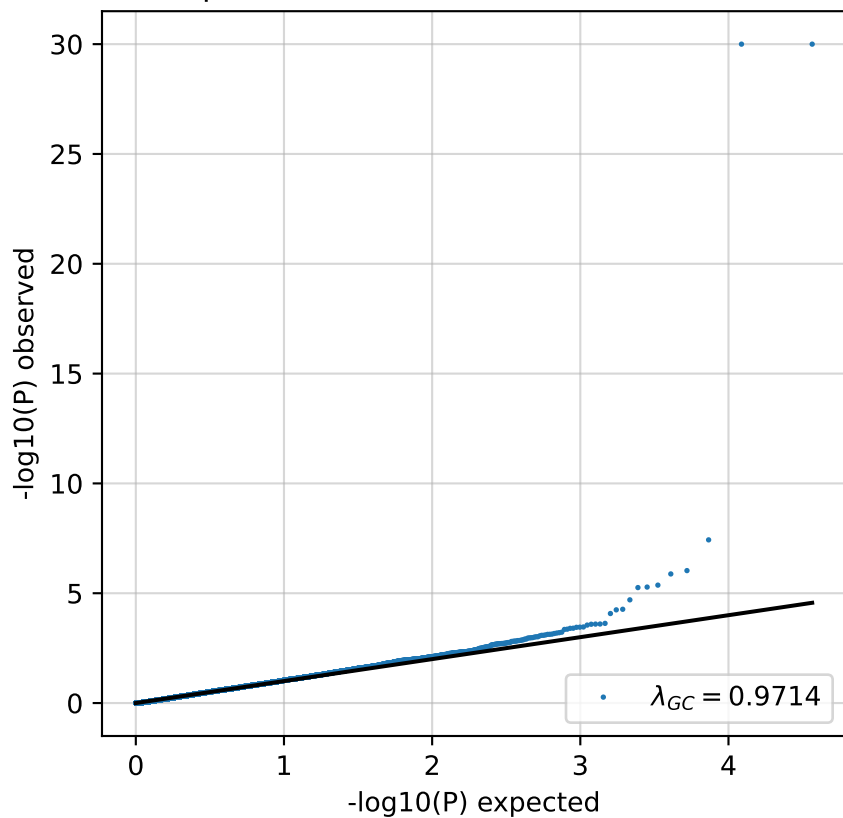

Supplement: Supplementary file 8 — Supplementary Data 5 [file 41467_2022_32864_MOESM8_ESM.zip › qqplots/miss_gbvc_sLRT_LDL_direct.pdf]

Lipoprotein\_A, test type: gbvc,  
implementation: sLRT, var. effect: miss

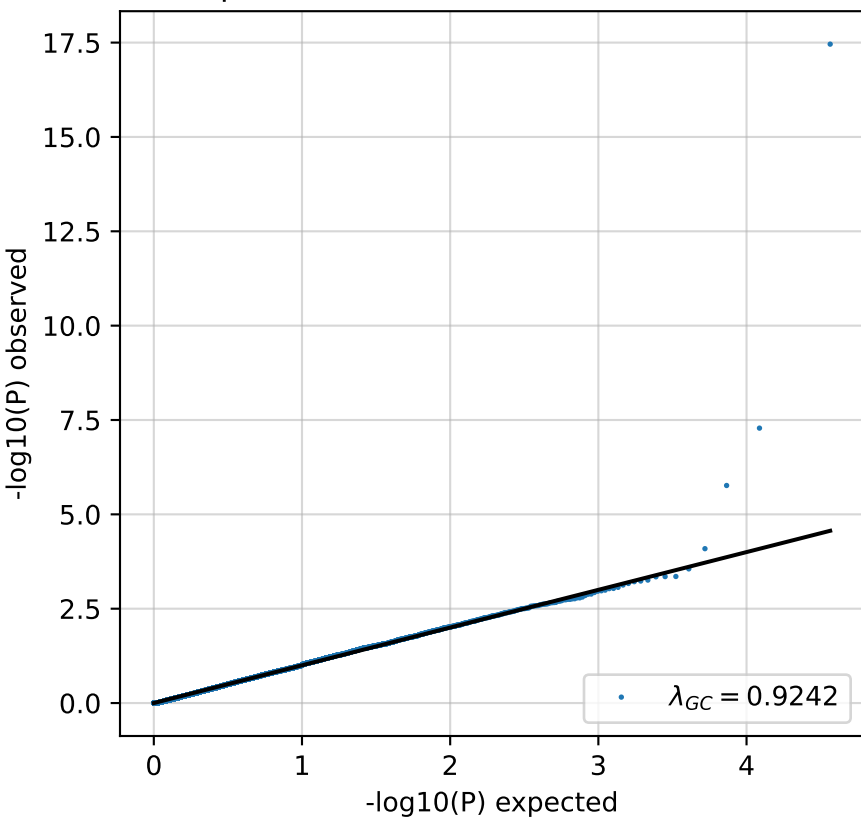

Supplement: Supplementary file 8 — Supplementary Data 5 [file 41467_2022_32864_MOESM8_ESM.zip › qqplots/miss_gbvc_sLRT_Lipoprotein_A.pdf]

Phosphate, test type: gbvc,  
implementation: sLRT, var. effect: miss

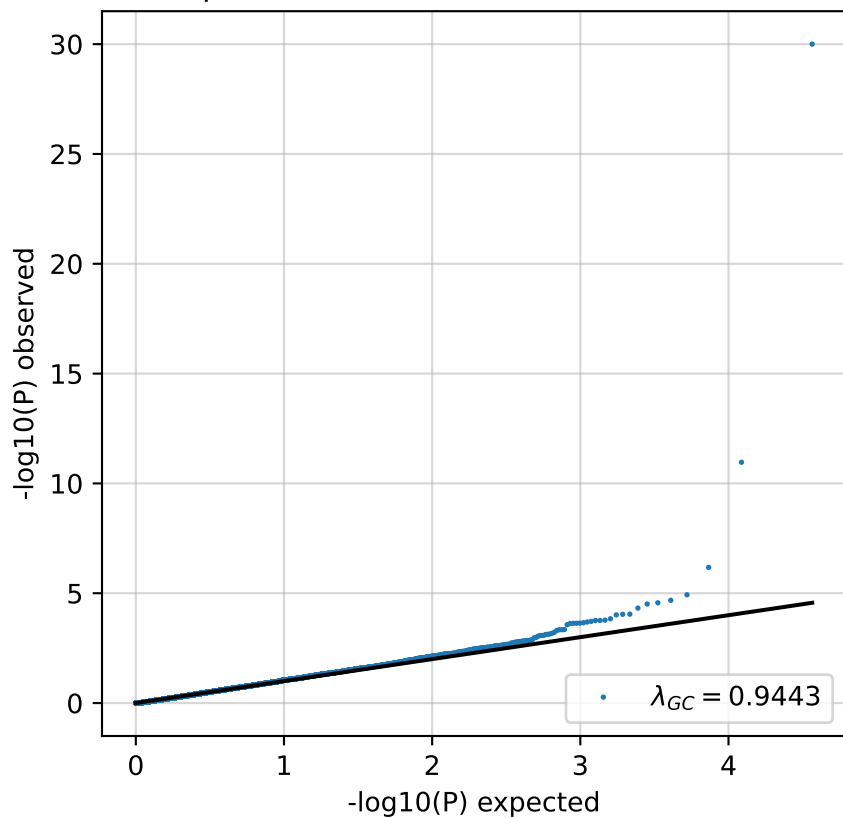

Supplement: Supplementary file 8 — Supplementary Data 5 [file 41467_2022_32864_MOESM8_ESM.zip › qqplots/miss_gbvc_sLRT_Phosphate.pdf]

SHBG, test type: gbvc,  
implementation: sLRT, var. effect: miss

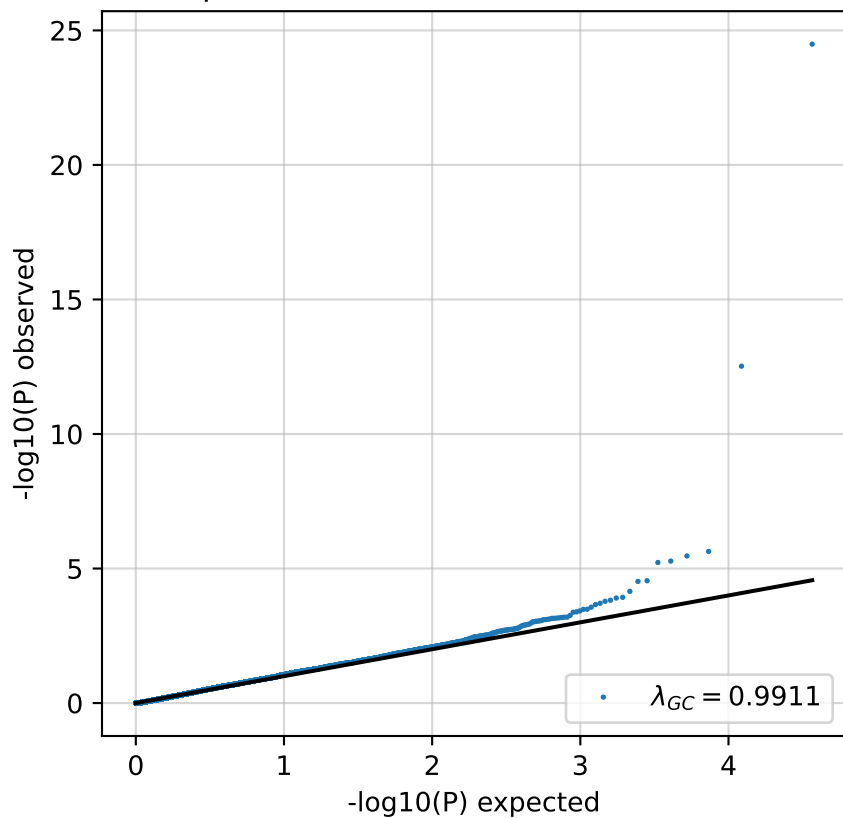

Supplement: Supplementary file 8 — Supplementary Data 5 [file 41467_2022_32864_MOESM8_ESM.zip › qqplots/miss_gbvc_sLRT_SHBG.pdf]

Total\_bilirubin, test type: gbvc,  
implementation: sLRT, var. effect: miss

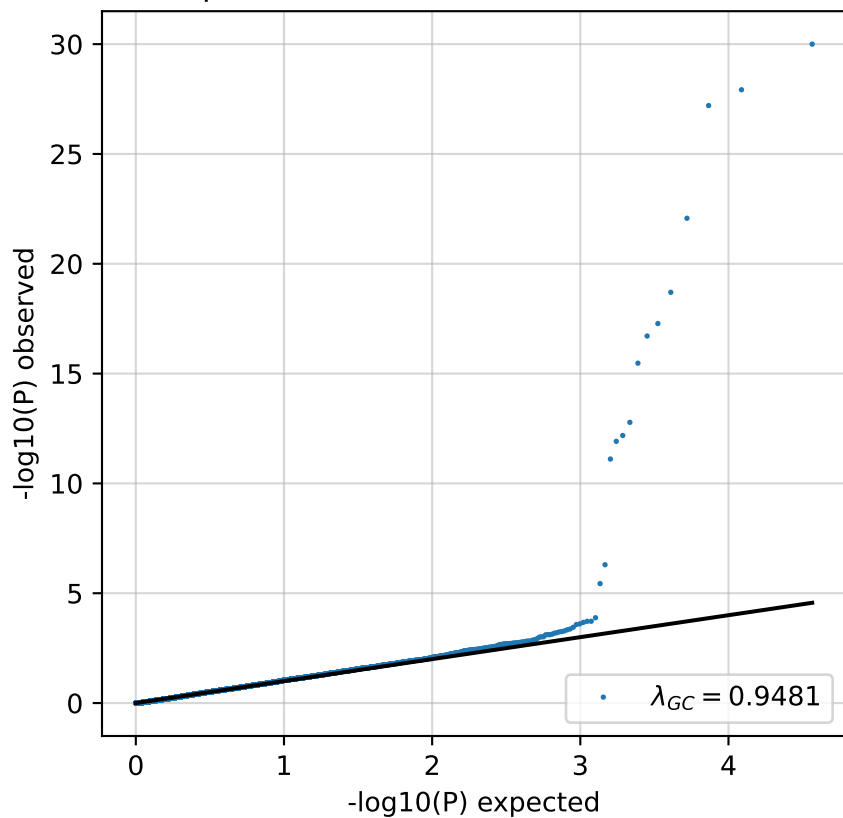

Supplement: Supplementary file 8 — Supplementary Data 5 [file 41467_2022_32864_MOESM8_ESM.zip › qqplots/miss_gbvc_sLRT_Total_bilirubin.pdf]

Total\_protein, test type: gbvc,  
implementation: sLRT, var. effect: miss

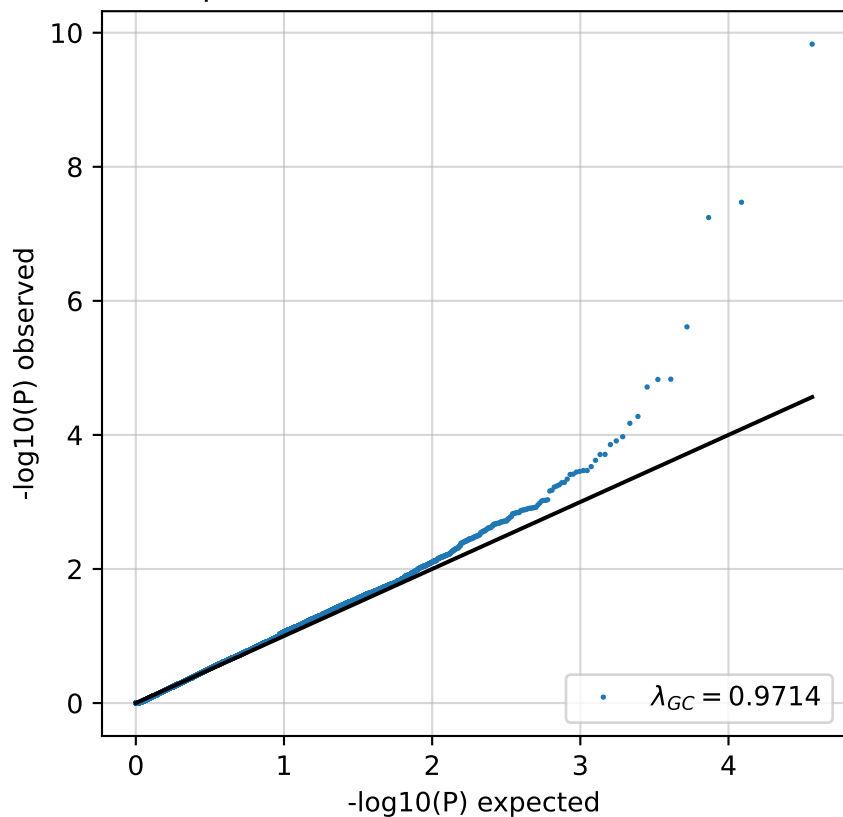

Supplement: Supplementary file 8 — Supplementary Data 5 [file 41467_2022_32864_MOESM8_ESM.zip › qqplots/miss_gbvc_sLRT_Total_protein.pdf]

Triglycerides, test type: gbvc,  
implementation: sLRT, var. effect: miss

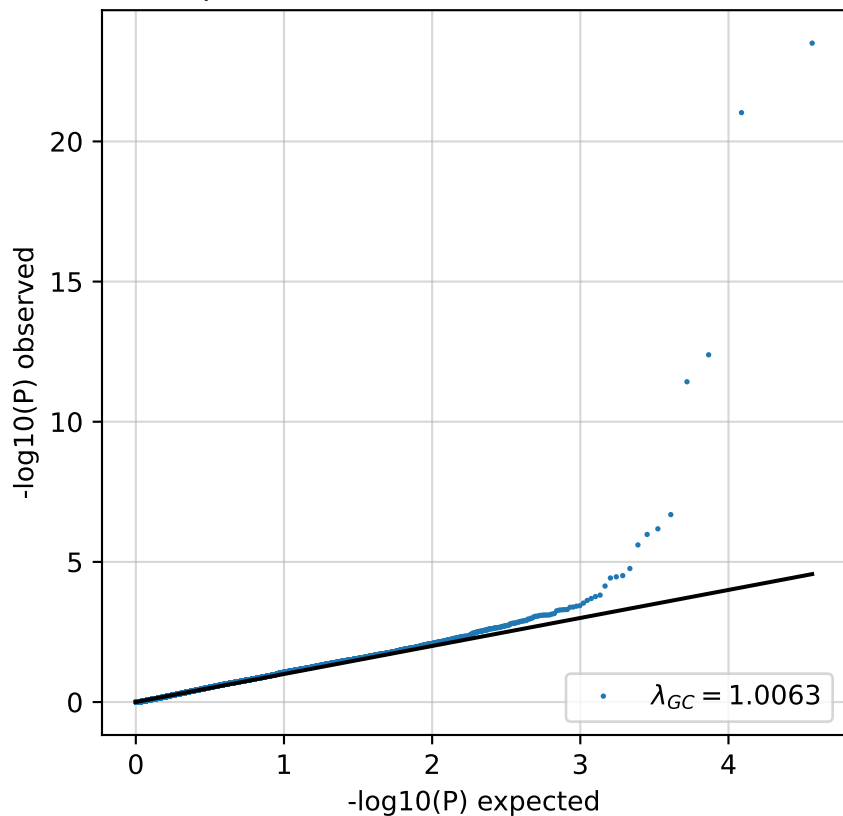

Supplement: Supplementary file 8 — Supplementary Data 5 [file 41467_2022_32864_MOESM8_ESM.zip › qqplots/miss_gbvc_sLRT_Triglycerides.pdf]

Urate, test type: gbvc,  
implementation: sLRT, var. effect: miss

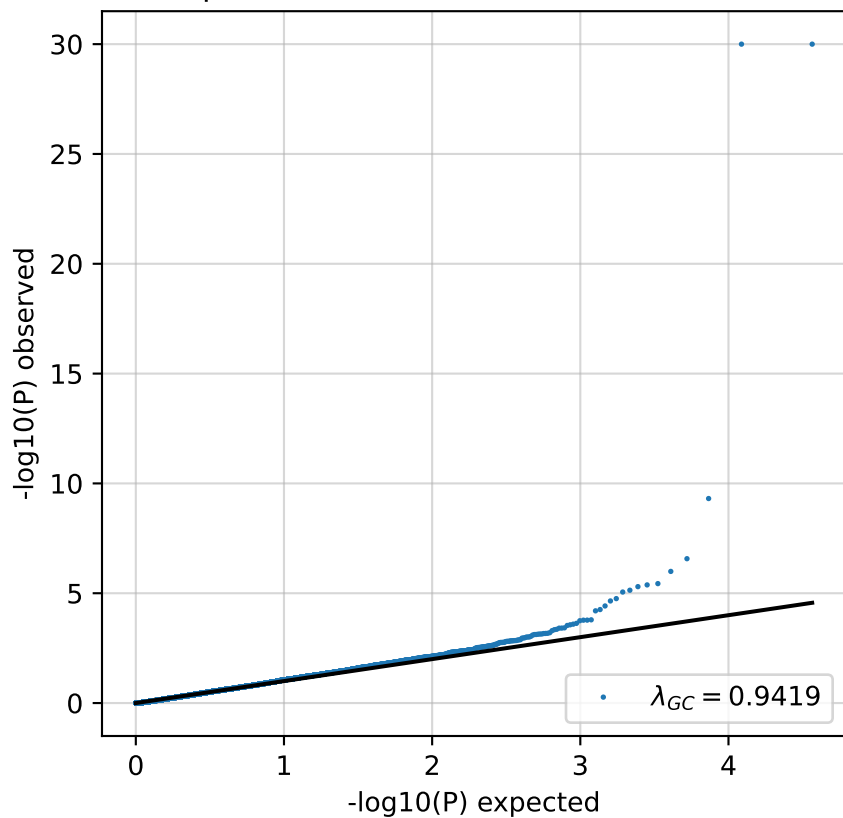

Supplement: Supplementary file 8 — Supplementary Data 5 [file 41467_2022_32864_MOESM8_ESM.zip › qqplots/miss_gbvc_sLRT_Urate.pdf]

Vitamin\_D, test type: gbvc,  
implementation: sLRT, var. effect: miss

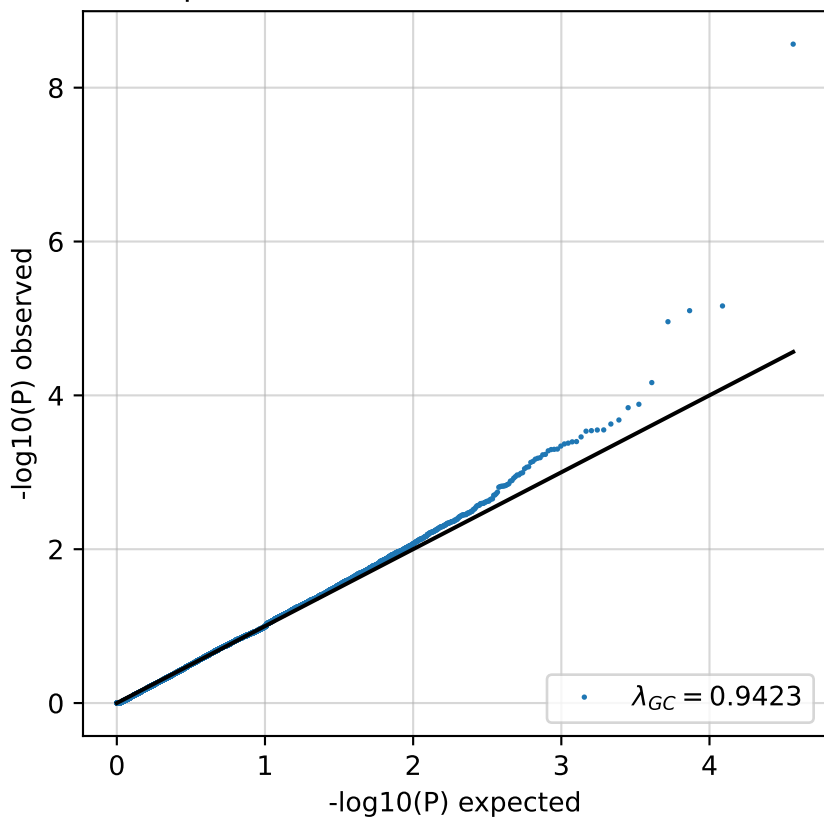

Supplement: Supplementary file 8 — Supplementary Data 5 [file 41467_2022_32864_MOESM8_ESM.zip › qqplots/miss_gbvc_sLRT_Vitamin_D.pdf]

Alanine\_aminotransferase, test type: gbvc,  
implementation: score, var. effect: pLOF

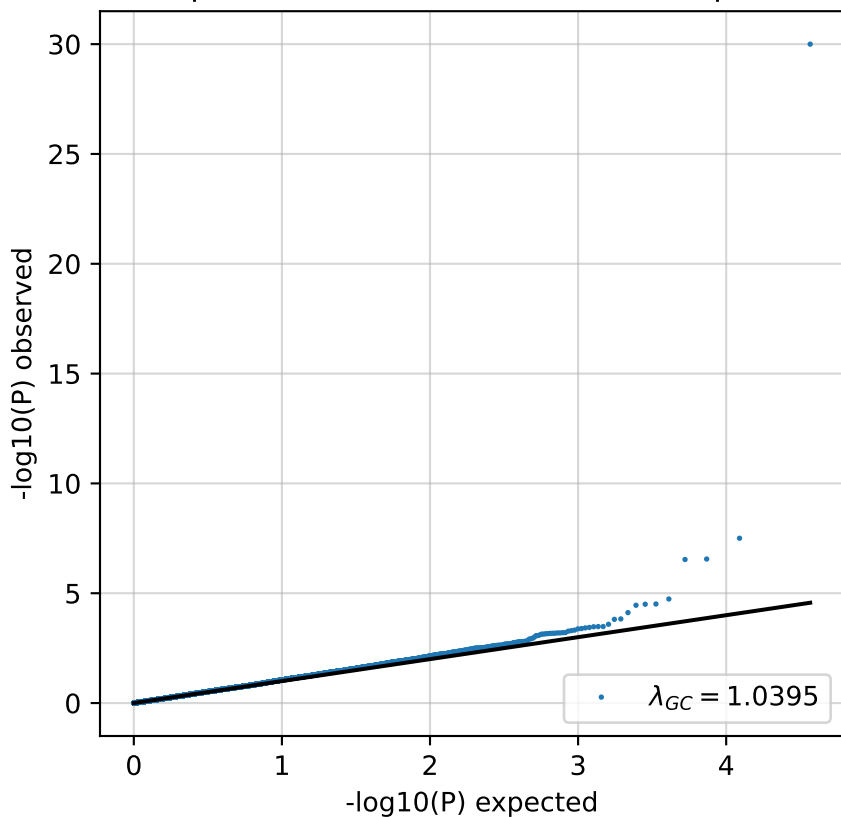

Supplement: Supplementary file 8 — Supplementary Data 5 [file 41467_2022_32864_MOESM8_ESM.zip › qqplots/pLOF_gbvc_score_Alanine_aminotransferase.pdf]

Albumin, test type: gbvc,  
implementation: score, var. effect: pLOF

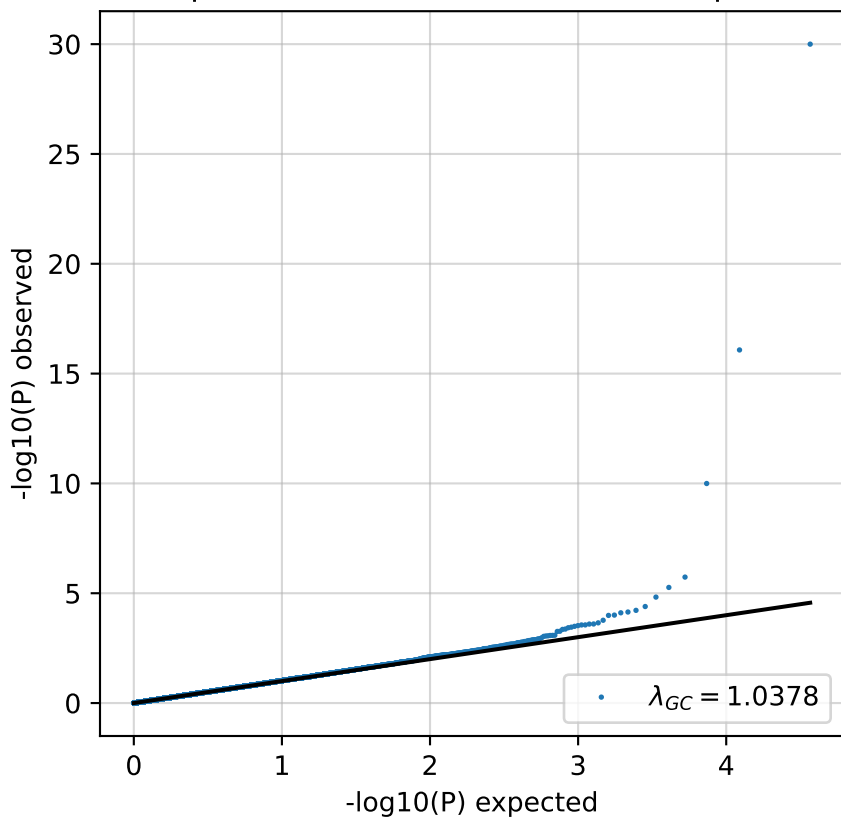

Supplement: Supplementary file 8 — Supplementary Data 5 [file 41467_2022_32864_MOESM8_ESM.zip › qqplots/pLOF_gbvc_score_Albumin.pdf]

Alkaline\_phosphatase, test type: gbvc,  
implementation: score, var. effect: pLOF

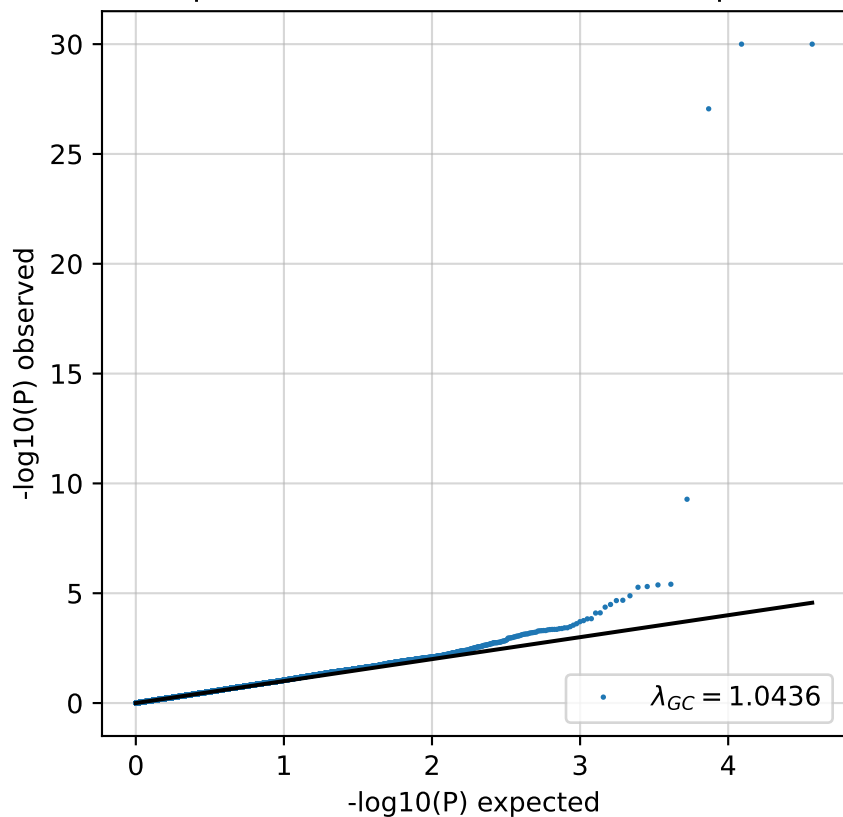

Supplement: Supplementary file 8 — Supplementary Data 5 [file 41467_2022_32864_MOESM8_ESM.zip › qqplots/pLOF_gbvc_score_Alkaline_phosphatase.pdf]

Apolipoprotein\_A, test type: gbvc,  
implementation: score, var. effect: pLOF

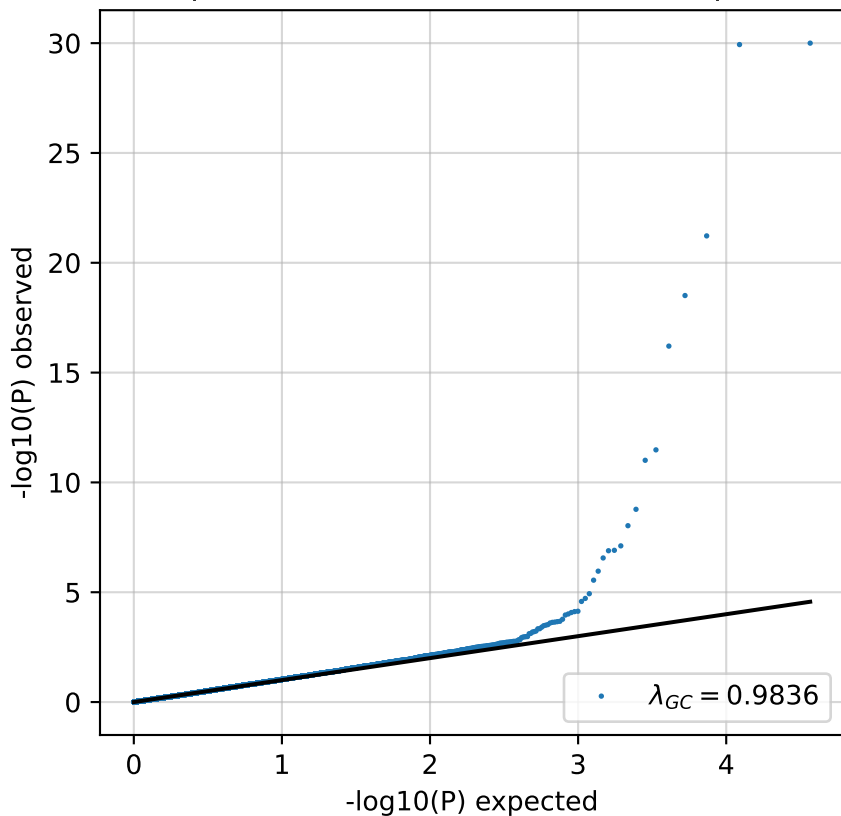

Supplement: Supplementary file 8 — Supplementary Data 5 [file 41467_2022_32864_MOESM8_ESM.zip › qqplots/pLOF_gbvc_score_Apolipoprotein_A.pdf]

Apolipoprotein\_B, test type: gbvc,  
implementation: score, var. effect: pLOF

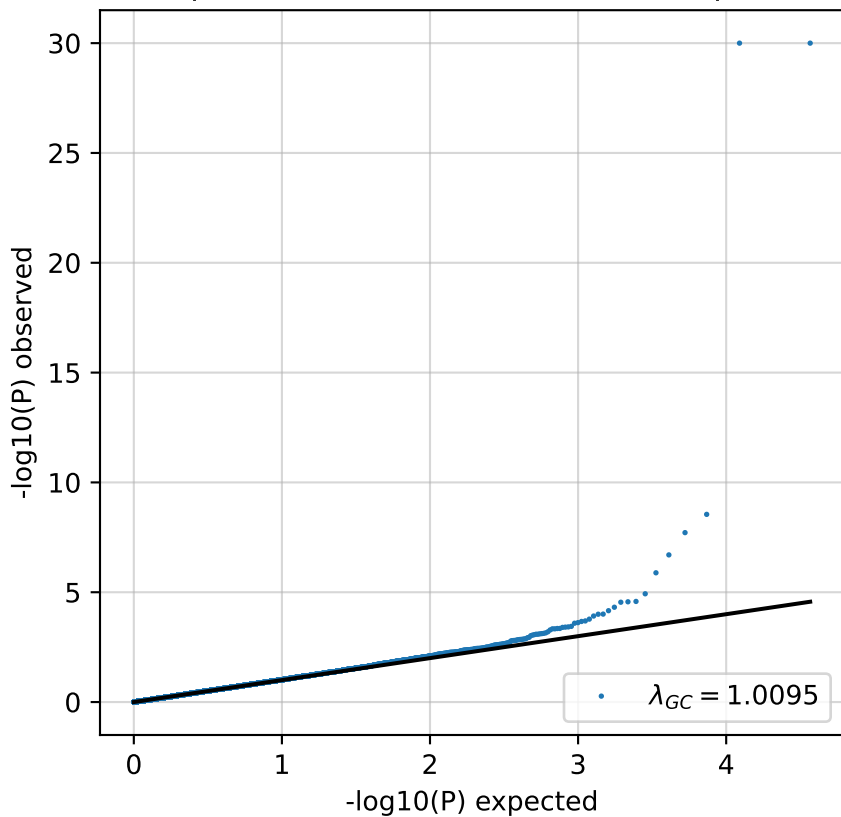

Supplement: Supplementary file 8 — Supplementary Data 5 [file 41467_2022_32864_MOESM8_ESM.zip › qqplots/pLOF_gbvc_score_Apolipoprotein_B.pdf]

Aspartate\_aminotransferase, test type: gbvc,  
implementation: score, var. effect: pLOF

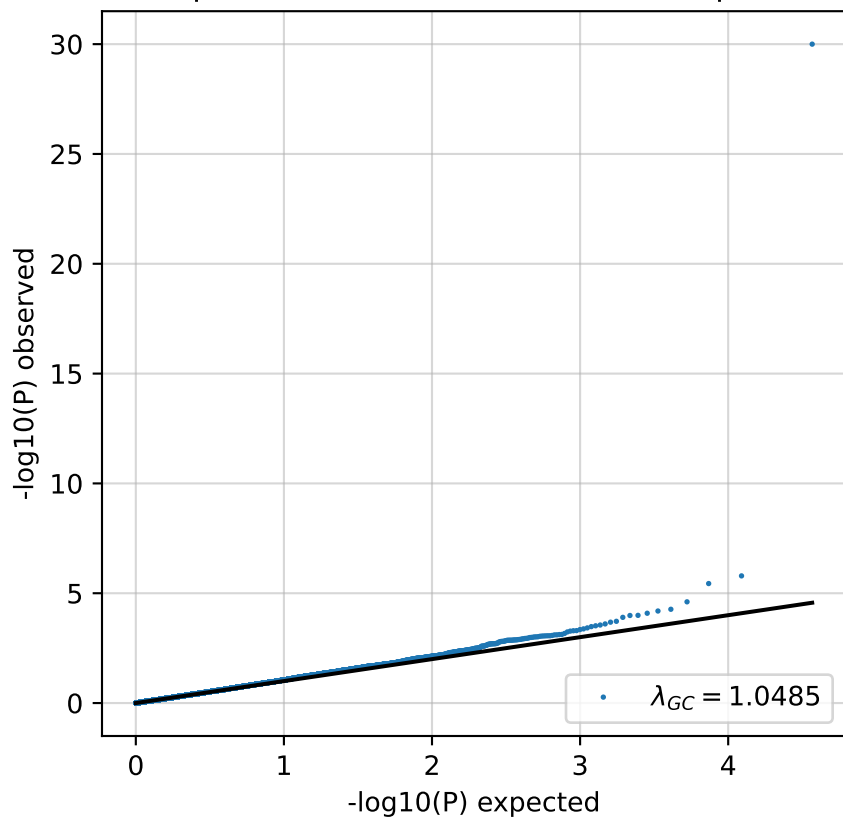

Supplement: Supplementary file 8 — Supplementary Data 5 [file 41467_2022_32864_MOESM8_ESM.zip › qqplots/pLOF_gbvc_score_Aspartate_aminotransferase.pdf]

Calcium, test type: gbvc,  
implementation: score, var. effect: pLOF

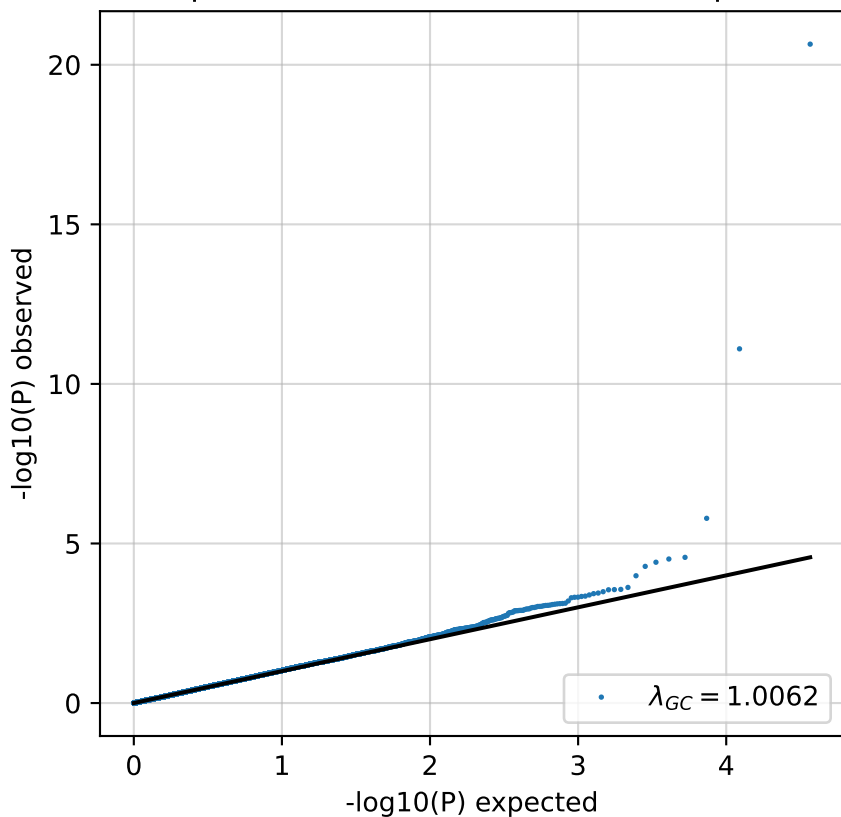

Supplement: Supplementary file 8 — Supplementary Data 5 [file 41467_2022_32864_MOESM8_ESM.zip › qqplots/pLOF_gbvc_score_Calcium.pdf]

Cholesterol, test type: gbvc,  
implementation: score, var. effect: pLOF

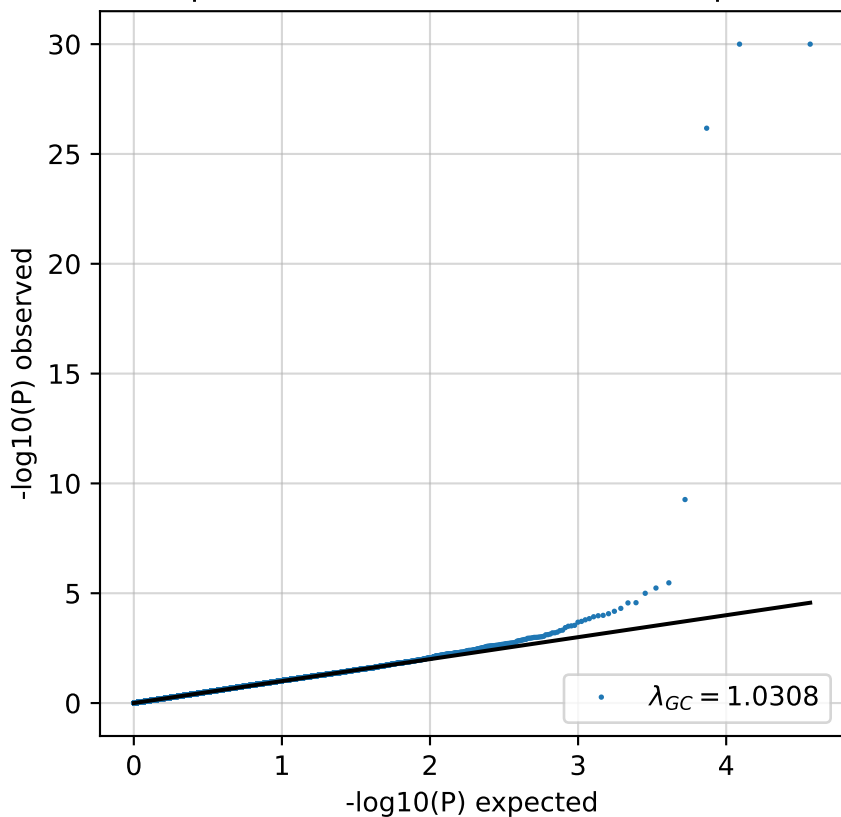

Supplement: Supplementary file 8 — Supplementary Data 5 [file 41467_2022_32864_MOESM8_ESM.zip › qqplots/pLOF_gbvc_score_Cholesterol.pdf]

C-reactive\_protein, test type: gbvc,  
implementation: score, var. effect: pLOF

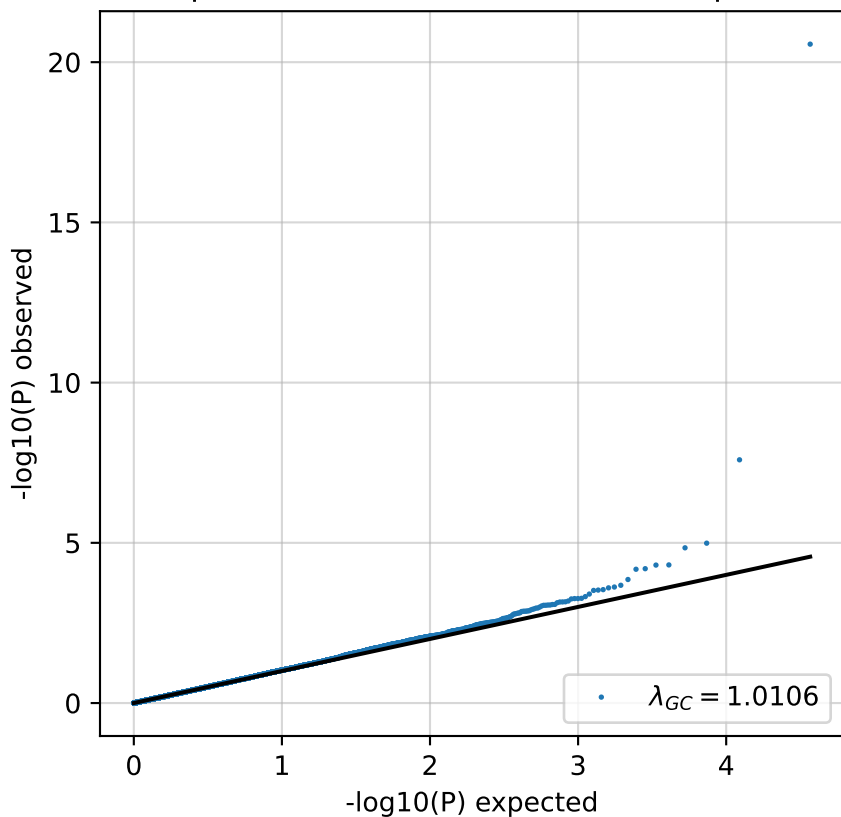

Supplement: Supplementary file 8 — Supplementary Data 5 [file 41467_2022_32864_MOESM8_ESM.zip › qqplots/pLOF_gbvc_score_Creactive_protein.pdf]

Creatinine, test type: gbvc,  
implementation: score, var. effect: pLOF

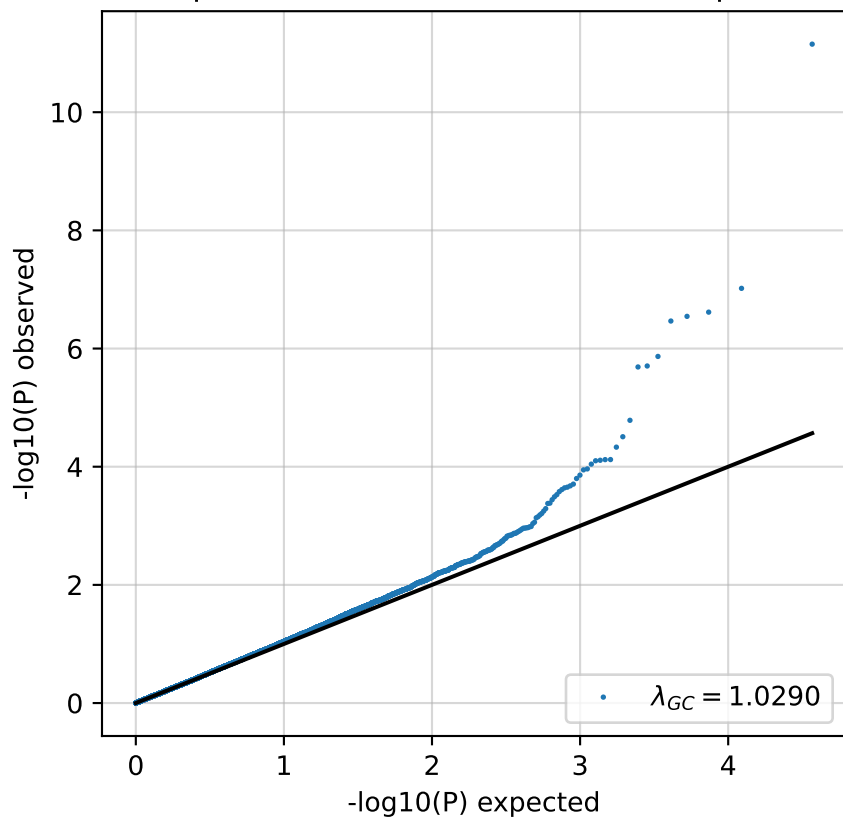

Supplement: Supplementary file 8 — Supplementary Data 5 [file 41467_2022_32864_MOESM8_ESM.zip › qqplots/pLOF_gbvc_score_Creatinine.pdf]

Cystatin\_C, test type: gbvc,  
implementation: score, var. effect: pLOF

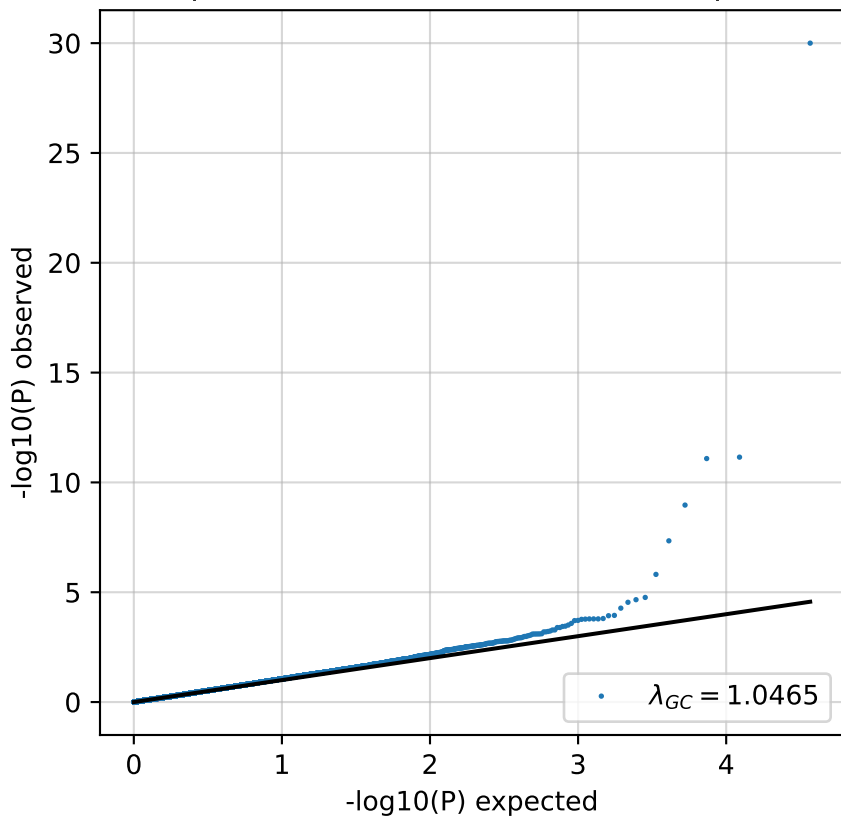

Supplement: Supplementary file 8 — Supplementary Data 5 [file 41467_2022_32864_MOESM8_ESM.zip › qqplots/pLOF_gbvc_score_Cystatin_C.pdf]

Direct\_bilirubin, test type: gbvc,  
implementation: score, var. effect: pLOF

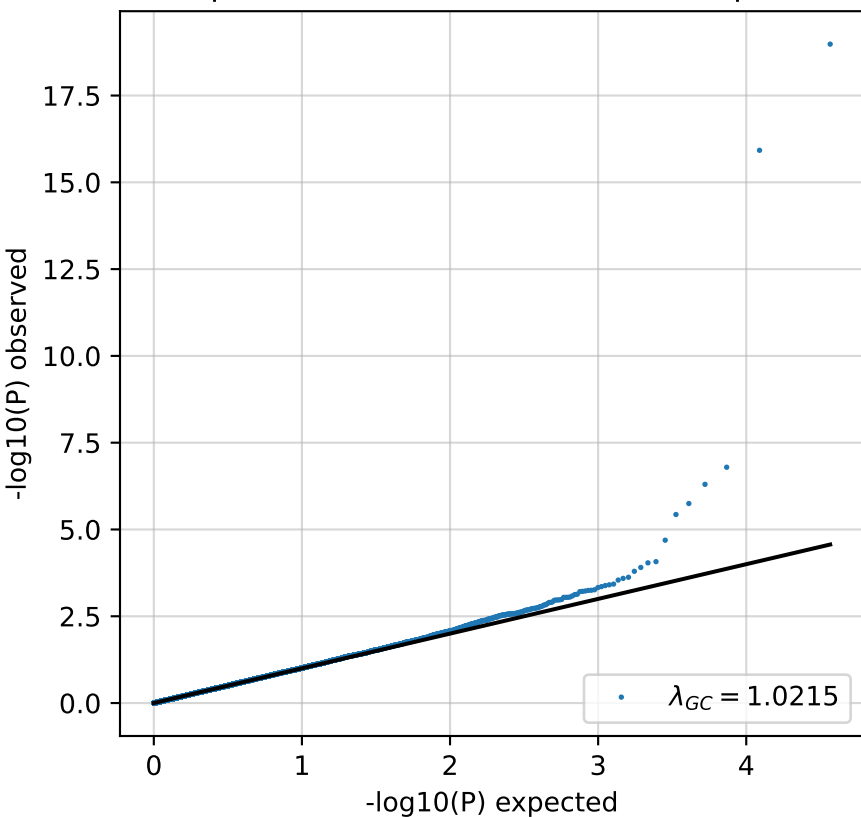

Supplement: Supplementary file 8 — Supplementary Data 5 [file 41467_2022_32864_MOESM8_ESM.zip › qqplots/pLOF_gbvc_score_Direct_bilirubin.pdf]

Glucose, test type: gbvc,  
implementation: score, var. effect: pLOF

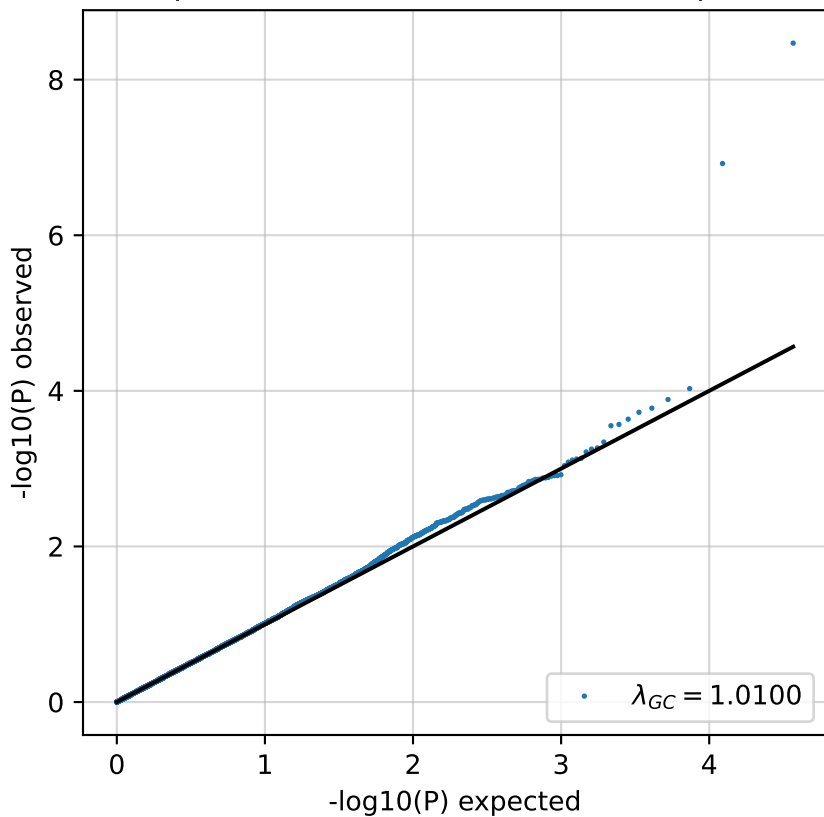

Supplement: Supplementary file 8 — Supplementary Data 5 [file 41467_2022_32864_MOESM8_ESM.zip › qqplots/pLOF_gbvc_score_Glucose.pdf]

Glycated\_haemoglobin\_(HbA1c), test type: gbvc,  
implementation: score, var. effect: pLOF

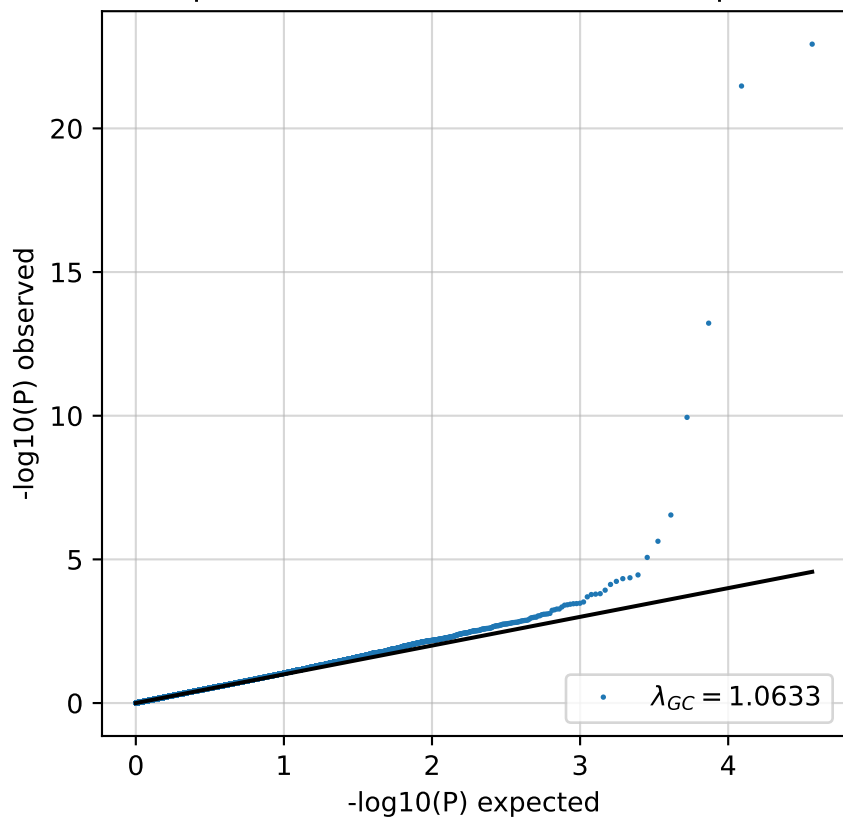

Supplement: Supplementary file 8 — Supplementary Data 5 [file 41467_2022_32864_MOESM8_ESM.zip › qqplots/pLOF_gbvc_score_Glycated_haemoglobin_HbA1c.pdf]

HDL\_cholesterol, test type: gbvc,  
implementation: score, var. effect: pLOF

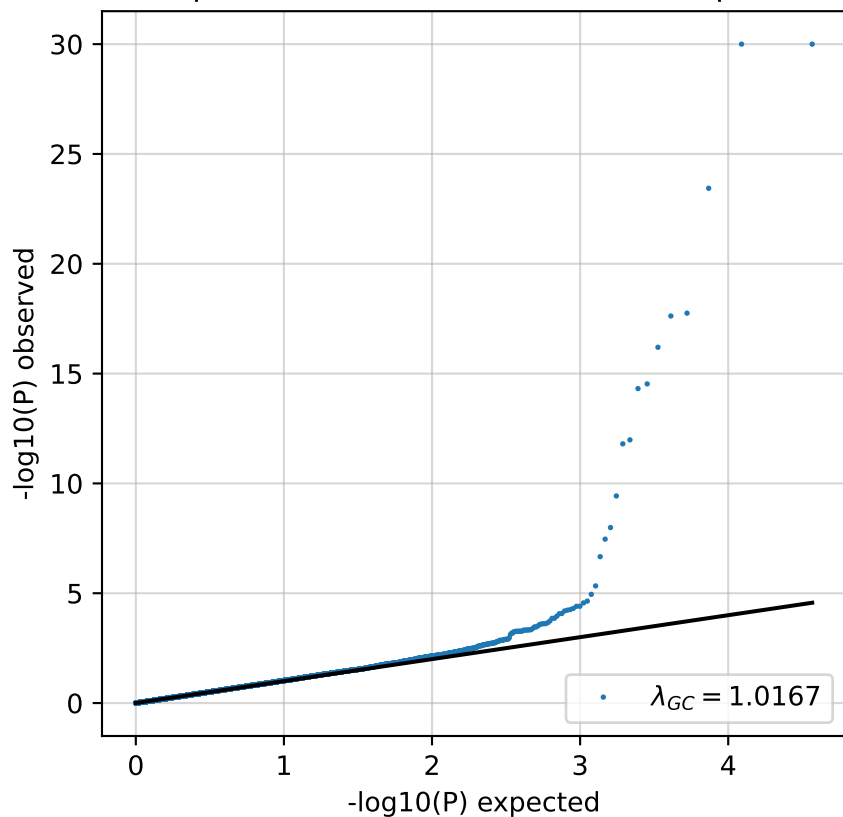

Supplement: Supplementary file 8 — Supplementary Data 5 [file 41467_2022_32864_MOESM8_ESM.zip › qqplots/pLOF_gbvc_score_HDL_cholesterol.pdf]

LDL\_direct, test type: gbvc,  
implementation: score, var. effect: pLOF

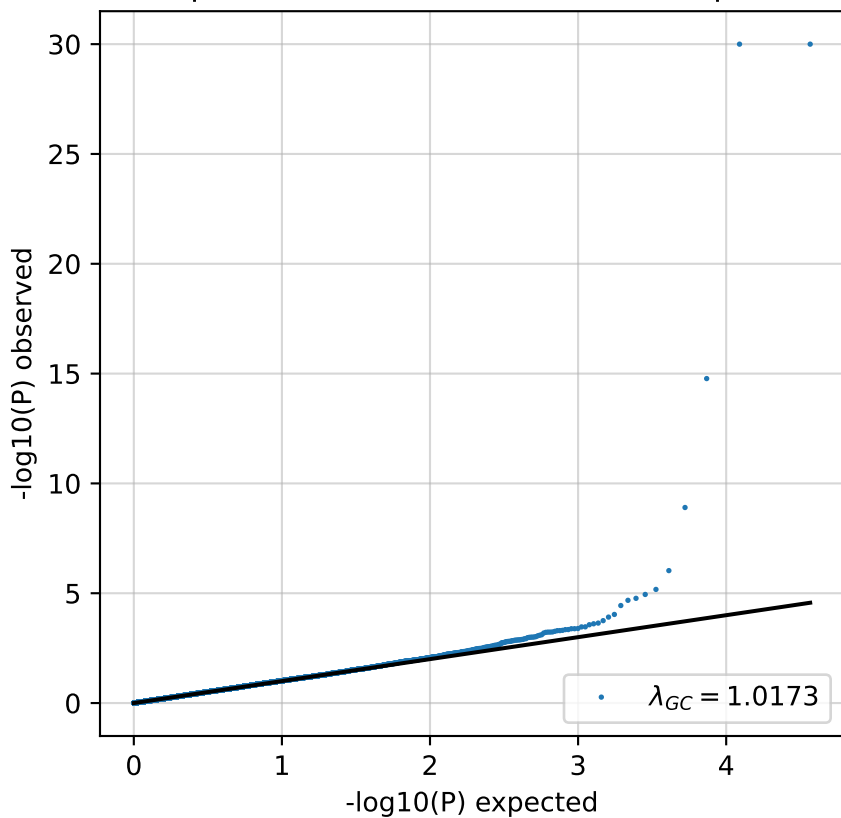

Supplement: Supplementary file 8 — Supplementary Data 5 [file 41467_2022_32864_MOESM8_ESM.zip › qqplots/pLOF_gbvc_score_LDL_direct.pdf]

Phosphate, test type: gbvc,  
implementation: score, var. effect: pLOF

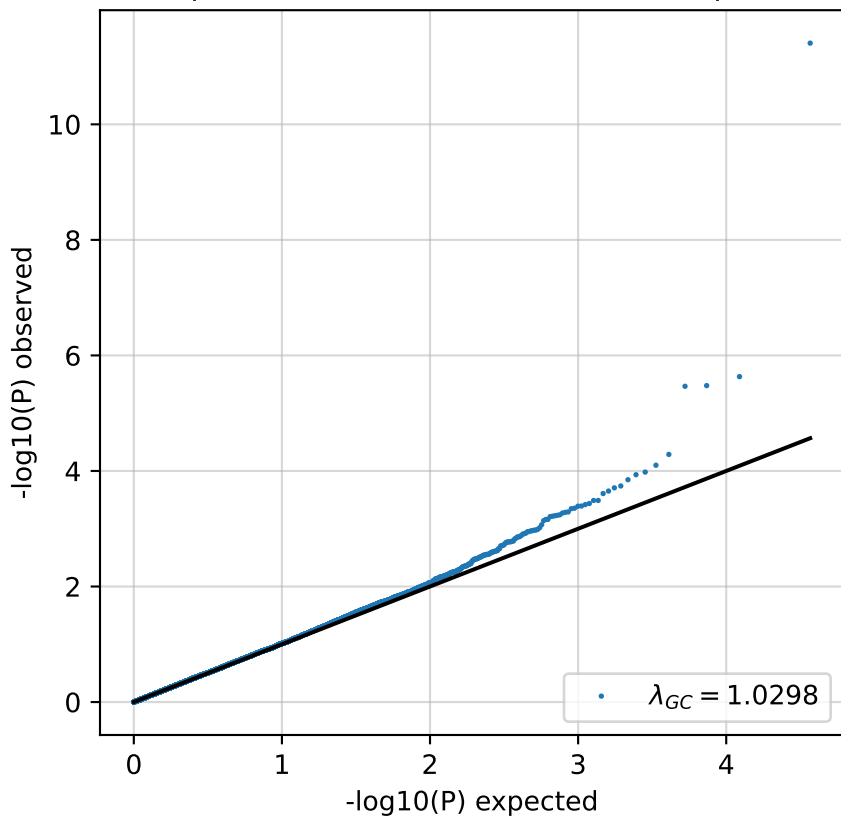

Supplement: Supplementary file 8 — Supplementary Data 5 [file 41467_2022_32864_MOESM8_ESM.zip › qqplots/pLOF_gbvc_score_Phosphate.pdf]

SHBG, test type: gbvc,  
implementation: score, var. effect: pLOF

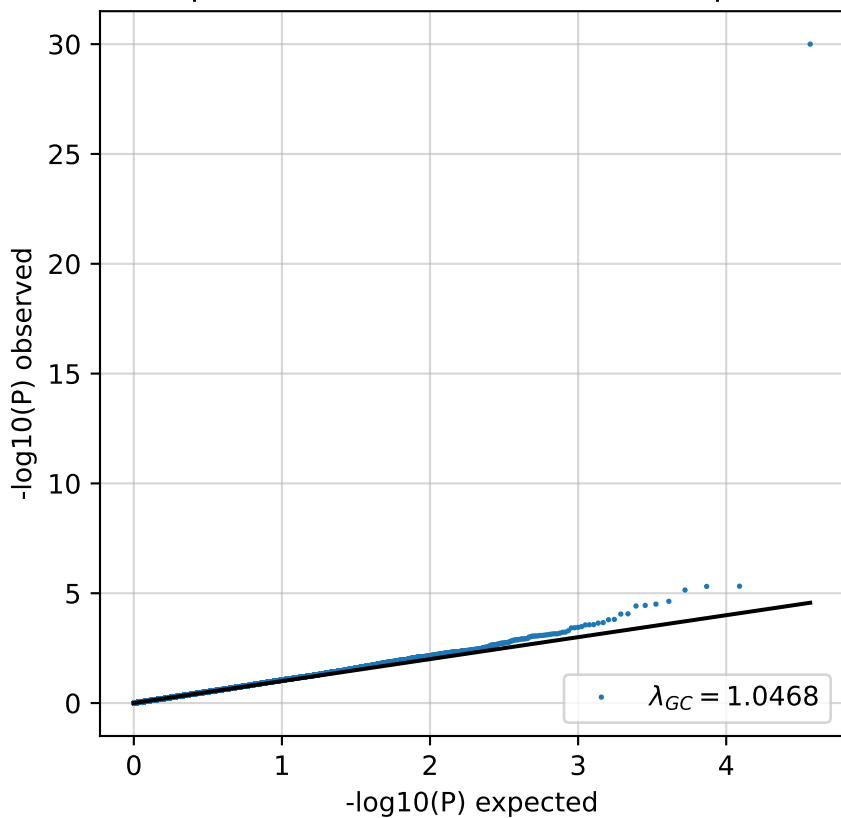

Supplement: Supplementary file 8 — Supplementary Data 5 [file 41467_2022_32864_MOESM8_ESM.zip › qqplots/pLOF_gbvc_score_SHBG.pdf]

Testosterone, test type: gbvc,  
implementation: score, var. effect: pLOF

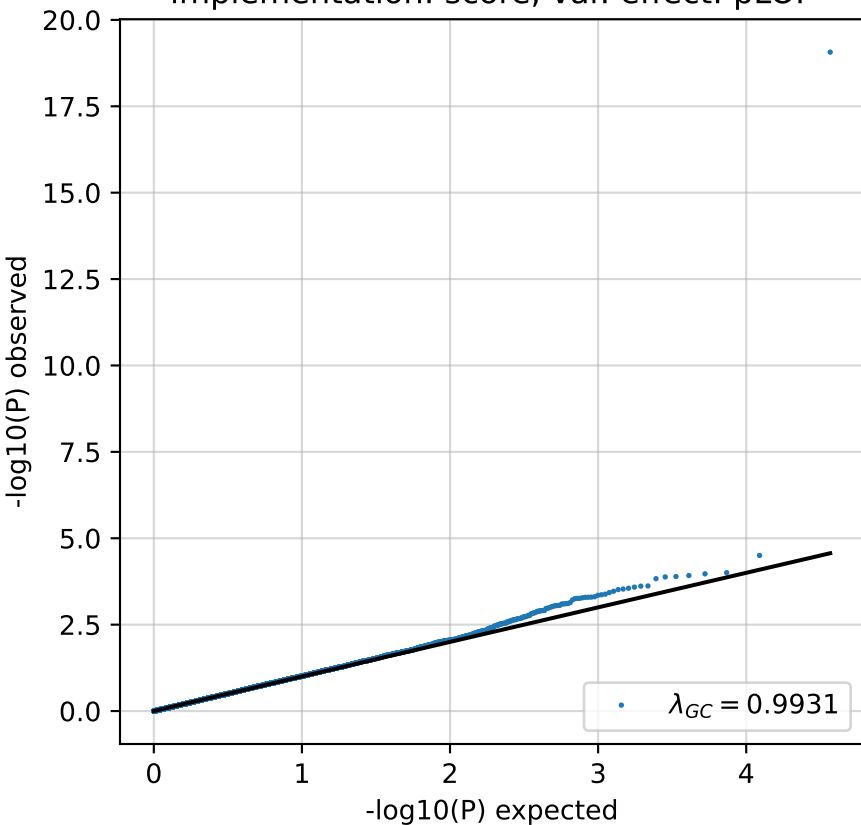

Supplement: Supplementary file 8 — Supplementary Data 5 [file 41467_2022_32864_MOESM8_ESM.zip › qqplots/pLOF_gbvc_score_Testosterone.pdf]

Total\_bilirubin, test type: gbvc,  
implementation: score, var. effect: pLOF

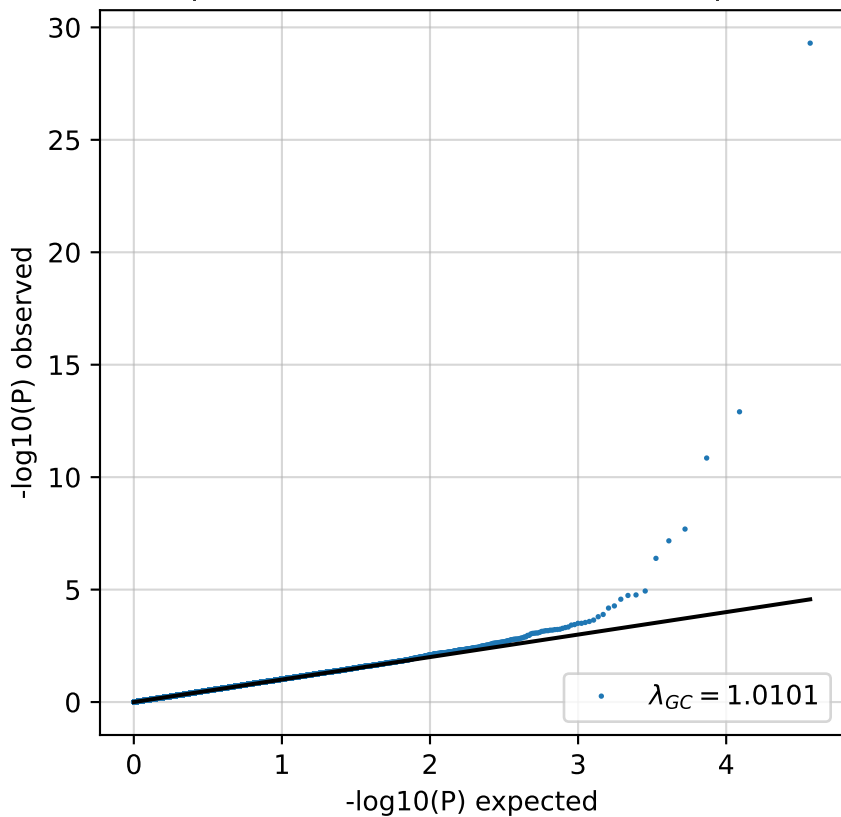

Supplement: Supplementary file 8 — Supplementary Data 5 [file 41467_2022_32864_MOESM8_ESM.zip › qqplots/pLOF_gbvc_score_Total_bilirubin.pdf]

Total\_protein, test type: gbvc,  
implementation: score, var. effect: pLOF

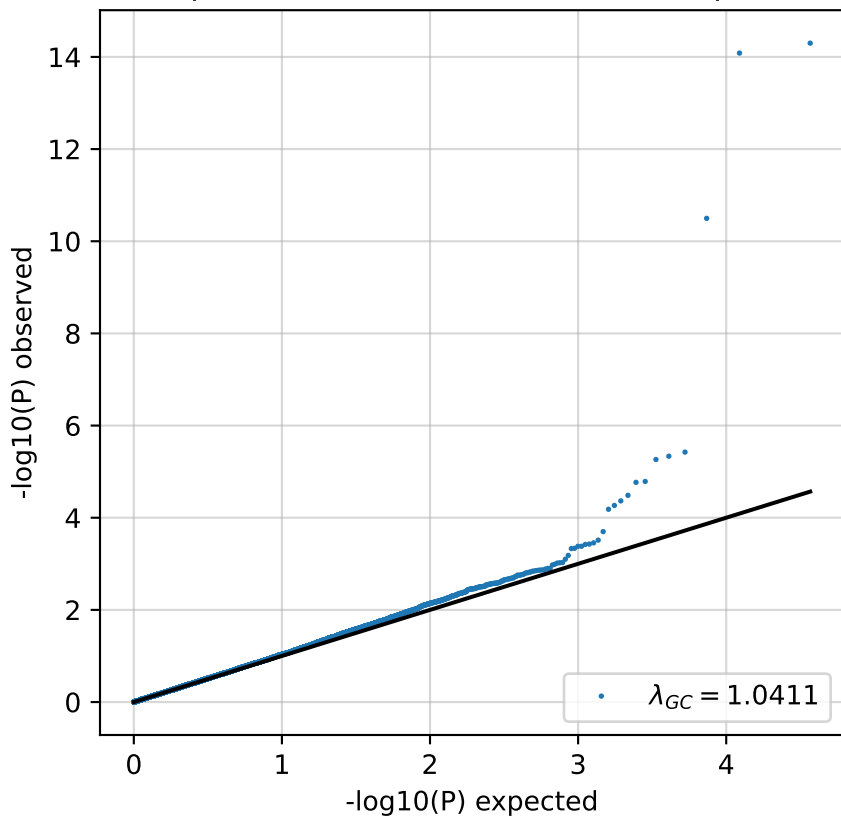

Supplement: Supplementary file 8 — Supplementary Data 5 [file 41467_2022_32864_MOESM8_ESM.zip › qqplots/pLOF_gbvc_score_Total_protein.pdf]

Triglycerides, test type: gbvc,  
implementation: score, var. effect: pLOF

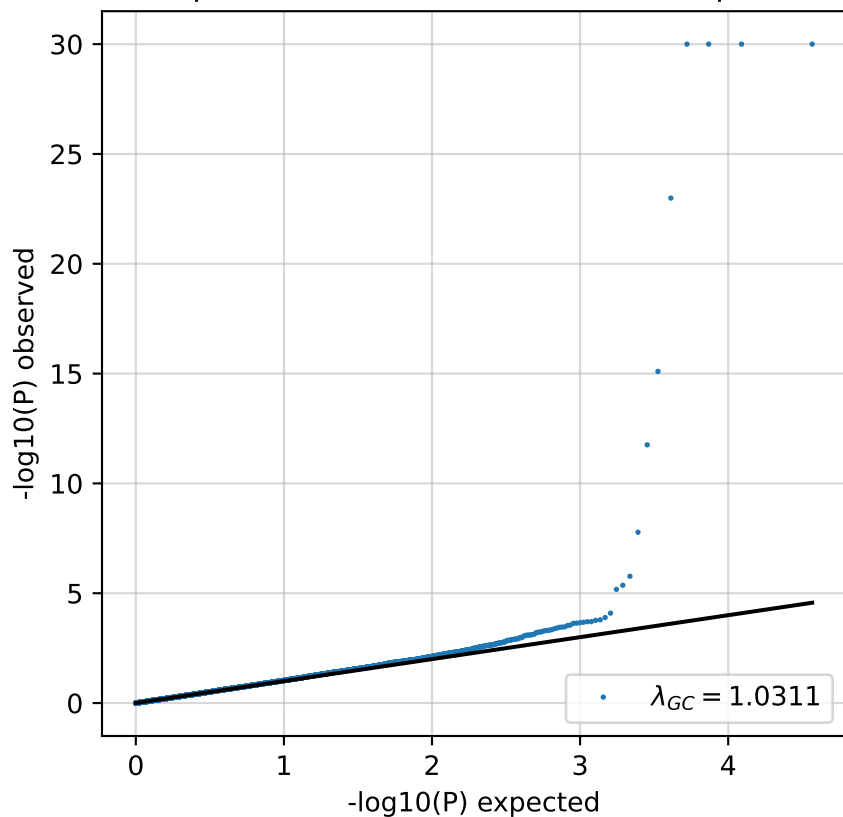

Supplement: Supplementary file 8 — Supplementary Data 5 [file 41467_2022_32864_MOESM8_ESM.zip › qqplots/pLOF_gbvc_score_Triglycerides.pdf]

Urate, test type: gbvc,  
implementation: score, var. effect: pLOF

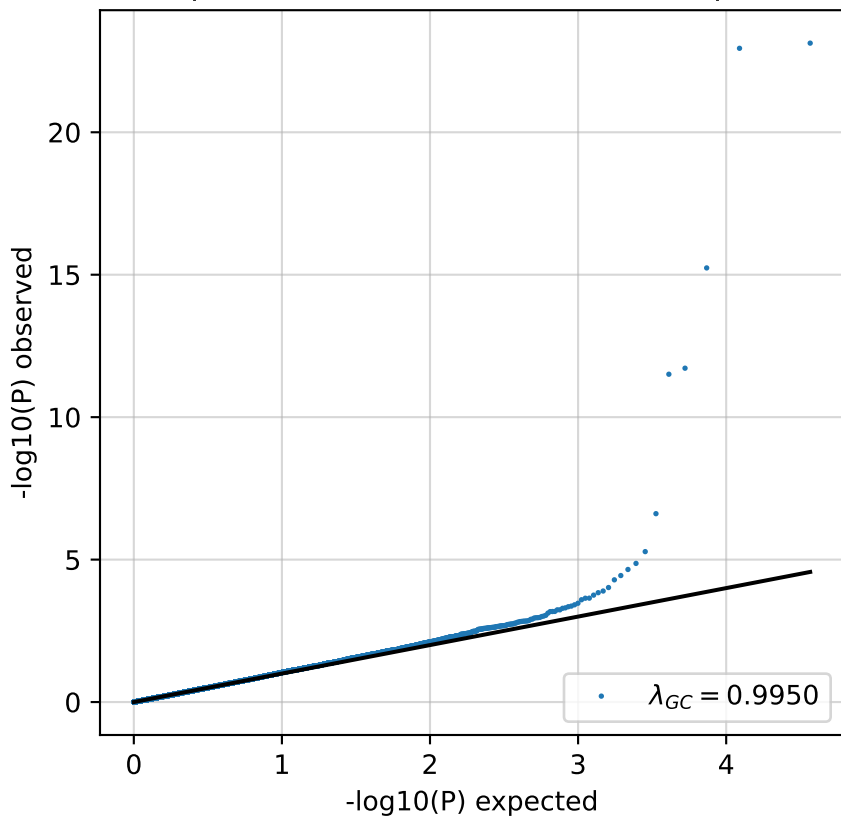

Supplement: Supplementary file 8 — Supplementary Data 5 [file 41467_2022_32864_MOESM8_ESM.zip › qqplots/pLOF_gbvc_score_Urate.pdf]

Vitamin\_D, test type: gbvc,  
implementation: score, var. effect: pLOF

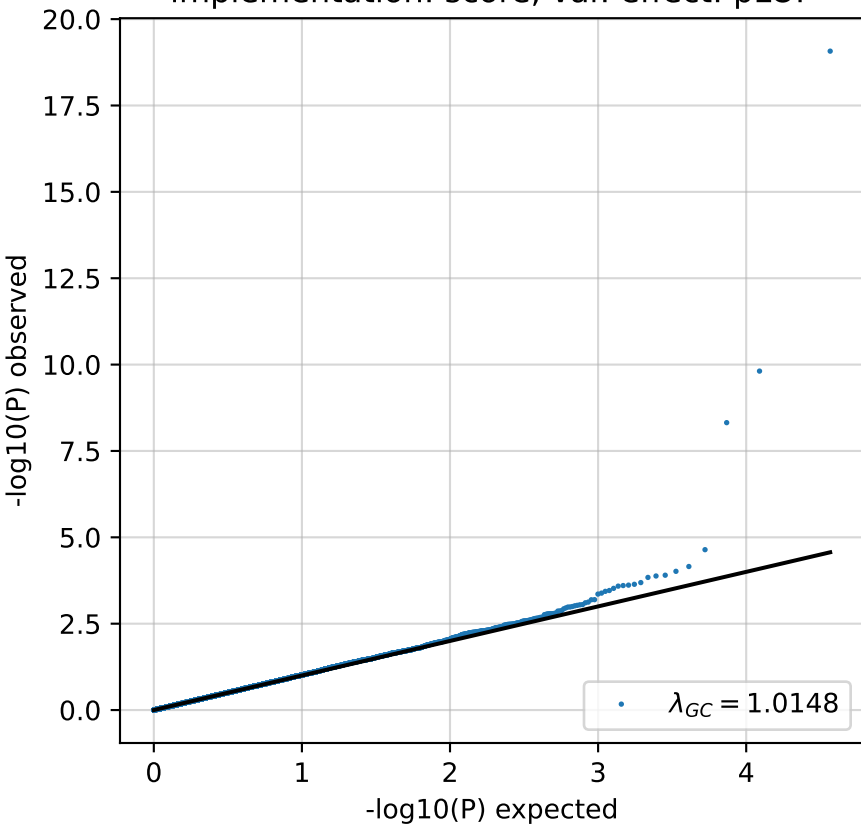

Supplement: Supplementary file 8 — Supplementary Data 5 [file 41467_2022_32864_MOESM8_ESM.zip › qqplots/pLOF_gbvc_score_Vitamin_D.pdf]

Alanine\_aminotransferase, test type: K,  
implementation: sLRT, var. effect: rbp

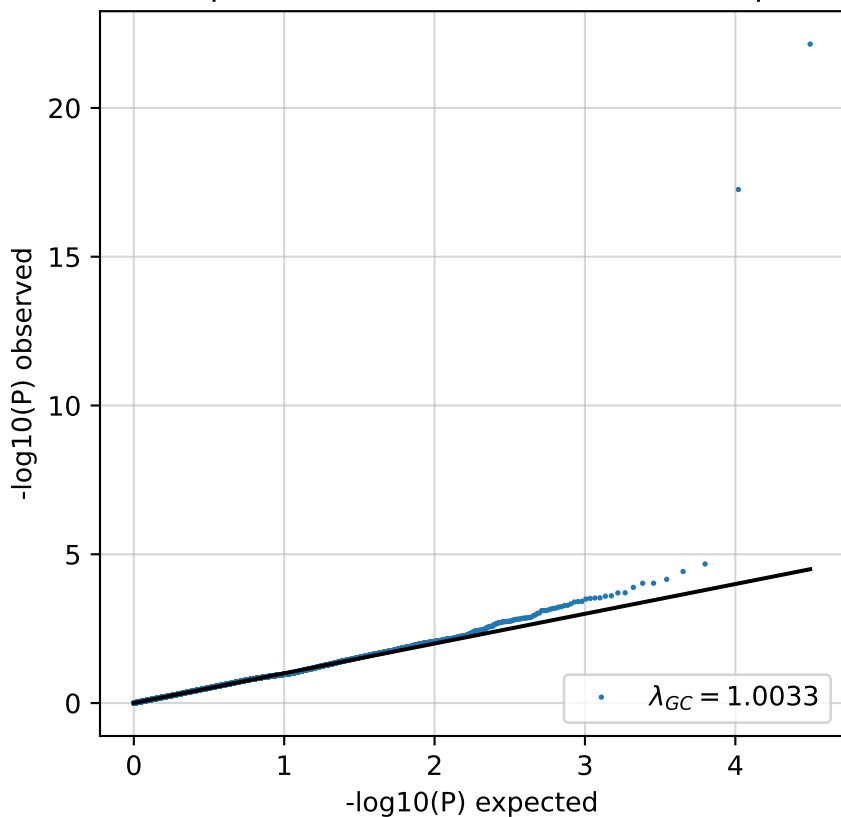

Supplement: Supplementary file 8 — Supplementary Data 5 [file 41467_2022_32864_MOESM8_ESM.zip › qqplots/rbp_K_sLRT_Alanine_aminotransferase.pdf]

Lipoprotein\_A, test type: K,  
implementation: sLRT, var. effect: rbp

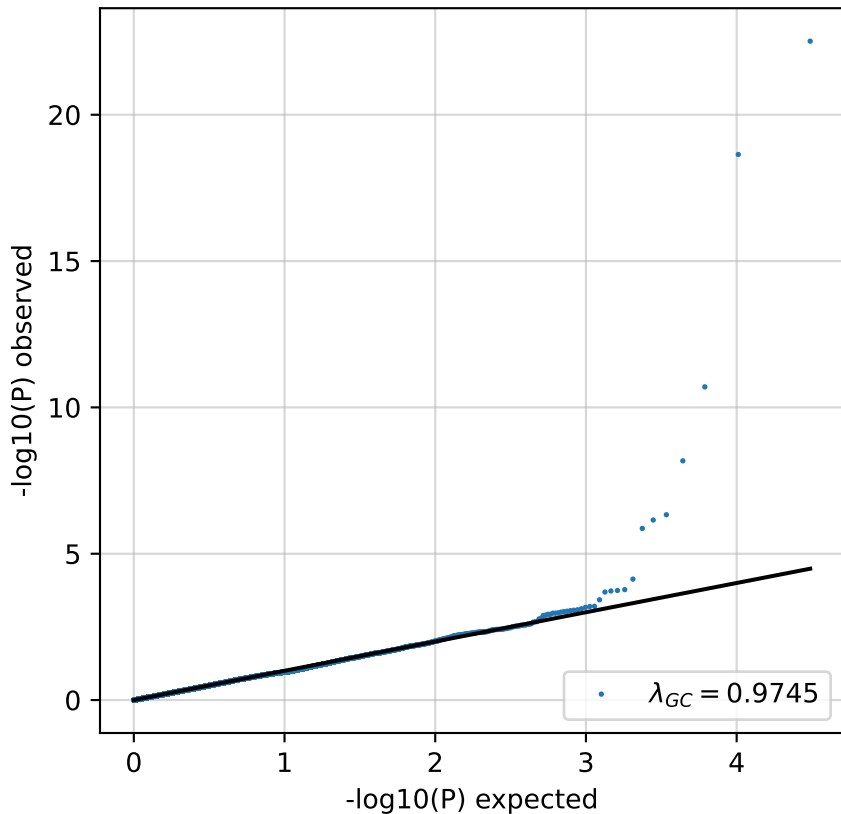

Supplement: Supplementary file 8 — Supplementary Data 5 [file 41467_2022_32864_MOESM8_ESM.zip › qqplots/rbp_K_sLRT_Lipoprotein_A.pdf]

Total\_bilirubin, test type: K,  
implementation: sLRT, var. effect: rbp

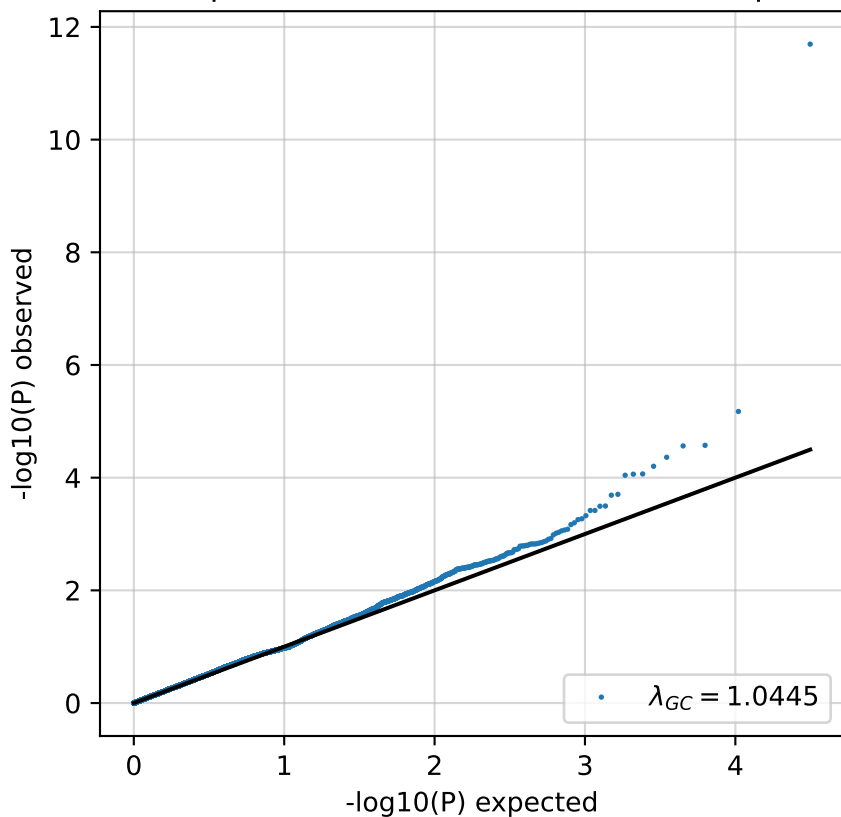

Supplement: Supplementary file 8 — Supplementary Data 5 [file 41467_2022_32864_MOESM8_ESM.zip › qqplots/rbp_K_sLRT_Total_bilirubin.pdf]

Triglycerides, test type: K,  
implementation: sLRT, var. effect: rbp

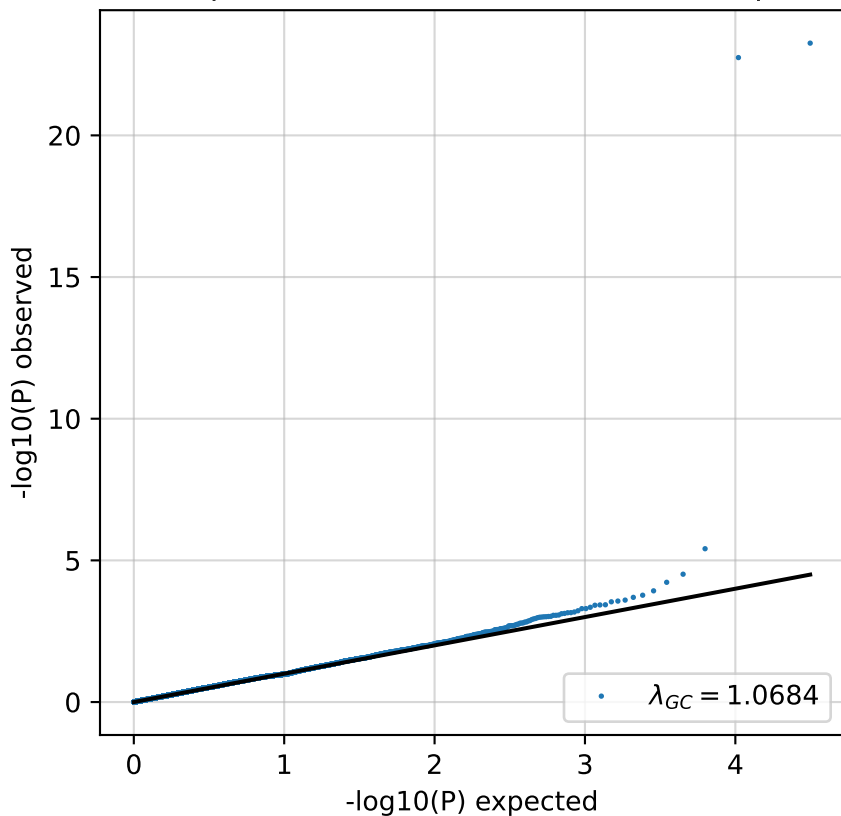

Supplement: Supplementary file 8 — Supplementary Data 5 [file 41467_2022_32864_MOESM8_ESM.zip › qqplots/rbp_K_sLRT_Triglycerides.pdf]

Alanine\_aminotransferase, test type: K,  
implementation: sLRT, var. effect: splice

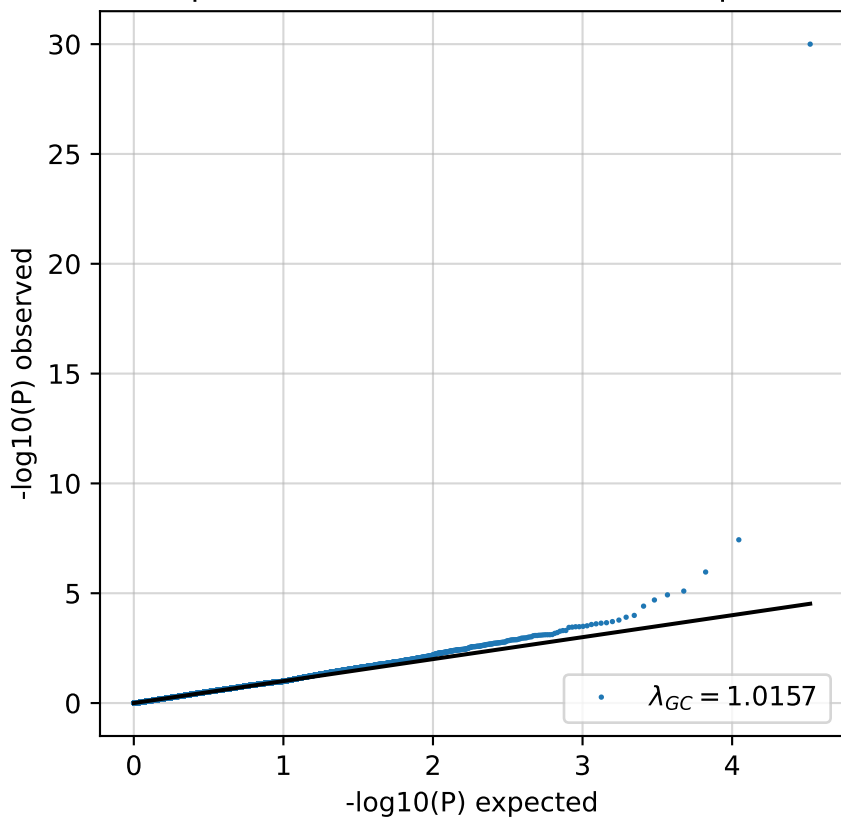

Supplement: Supplementary file 8 — Supplementary Data 5 [file 41467_2022_32864_MOESM8_ESM.zip › qqplots/splice_K_sLRT_Alanine_aminotransferase.pdf]

Albumin, test type: K,  
implementation: sLRT, var. effect: splice

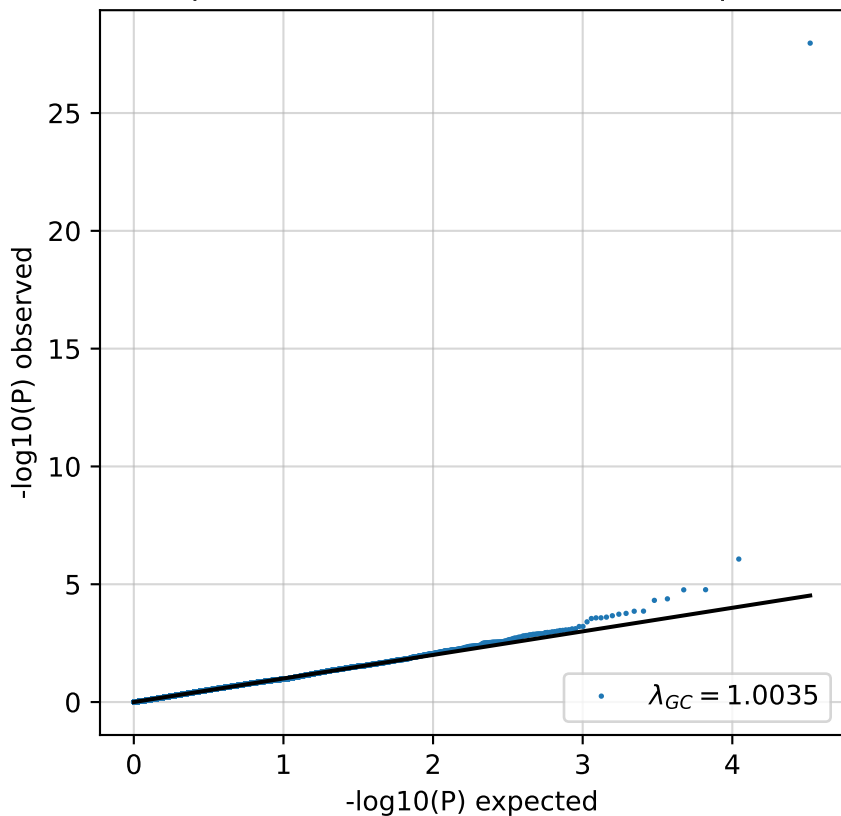

Supplement: Supplementary file 8 — Supplementary Data 5 [file 41467_2022_32864_MOESM8_ESM.zip › qqplots/splice_K_sLRT_Albumin.pdf]

Alkaline\_phosphatase, test type: K,  
implementation: sLRT, var. effect: splice

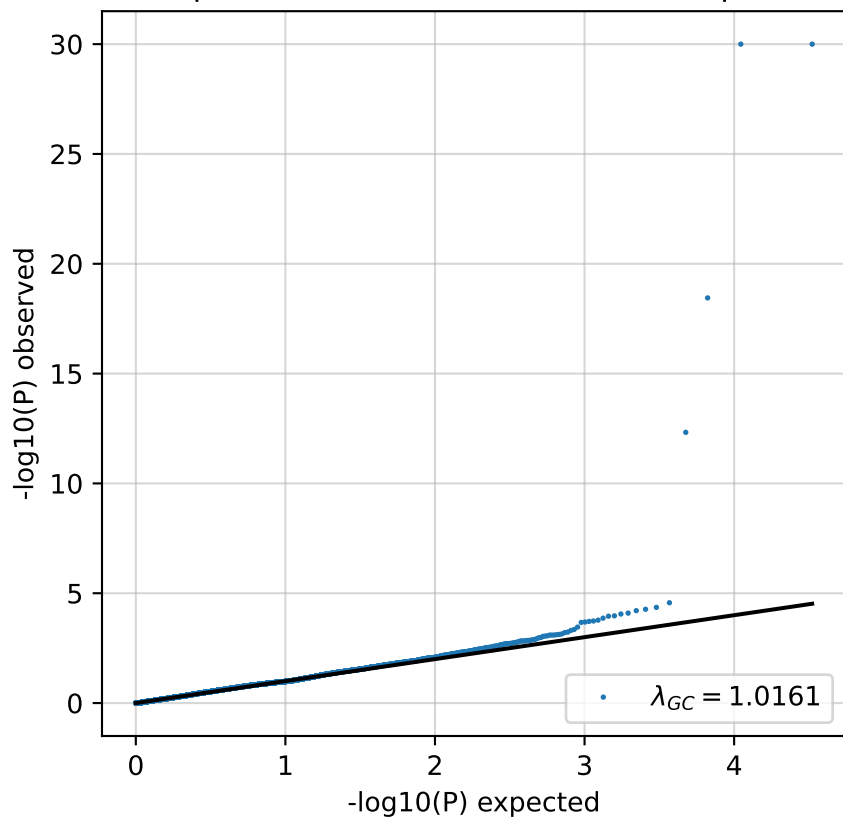

Supplement: Supplementary file 8 — Supplementary Data 5 [file 41467_2022_32864_MOESM8_ESM.zip › qqplots/splice_K_sLRT_Alkaline_phosphatase.pdf]

Apolipoprotein\_A, test type: K,  
implementation: sLRT, var. effect: splice

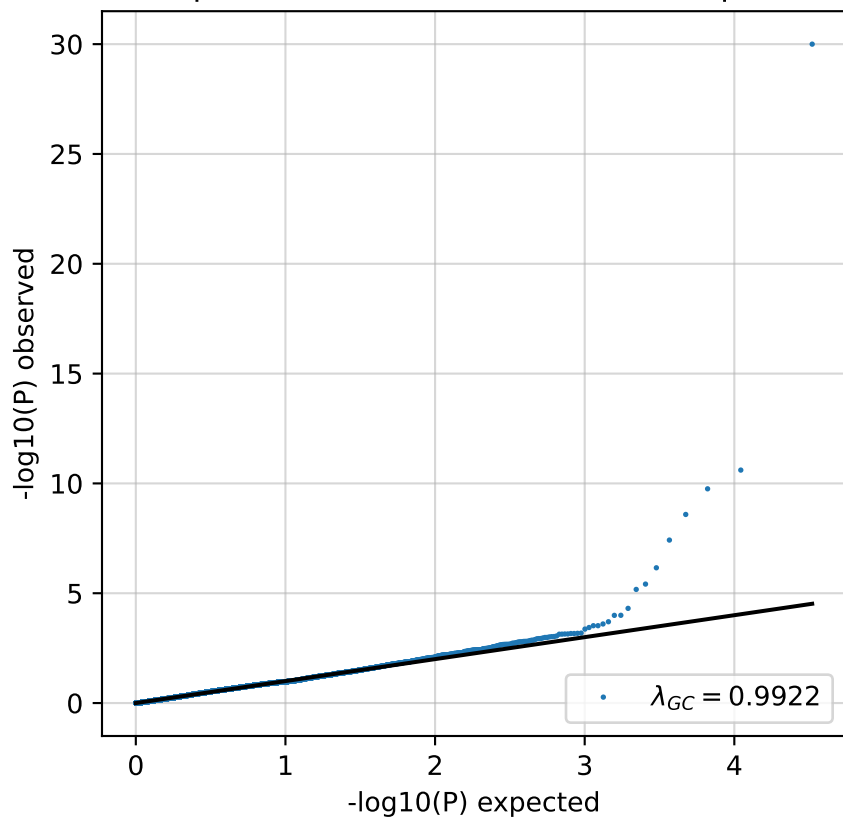

Supplement: Supplementary file 8 — Supplementary Data 5 [file 41467_2022_32864_MOESM8_ESM.zip › qqplots/splice_K_sLRT_Apolipoprotein_A.pdf]

Apolipoprotein\_B, test type: K,  
implementation: sLRT, var. effect: splice

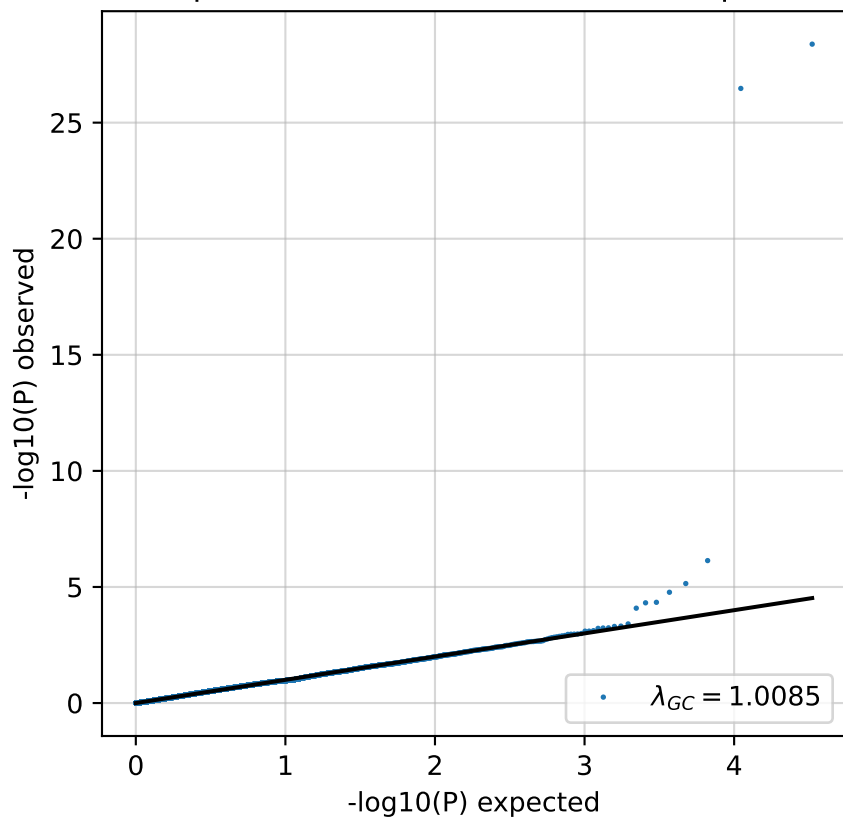

Supplement: Supplementary file 8 — Supplementary Data 5 [file 41467_2022_32864_MOESM8_ESM.zip › qqplots/splice_K_sLRT_Apolipoprotein_B.pdf]

Aspartate\_aminotransferase, test type: K,  
implementation: sLRT, var. effect: splice

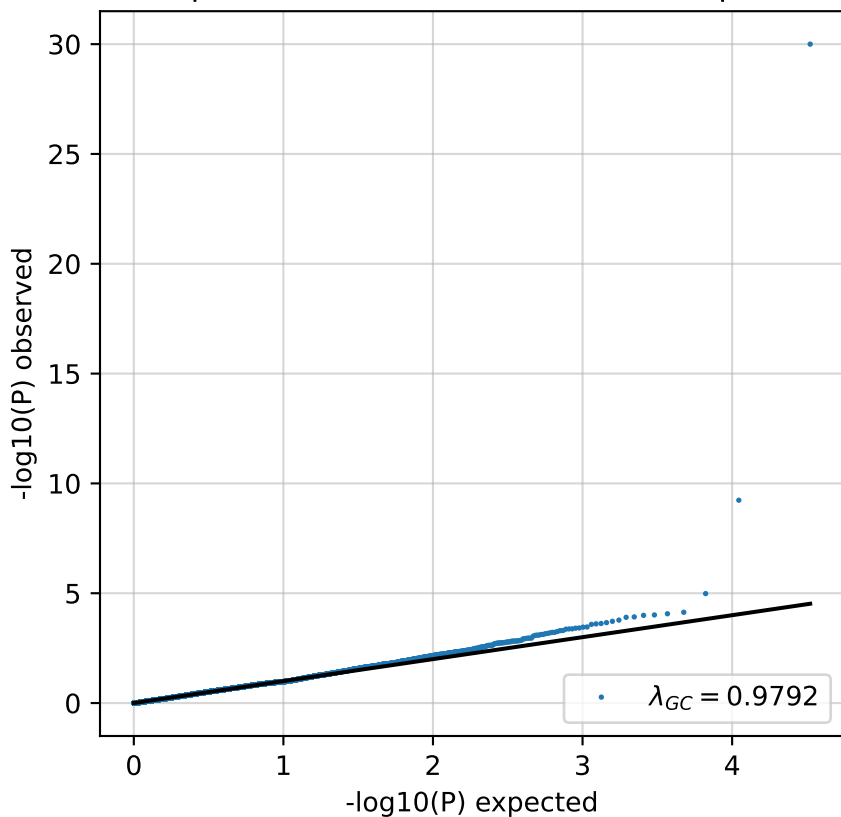

Supplement: Supplementary file 8 — Supplementary Data 5 [file 41467_2022_32864_MOESM8_ESM.zip › qqplots/splice_K_sLRT_Aspartate_aminotransferase.pdf]

Cholesterol, test type: K,  
implementation: sLRT, var. effect: splice

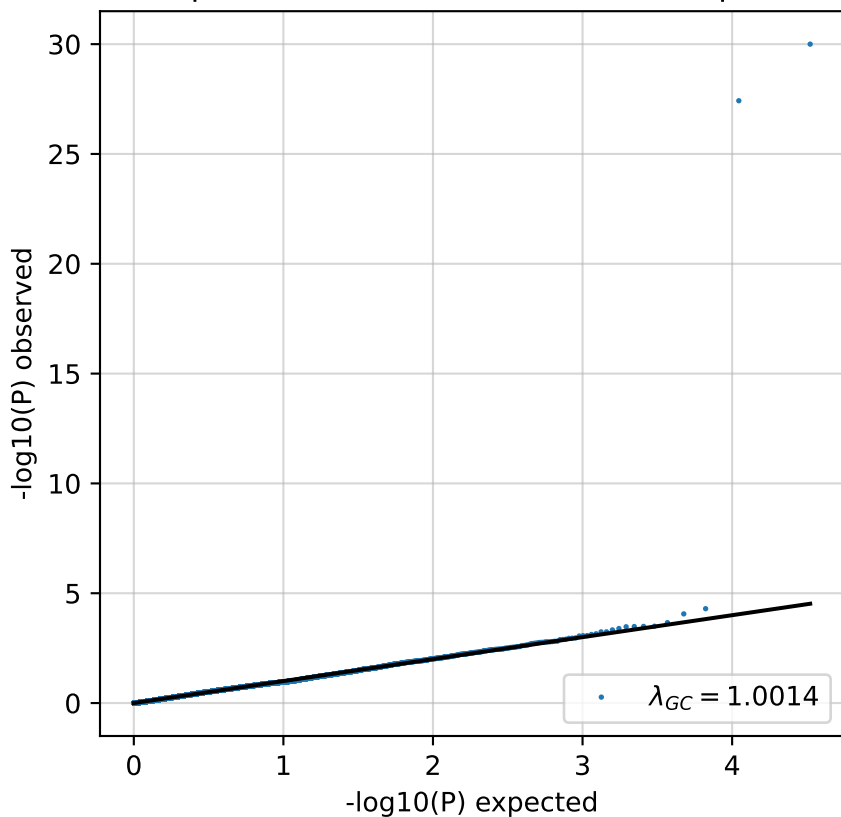

Supplement: Supplementary file 8 — Supplementary Data 5 [file 41467_2022_32864_MOESM8_ESM.zip › qqplots/splice_K_sLRT_Cholesterol.pdf]

Cystatin\_C, test type: K,  
implementation: sLRT, var. effect: splice

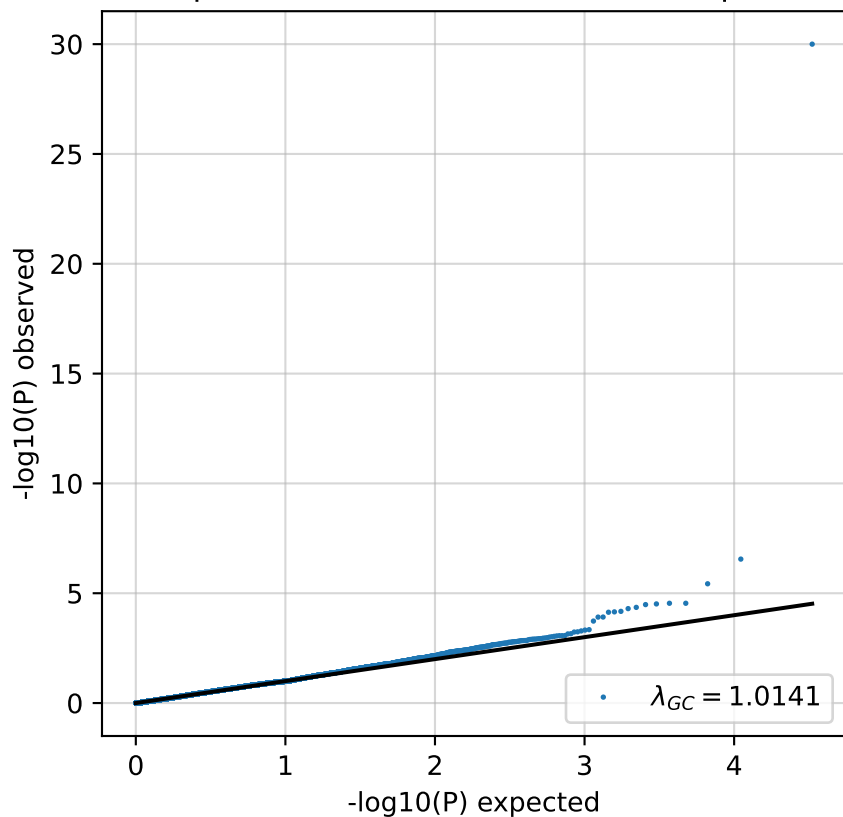

Supplement: Supplementary file 8 — Supplementary Data 5 [file 41467_2022_32864_MOESM8_ESM.zip › qqplots/splice_K_sLRT_Cystatin_C.pdf]

Gamma\_glutamyltransferase, test type: K,  
implementation: sLRT, var. effect: splice

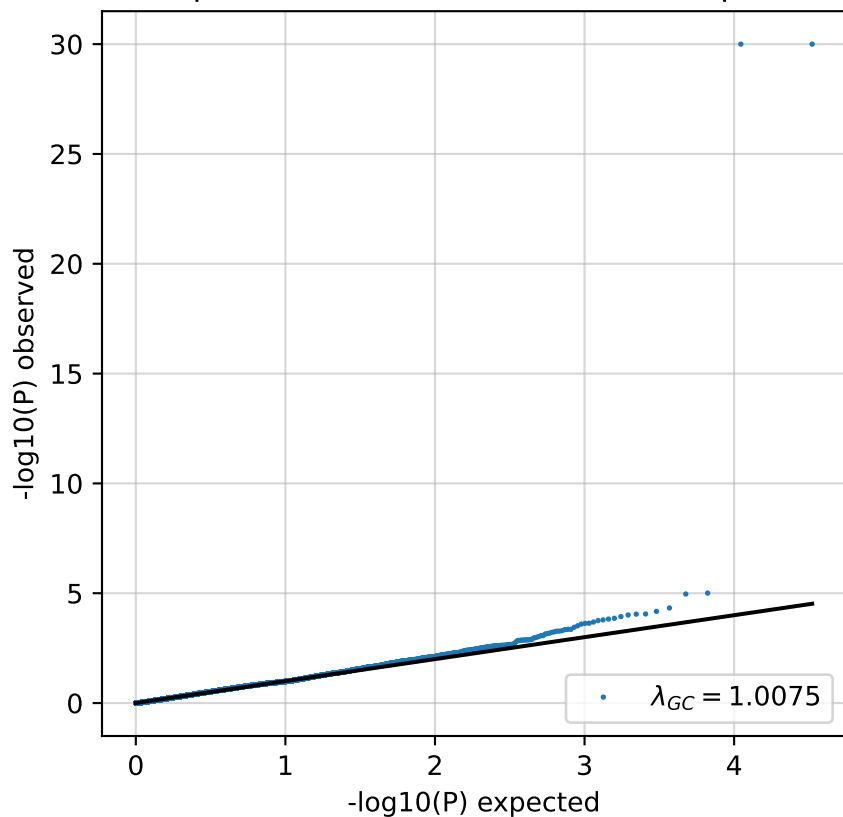

Supplement: Supplementary file 8 — Supplementary Data 5 [file 41467_2022_32864_MOESM8_ESM.zip › qqplots/splice_K_sLRT_Gamma_glutamyltransferase.pdf]

Glycated\_haemoglobin\_(HbA1c), test type: K,  
implementation: sLRT, var. effect: splice

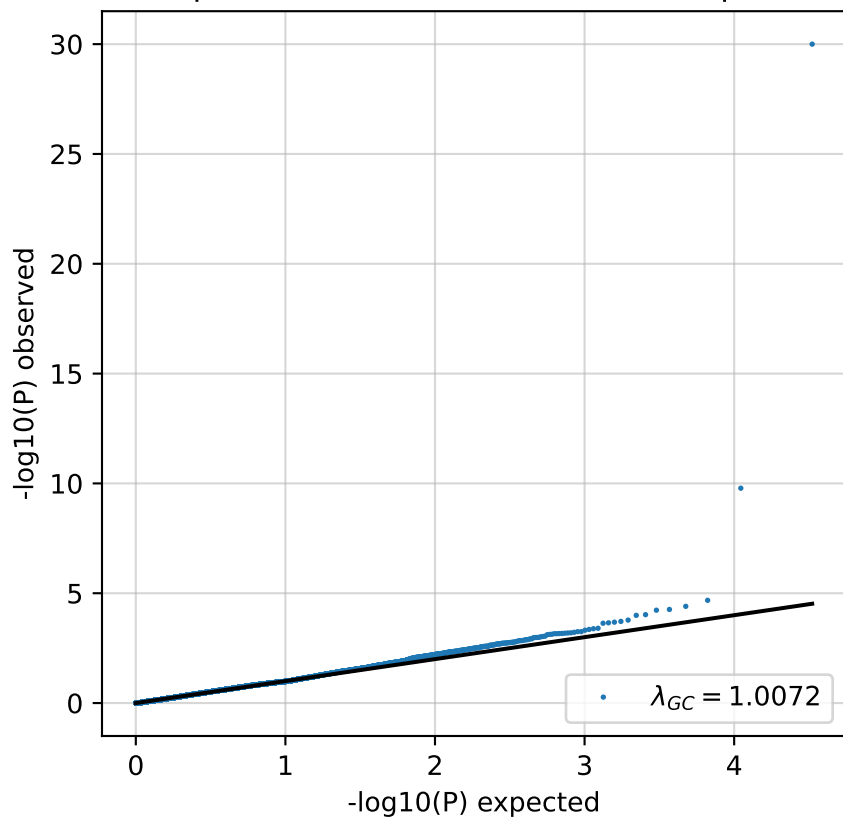

Supplement: Supplementary file 8 — Supplementary Data 5 [file 41467_2022_32864_MOESM8_ESM.zip › qqplots/splice_K_sLRT_Glycated_haemoglobin_HbA1c.pdf]

HDL\_cholesterol, test type: K,  
implementation: sLRT, var. effect: splice

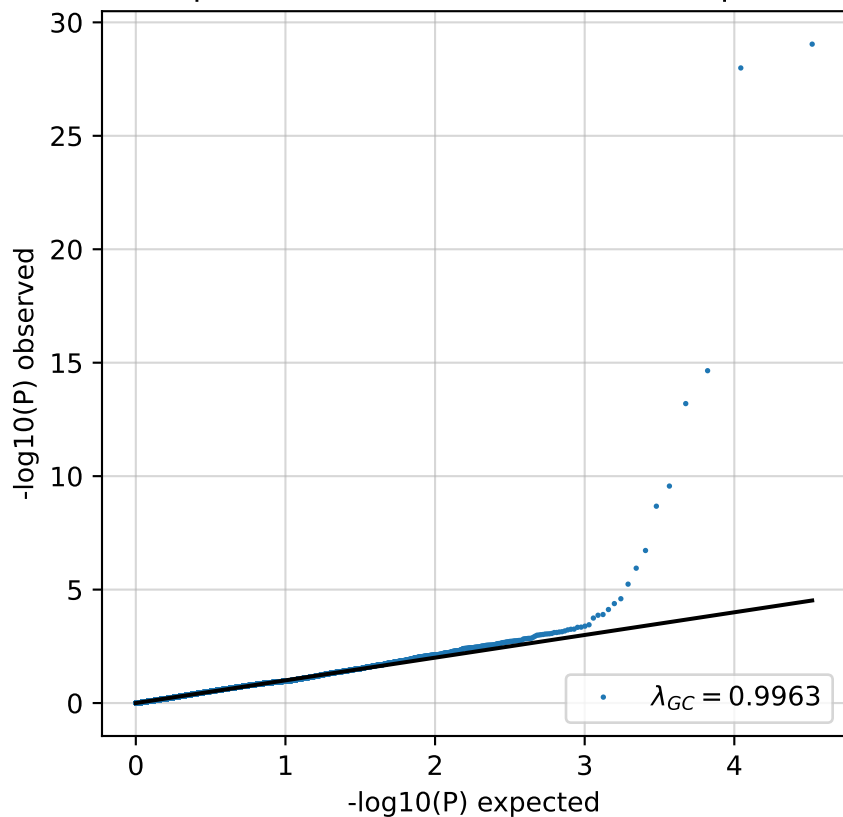

Supplement: Supplementary file 8 — Supplementary Data 5 [file 41467_2022_32864_MOESM8_ESM.zip › qqplots/splice_K_sLRT_HDL_cholesterol.pdf]

IGF-1, test type: K,  
implementation: sLRT, var. effect: splice

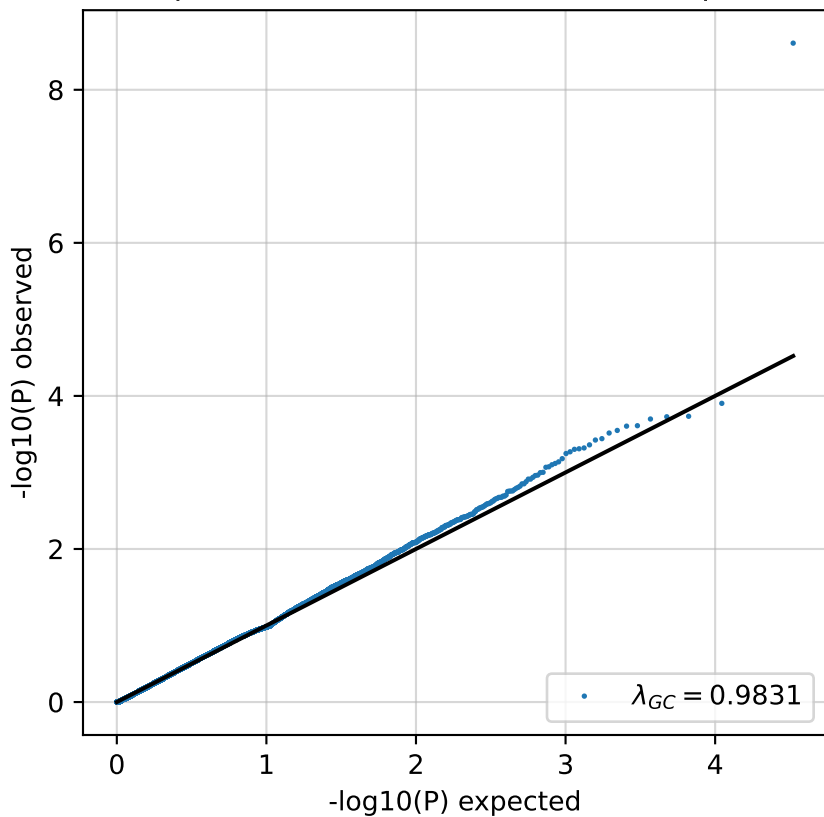

Supplement: Supplementary file 8 — Supplementary Data 5 [file 41467_2022_32864_MOESM8_ESM.zip › qqplots/splice_K_sLRT_IGF1.pdf]

LDL\_direct, test type: K,  
implementation: sLRT, var. effect: splice

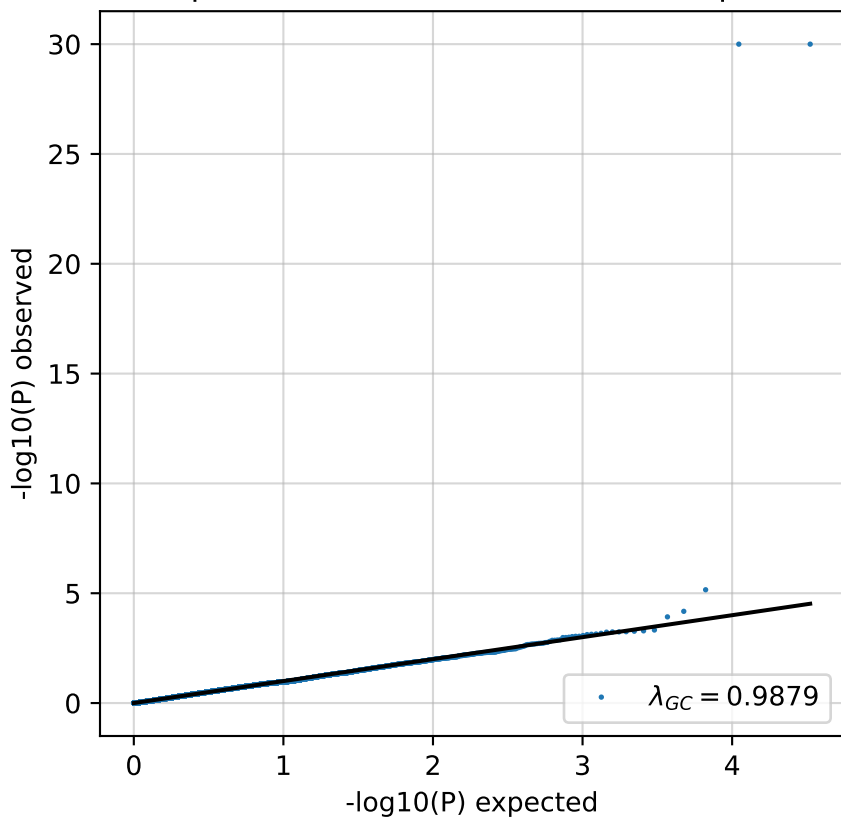

Supplement: Supplementary file 8 — Supplementary Data 5 [file 41467_2022_32864_MOESM8_ESM.zip › qqplots/splice_K_sLRT_LDL_direct.pdf]

Lipoprotein\_A, test type: K,  
implementation: sLRT, var. effect: splice

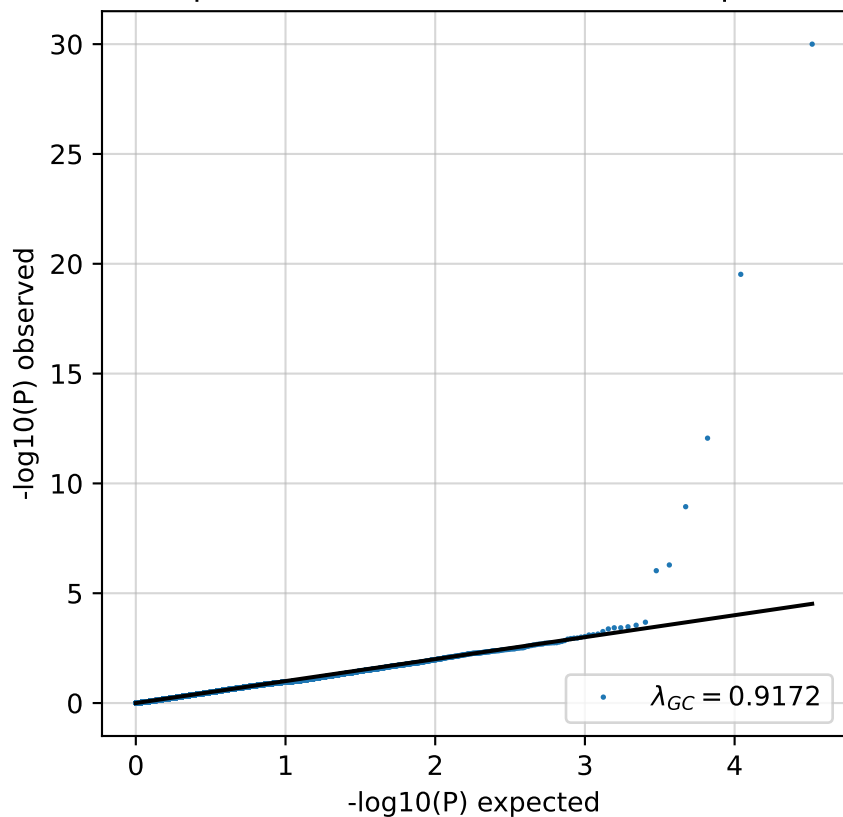

Supplement: Supplementary file 8 — Supplementary Data 5 [file 41467_2022_32864_MOESM8_ESM.zip › qqplots/splice_K_sLRT_Lipoprotein_A.pdf]

Phosphate, test type: K,  
implementation: sLRT, var. effect: splice

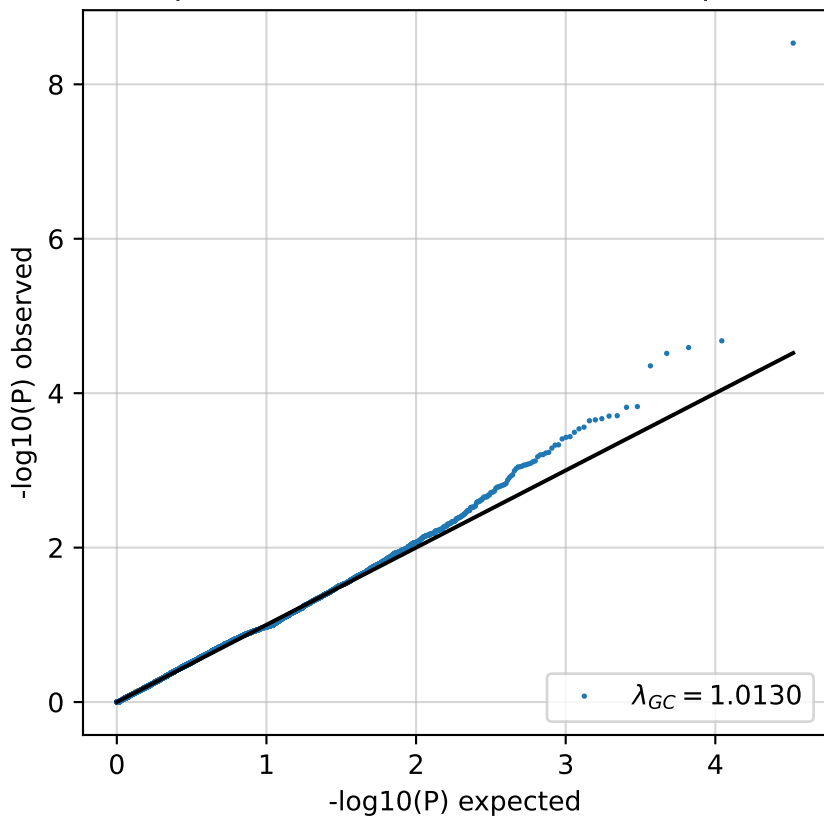

Supplement: Supplementary file 8 — Supplementary Data 5 [file 41467_2022_32864_MOESM8_ESM.zip › qqplots/splice_K_sLRT_Phosphate.pdf]

SHBG, test type: K,  
implementation: sLRT, var. effect: splice

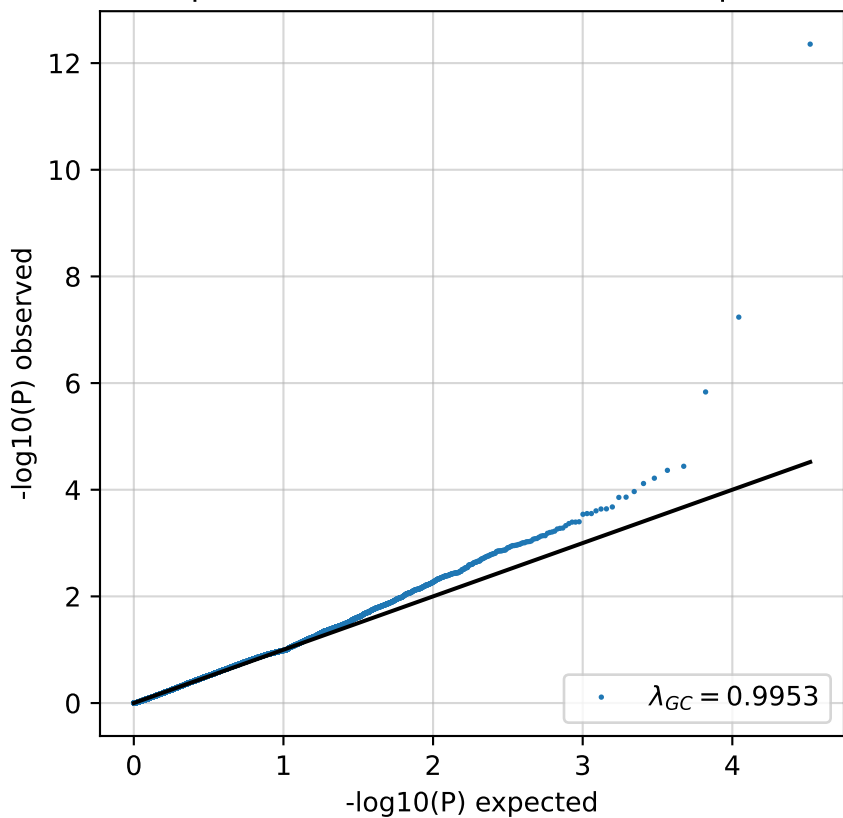

Supplement: Supplementary file 8 — Supplementary Data 5 [file 41467_2022_32864_MOESM8_ESM.zip › qqplots/splice_K_sLRT_SHBG.pdf]

Testosterone, test type: K,  
implementation: sLRT, var. effect: splice

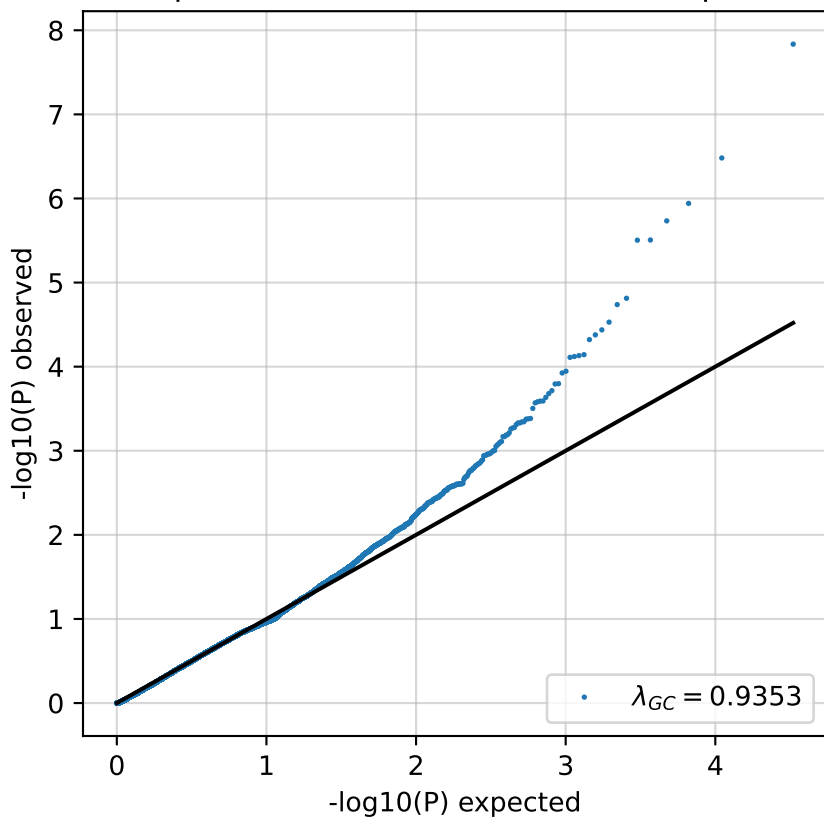

Supplement: Supplementary file 8 — Supplementary Data 5 [file 41467_2022_32864_MOESM8_ESM.zip › qqplots/splice_K_sLRT_Testosterone.pdf]

Total\_bilirubin, test type: K,  
implementation: sLRT, var. effect: splice

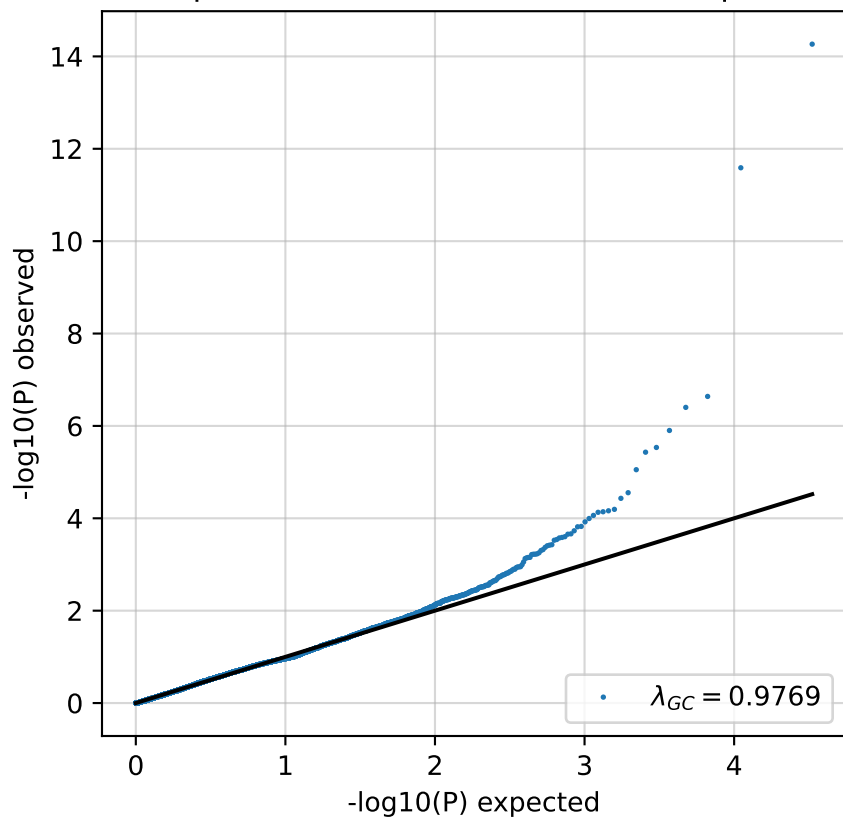

Supplement: Supplementary file 8 — Supplementary Data 5 [file 41467_2022_32864_MOESM8_ESM.zip › qqplots/splice_K_sLRT_Total_bilirubin.pdf]

Triglycerides, test type: K,  
implementation: sLRT, var. effect: splice

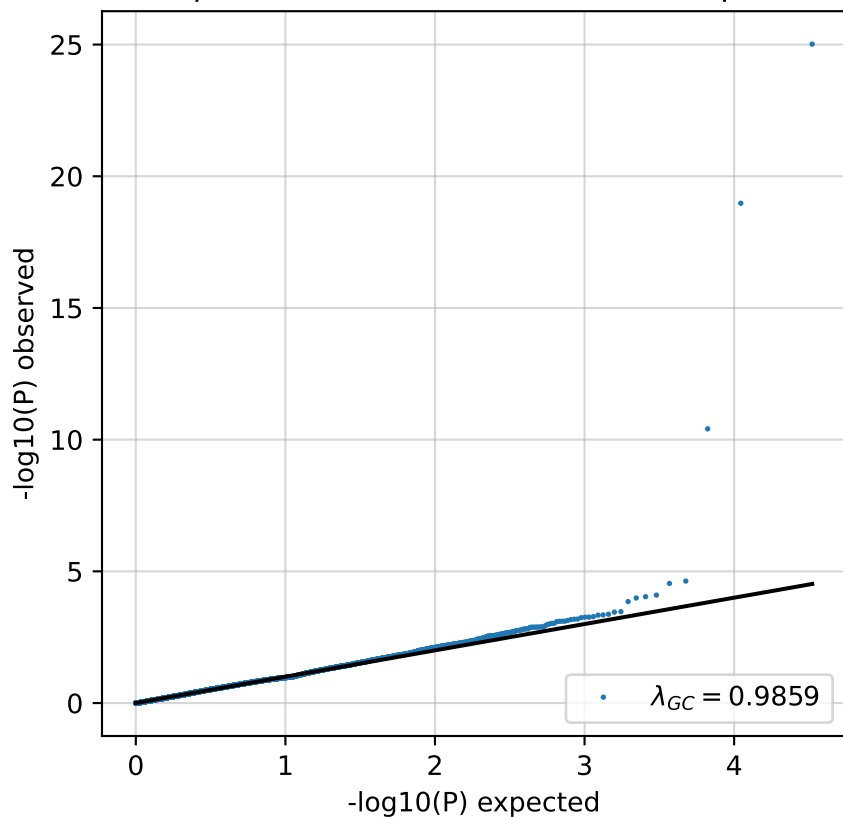

Supplement: Supplementary file 8 — Supplementary Data 5 [file 41467_2022_32864_MOESM8_ESM.zip › qqplots/splice_K_sLRT_Triglycerides.pdf]

Urate, test type: K,  
implementation: sLRT, var. effect: splice

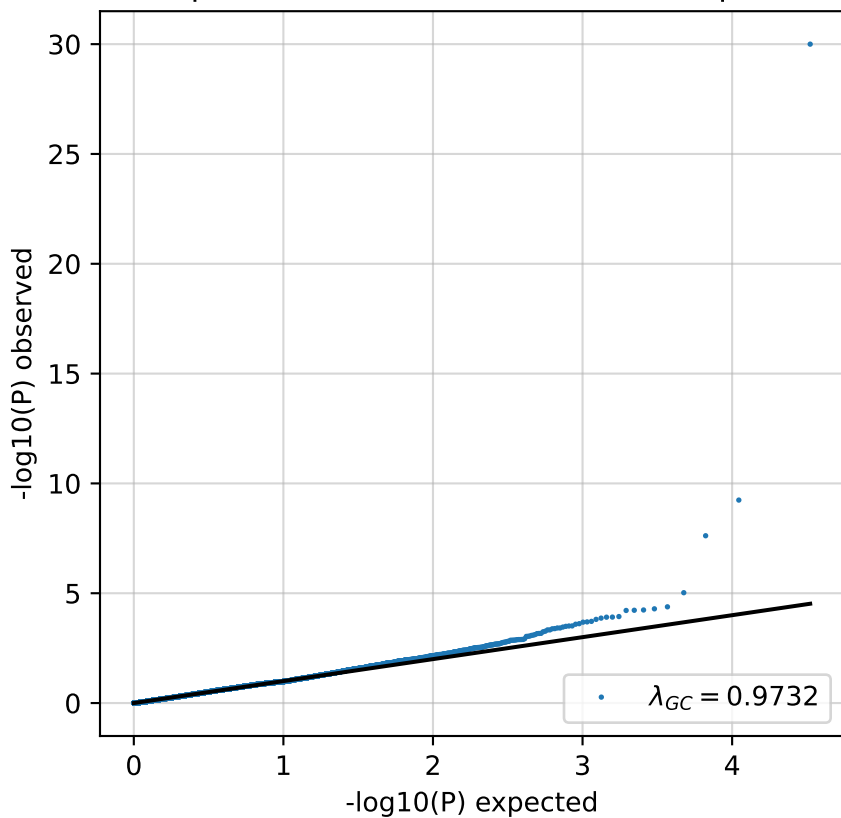

Supplement: Supplementary file 8 — Supplementary Data 5 [file 41467_2022_32864_MOESM8_ESM.zip › qqplots/splice_K_sLRT_Urate.pdf]
