# Supplementary material for: Identifying interpretable gene-biomarker associations with functionally informed kernel-based tests in 190,000 exomes
Source: Nat Commun. 2022 Sep 10;13:5332. doi: 10.1038/s41467-022-32864-2 (PMC9464252; doi:10.1038/s41467-022-32864-2)
Supplement: Supplementary file 8 — Supplementary Data 5 [file pLOF_gbvc_score_Gamma_glutamyltransferase.pdf › qqplots/pLOF_gbvc_score_Gamma_glutamyltransferase.pdf]

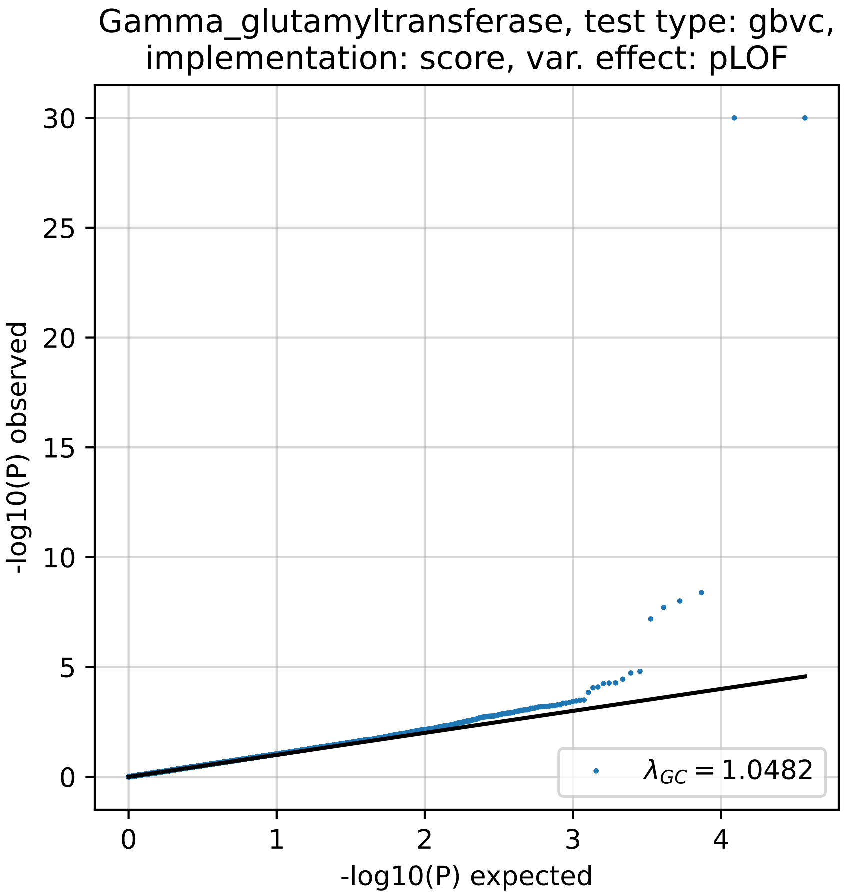

Gamma_glutamyltransferase, test type: gbvc,
implementation: score, var. effect: pLOF
-log10(P) observed
-log10(P) expected
0
5
10
15
20
25
30
0
1
2
3
4
$\lambda_{GC} = 1.0482$
