# Supplementary material for: Identifying interpretable gene-biomarker associations with functionally informed kernel-based tests in 190,000 exomes
Source: Nat Commun. 2022 Sep 10;13:5332. doi: 10.1038/s41467-022-32864-2 (PMC9464252; doi:10.1038/s41467-022-32864-2)
Supplement: Supplementary file 9 — Reporting Summary [file 41467_2022_32864_MOESM9_ESM.pdf]

nature portfolio

Corresponding author(s): Christoph Lippert

Last updated by author(s): Aug 3, 2022

Reporting Summary

Nature Portfolio wishes to improve the reproducibility of the work that we publish. This form provides structure for consistency and transparency in reporting. For further information on Nature Portfolio policies, see our Editorial Policies and the Editorial Policy Checklist.

Statistics

For all statistical analyses, confirm that the following items are present in the figure legend, table legend, main text, or Methods section.

n/a	Confirmed	
☐	☒	The exact sample size (*n*) for each experimental group/condition, given as a discrete number and unit of measurement
☒	☐	A statement on whether measurements were taken from distinct samples or whether the same sample was measured repeatedly
☐	☒	The statistical test(s) used AND whether they are one- or two-sided<br>*Only common tests should be described solely by name; describe more complex techniques in the Methods section.*
☐	☒	A description of all covariates tested
☐	☒	A description of any assumptions or corrections, such as tests of normality and adjustment for multiple comparisons
☐	☒	A full description of the statistical parameters including central tendency (e.g. means) or other basic estimates (e.g. regression coefficient) AND variation (e.g. standard deviation) or associated estimates of uncertainty (e.g. confidence intervals)
☐	☒	For null hypothesis testing, the test statistic (e.g. F, t, r) with confidence intervals, effect sizes, degrees of freedom and P value noted<br>*Give P values as exact values whenever suitable.*
☒	☐	For Bayesian analysis, information on the choice of priors and Markov chain Monte Carlo settings
☒	☐	For hierarchical and complex designs, identification of the appropriate level for tests and full reporting of outcomes
☐	☒	Estimates of effect sizes (e.g. Cohen's *d*, Pearson's *r*), indicating how they were calculated

Our web collection on statistics for biologists contains articles on many of the points above.

Software and code

Policy information about availability of computer code

Data collection	No data collection was performed. No software was used for data collection.

Data analysis	Phenotype pre-processing: python (3.8.2) with numpy (1.12.1), pandas (0.24.2), scikit-learn (0.22.2); R (3.6.3) with ukbtools (0.11.3).<br><br>Working with genetic intervals or genotype data: bcftools (1.11), bedtools (2.29.2), Plink (v1.90b6.21, v2.00a2.3LM), samtools (1.11), vcftools (0.1.16), htslib (1.11); python (3.6) with biopython (1.70), pybedtools (0.8.1), pyranges (0.0.88) and pysam (0.16.0.1)<br><br>Variant effect prediction: tensorflow (1.12.0), keras (2.2.4), Ensembl variant effect predictor - VEP (v101, cache version 97), Polyphen-2 (2.2.2), SIFT (5.2.2)<br><br>Statistical analysis: python (3.8.5) with seak (available at https://github.com/HealthML/seak, tag 0.4.3, https://doi.org/10.5281/zenodo.6912202), FaST-LMM (0.4.11), pysnptools (0.4.26)<br><br>Querying GWAS databases: R with gwasrapidd (0.99.11), phenoscanner (1.0)<br><br>Figures: ggplot2 (3.3.3), gplots (3.1.1), matplotlib (3.4.2), seaborn (0.11.2), matplotlib_venn (0.11.6)<br><br>Ancestry scoring: GenoPred (https://github.com/opain/GenoPred/, commit 4f8c63108ad3423327738df466742186ae114b58)<br><br>An analysis pipeline that allows reproducing the results is available at http://github.com/HealthML/faatpipe, tag 0.1.0, https://doi.org/10.5281/zenodo.6912198)

For manuscripts utilizing custom algorithms or software that are central to the research but not yet described in published literature, software must be made available to editors and reviewers. We strongly encourage code deposition in a community repository (e.g. GitHub). See the Nature Portfolio guidelines for submitting code & software for further information.

Data

Policy information about availability of data

All manuscripts must include a data availability statement. This statement should provide the following information, where applicable:
- Accession codes, unique identifiers, or web links for publicly available datasets
- A description of any restrictions on data availability
- For clinical datasets or third party data, please ensure that the statement adheres to our policy

Variant effect predictions generated in this study are made available at https://github.com/HealthML/ukb-200k-wes-vep (v0.0.0, https://doi.org/10.5281/zenodo.6912352).

Online Mendelian Inheritance in Man, OMIM®. https://omim.org/

ClinVar: https://www.ncbi.nlm.nih.gov/clinvar/

The NHGRI-EBI Catalog of human genome-wide association studies (GWAS catalog): https://www.ebi.ac.uk/gwas/

A database of human genotype-phenotype associations (phenoscanner): http://www.phenoscanner.medschl.cam.ac.uk/

SpliceAI variant effect predictions are available from Illumina: https://basespace.illumina.com/s/otSPW8hnhaZR

The genetic, phenotype and covariate data are protected and are only available to researchers that have valid and approved research applications for these data within the UK Biobank (www.ukbiobank.ac.uk/).

1000 Genomes phase3 v5: https://ftp.1000genomes.ebi.ac.uk/vol1/ftp/technical/working/20140708_previous_phase3/v5_vcfs/

Human research participants

Policy information about studies involving human research participants and Sex and Gender in Research.

Reporting on sex and gender	The reported sex of participants was accessed through UK-Biobank Data-Field 31, and included as a covariate in the statistical analyses.<br><br>The description of the Data-Field states:<br><br>"Acquired from central registry at recruitment, but in some cases updated by the participant. Hence this field may contain a mixture of the sex the NHS had recorded for the participant and self-reported sex."<br>- https://biobank.ctsu.ox.ac.uk/crystal/field.cgi?id=31, accessed on the 28th July 2022<br><br>Out of the 191,971 participants included in the analyses in this study, 105,549 were reported as having female sex, and 86,422 were reported as having male sex.<br><br>Gender was not considered as a variable in this study.
Population characteristics	The mean age at recruitment of the 191,971 participants included in the statistical analyses in this study was 56.5 years, standard deviation 8.076, and ranged from 38 to 72 years. The average BMI in this sample was 27.37 (standard deviation 4.757). Based on an ancestry prediction model implemented using the 1000 Genomes data and superpopulation assignments (Methods), the sample was determined to consist predominantly of individuals with "EUR" ancestry (95%), followed by

	"SAS" (2.2%), "AFR" (2%), "EAS" (0.6%) and "AMR" (0.15%).
Recruitment	The UK Biobank recruited approximately 500,000 individuals in 2006 to 2010 with a target age of 40-69 by mailers to people in the UK medical system. Informed consent was obtained by the UK Biobank for all participants.
Ethics oversight	The scientific protocol of the UK Biobank is approved from appropriate external ethics committees in accordance with guidance from relevant bodies. Instead of requiring each applicant to obtain separate ethics approval, UK Biobank has sought generic Research Tissue Bank (TB) approval, which covers the large majority of research using the resource.<br><br>The original approval for the UK Biobank was granted in 2011 by the National Research Ethics Service (NRES) Committee North West - Haydock. The approval was renewed in 2016 and 2021 by the Health Research Authority, North West - Haydock Research Ethics Committee.<br><br>For additional information, see https://www.ukbiobank.ac.uk/learn-more-about-uk-biobank/about-us/ethics.<br><br>This research has been conducted using the UK Biobank Resource under Application Number 40502, "Association tests for structured and multimodal data"

Note that full information on the approval of the study protocol must also be provided in the manuscript.

Field-specific reporting

Please select the one below that is the best fit for your research. If you are not sure, read the appropriate sections before making your selection.

☒ Life sciences ☐ Behavioural & social sciences ☐ Ecological, evolutionary & environmental sciences

For a reference copy of the document with all sections, see nature.com/documents/nr-reporting-summary-flat.pdf

Life sciences study design

All studies must disclose on these points even when the disclosure is negative.

Sample size	Sample sizes were determined from the data. We did not pre-define sample size based on power. The sample size for each phenotype analyzed in this study was determined by the number of individuals which had both complete (i.e., non-missing) phenotype and covariate data. Because of varying levels of missingness for the different phenotypes, the sample size ranged from 15,997 to 182,742. All phenotypes were continuous
Data exclusions	We removed individuals who have withdrawn consent and retained only 1 individual of groups of individuals related to the third degree or less. These exclusions are already reflected in the sample sizes reported above.
Replication	The vast majority of significant associations replicated associations previously reported to GWAS databases (Phenoscanner, GWAS catalog) and were well aligned with knowledge available through GeneCards, ClinVar, UniProt and/or OMIM. Overlaps with other rare-variant association studies were assessed, and indicated high reproducibility within UK Biobank (Supplementary Data 1).<br><br>Statistical replication of gene-based tests in independent exome-sequencing data could not be performed, as no comparable data were available to us. The association of PIEZO1 L2277M with HbA1c-levels in individuals of inferred South Asian ancestry was replicated once within an independent subset of the data consisting of individuals of inferred European ancestry, as described in the Methods.
Randomization	We did not allocate samples into experimental groups.
Blinding	No groups were allocated.

Reporting for specific materials, systems and methods

We require information from authors about some types of materials, experimental systems and methods used in many studies. Here, indicate whether each material, system or method listed is relevant to your study. If you are not sure if a list item applies to your research, read the appropriate section before selecting a response.

Materials & experimental systems

n/a	Involved in the study
☒	☐ Antibodies
☒	☐ Eukaryotic cell lines
☒	☐ Palaeontology and archaeology
☒	☐ Animals and other organisms
☒	☐ Clinical data
☒	☐ Dual use research of concern

Methods

n/a	Involved in the study
☒	☐ ChIP-seq
☒	☐ Flow cytometry
☒	☐ MRI-based neuroimaging
